# Mono- and bithiophene-substituted diarylethene photoswitches with emissive open or closed forms

**DOI:** 10.3762/bjoc.15.227

**Published:** 2019-10-01

**Authors:** A Lennart Schleper, Mariano L Bossi, Vladimir N Belov, Stefan W Hell

**Affiliations:** 1 Department of NanoBiophotonics, Max Planck Institute for Biophysical Chemistry, 37077 Göttingen, Germany; 2 Department of Organic Chemistry, Heidelberg University, Im Neuenheimer Feld 270, 69120 Heidelberg, Germany; 3 Department of Optical Nanoscopy, Max Planck Institute for Medical Research, Jahnstrasse 29, 69120 Heidelberg, Germany

**Keywords:** diarylethenes, dyes, fluorescence, organic synthesis, photochromism, photoswitching

## Abstract

We present a new series of photochromic 1,2-bis(2-ethylbenzo[*b*]thiophen-3-yl)perfluorocyclopentenes with an oxidized benzothiophene core (O) or a nonoxidized one, decorated with mono- (Th_1_) and bithiophene (Th_2_) units attached to positions 6 and 6′ (Sy = symmetric) or only to position 6 (As = asymmetric). “Oxidized” compounds have highly fluorescent closed forms emitting in the visible region (yellow to red). The dyes with nonoxidized benzothiophenes possess fluorescent open forms with rather low emission efficiency. The photoswitching kinetics was studied at several wavelengths with UV and visible light. New diarylethenes underwent ring-closure reactions by irradiation with UV light (365 nm, 405 nm), and the reversible ring-opening by irradiation with visible light (470 nm, 530 nm). The on-switching of fluorescence due to the ring-closure reaction was observed also with visible light of 470 nm (to an extent of 10% for compound **SyOTh_1_**) and attributed to the Urbach tail effect. Due to a high degree of fluorescence modulation (>270), good fatigue resistance and large fluorescence quantum yield, compound **SyOTh_1_** emerged as a candidate for single-molecule based super-resolution fluorescence microscopy.

## Introduction

Reversibly photoswitchable diarylethenes (DAEs) with highly fluorescent “closed” forms combine photochromic and fluorescent entities in one molecule [1] and contain a perfluorocyclopentene bridge linking two 2-alkyl-1-benzothiophene-1,1-oxide residues with a C=C bond via C-3 and C-3′ atoms [2–3]. The “open” form of the DAE core (see graphical abstract and Table 1) is only weakly fluorescent (φ_Fl_ ≈ 0.01) [4]. In general, diarylethenes with fluorescent open forms are rare, while photoswitchable compounds with highly fluorescent “open” *and* “closed” forms represent a yet unknown and unique class of ratiometric fluorophores.

**Figure 1:**
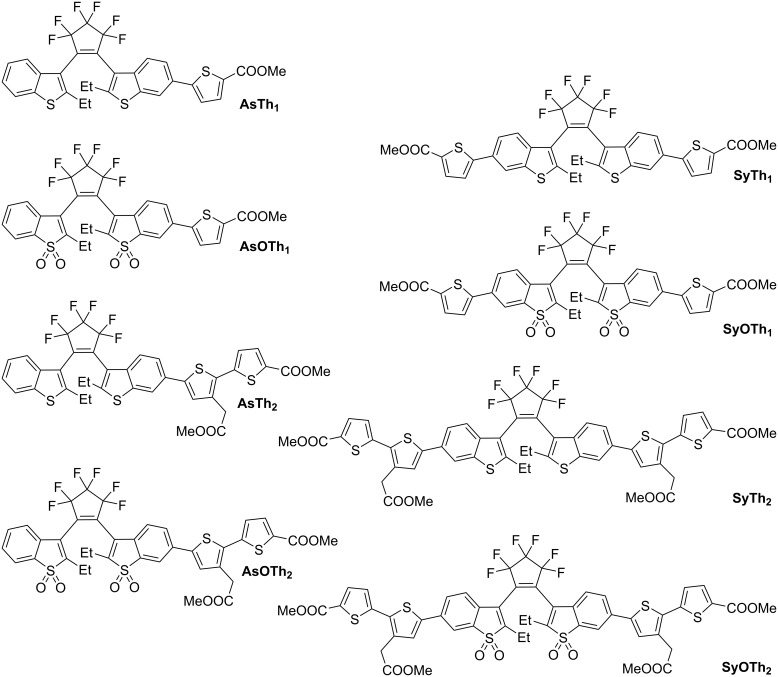
Structures of “thiophenylated” DAEs prepared and studied in this work.

Recently, we demonstrated that asymmetric DAEs with oxidized 2-ethylbenzo[*b*]thiophene-3-yl units have high cycloreversion quantum yields (a desirable feature for super-resolution RESOLFT microscopy), large Stokes shifts and acceptable absorptivity [5]. Yet unknown asymmetric “thiophenylated” DAEs may have unique properties. Oligothiophenes are highly fluorescent [6–7], and therefore, we reasoned that their incorporation into DAEs with oxidized 2-alkylbenzo[*b*]thiophene units might produce fluorescent open forms in addition to the intrinsic fluorescence of the closed forms. In particular, we expected that the prolonged conjugation path, even at “one side” of the molecule (in asymmetric compounds) would enable switching with focusable light of 375 nm and above. To test this hypothesis, we designed a series of DAEs with mono- and bithiophene substituents attached to positions 6 and 6′ of an oxidized or a nonoxidized 2-alkylbenzo[*b*]thiophene core. The structures of “thiophenylated” DAEs **AsTh_1_**, **AsTh_2_**, **SyTh_1_**, **SyTh_2_**, **AsOTh_1_**, **AsOTh_2_**, **SyOTh_1_**, and **SyOTh_2_** prepared and studied in this work are given in Figure 1.

Methyl esters of the thiophene carboxylic acids were chosen as model building blocks, because they possess optical properties similar to the free acids. The future promising candidates which were expected to emerge in the course of synthesis and screening could be eventually transformed to the corresponding carboxylic acids possessing higher solubility in aqueous buffers and reactive groups required for bioconjugation.

## Results and Discussion

### Synthetic procedures

**Scheme 1:**
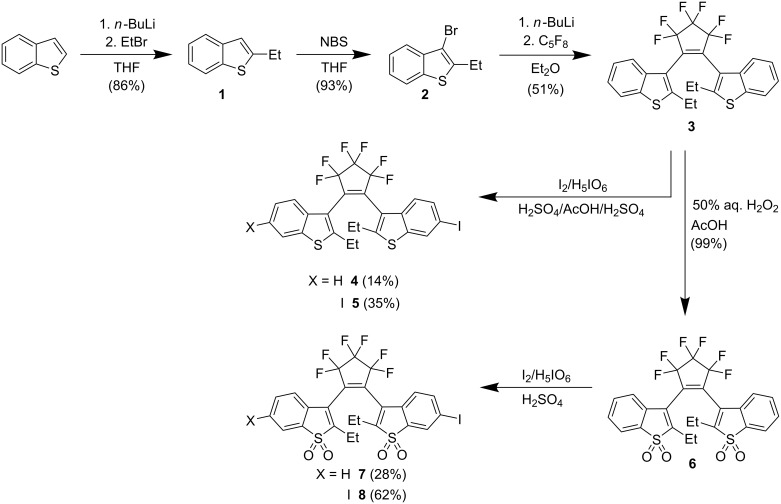
Synthesis routes towards mono- and diiodinated core structures **4**, **5**, **7**, and **8**.

**Scheme 2:**
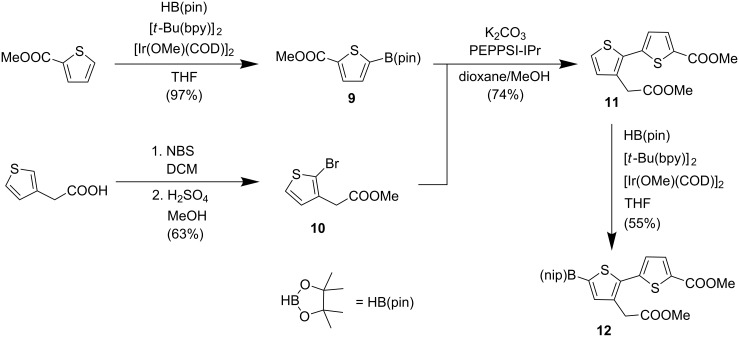
Synthesis of thiophene- and bithiopheneboronic esters **9** and **12** (bpy – 4,4’-di-*tert*-butyl-2,2’-dipyridine; COD – cycloocta-1,5-diene; NBS – *N*-bromosuccinimide, DCM – dichloromethane).

To enable a facile synthesis of oxidized and nonoxidized 1,2-bis(2-ethylbenzo[*b*]thiophen-3-yl)perfluorocylopentenes decorated with thiophene units, mono- and diiodinated core structures **4**, **5**, **7**, and **8** (Scheme 1), as well as mono- and bithiopheneboronic esters **9** and **12** (Scheme 2) were prepared.

The transformations leading to diarylethene cores **3** and **6** were carried out on a 10–100 mmol scale (Scheme 1). The iodination at positions 6 and 6' of benzo[*b*]thiophene residues has been reported [5,8]. Following those protocols, diiodides **5** and **8** were obtained in fairly good yields by treatment of **3** with elemental iodine and periodic acid in aqueous acetic acid with addition of sulfuric acid, and by treatment of **6** with elemental iodine and periodic acid in concentrated sulfuric acid, respectively. The conditions for monoiodination of **6** have been published recently [5]. We observed that the reproducible monoiodination of DAE **3** to form compound **4** was possible under similar conditions and in low yield (14%) by using smaller amounts of periodic acid (0.34 equiv) and iodine (0.68 equiv) than the amounts of these reagents used for diiodination (see Experimental for details). The progress of the reaction leading to monoiodide **4** was monitored by HPLC. Despite incomplete conversion, the formation of diiodide **5** could not be fully suppressed. The *R*_f_ values of **3** (TLC [SiO_2_, hexane]: *R*_f_ = 0.17), monoiodide **4** (*R*_f_ = 0.19), and diiodide **5** (*R*_f_ = 0.20) were very similar, and DAE **4** could not be isolated by column chromatography on regular silica gel. Instead, monoiodide **4** was obtained in 14% yield as colorless solid after preparative HPLC on a reversed phase (C18) column and lyophilization. The constitution and structure of **4** was confirmed by HRMS, ^1^H and ^19^F NMR spectroscopy (see Figure S1 in Supporting Information File 1). These results show that “desymmetrization” of DAEs still represents a real synthetic challenge. Diiodides **5** and **8** provide the possibility [9–10] to obtain symmetric DAEs, and monoiodides **4** and **7** allow for a short and straightforward approach towards asymmetric DAEs.

The thiophene- and bithiopheneboronic esters **9** and **12** were prepared as shown in Scheme 2. C–H activation of methyl thiophene-2-carboxylate in position 5 was achieved by using a bis(1,5-cyclooctadiene)di-μ-methoxydiiridium(I) catalyst with 4,4’-di-*tert*-butyl-2,2’-dipyridyl as ligands. The reaction with pinacolborane (HB(pin)) gave ester **9** in 97% yield [11]. Another ester (**10**) was synthesized by bromination of 3-thiopheneacetic acid with NBS (applying an ultrasound bath) followed by esterification of the crude product with methanol in the presence of sulfuric acid [12]. Other regioisomeric bromides were also formed, but they were separated by column chromatography on the next step. Bithiophene **11** was prepared in 74% yield by using a Suzuki–Miyaura coupling (catalyzed by PEPPSI-IPr) between boronic ester **9** and bromide **10** [13–14]. Boronation of bithiophene **11** was achieved under conditions similar to the preparation of thiophene **9** and afforded ester **12** in 55% yield (79% crude yield with 70% purity). However, in this case, larger amounts of catalyst and pinacolborane were required for good conversions.

**Scheme 3:**
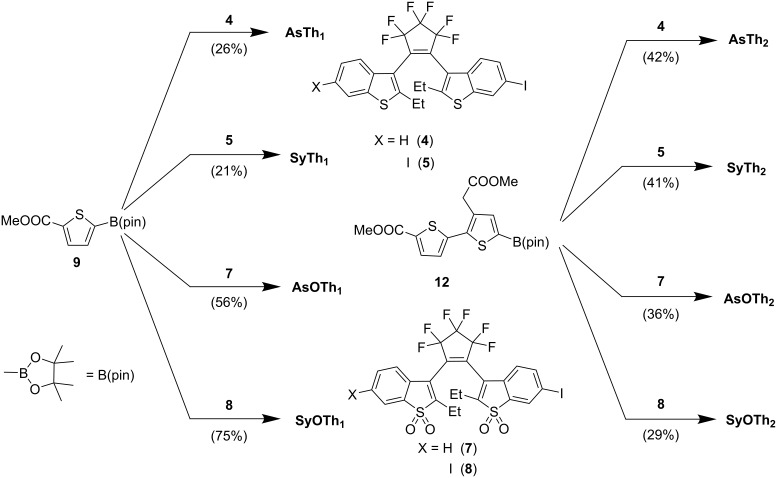
Photoswitchable diarylethenes **AsTh_1_**, **SyTh_1_**, **AsTh_2_**, **SyTh_2_**, **AsOTh_1_**, **SyOTh_1_**, **AsOTh_2_**, and **SyOTh_2_** synthesized via a Suzuki–Miyaura coupling. Conditions: 60 °C, argon atmosphere, an emulsion of the starting compounds, Pd_2_(dba)_3_, P(Cy)_3_ stock solution, and aqueous K_2_CO_3_ in THF (see Supporting Information File 1 for details).

Finally, the photoswitchable DAEs **AsTh_1_**, **SyTh_1_**, **AsTh_2_**, **SyTh_2_**, **AsOTh_1_**, **SyOTh_1_**, **AsOTh_2_**, and **SyOTh_2_** (Figure 1) were obtained using a standard procedure for a Suzuki–Miyaura coupling of iodides **4**, **5**, **7**, and **8** with boronic acid esters **9** and **12** (see Scheme 3). Throughout the text, abbreviations “As” and “Sy” denote asymmetric and symmetric substitution patterns, respectively; “O” indicates oxidized benzothiophene units, and “Th_1_”/ “Th_2_” specify the number of thiophene units in the side chain. The coupling products were isolated via preparative HPLC in yields ranging from 21% to 75%. While in all cases the open forms of the diarylethenes were isolated, small amounts of **SyOTh_1_** in its closed form (formed by handling in the lab not fully protected from light) were detected by HPLC (Figure S33 in Supporting Information File 1). The constitution, structures, and purities of the final products were confirmed by HRMS, ^1^H and ^19^F NMR spectroscopy, as well as analytical HPLC.

**Scheme 4:**
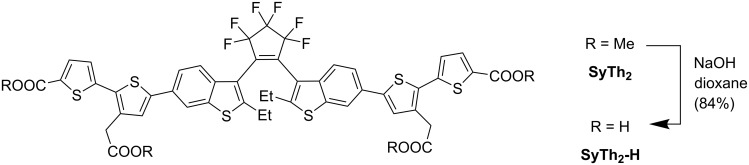
Saponification of methyl ester groups in tetraester **SyTh_2_** leading to tetracarboxylic acid **SyTh_2_-H**.

Saponification of the ester groups was performed only for nonoxidized benzothiophenes, as shown for compound **SyTh_2_** in Scheme 4. Diarylethenes with “oxidized” benzothiophene units and perfluorocyclopentene rings react with aqueous or alcoholic solutions of strong bases [5,10,15]. Under these conditions, substitution of fluorine and/or the addition of nucleophiles to the electron-poor double bond take place. Therefore, *tert-*butyl protection is required for carboxylates present in diarylethenes with “oxidized” benzothiophene units and perfluorocyclopentene rings. Photoswitches containing the free carboxylic acid residues benefit from solubility in aqueous buffers and the presence of a reactive group required for further modifications. The methyl groups were cleaved using 1 M aq NaOH, and the product **SyTh_2_-H** was isolated by preparative HPLC in 84% yield (see Scheme 4). As shown in Figure S2 (see Supporting Information File 1), tetraester **SyTh_2_** displayed six ^1^H NMR signals between 3.70 and 4.00 ppm, belonging to two inequivalent methyl ester groups and the methylene group of the parallel and antiparallel isomers. After the reaction, four of the six signals vanished, and only two methylene signals (two isomers) were detected, confirming cleavage of the methyl esters.

### Photophysical properties

**Figure 2:**
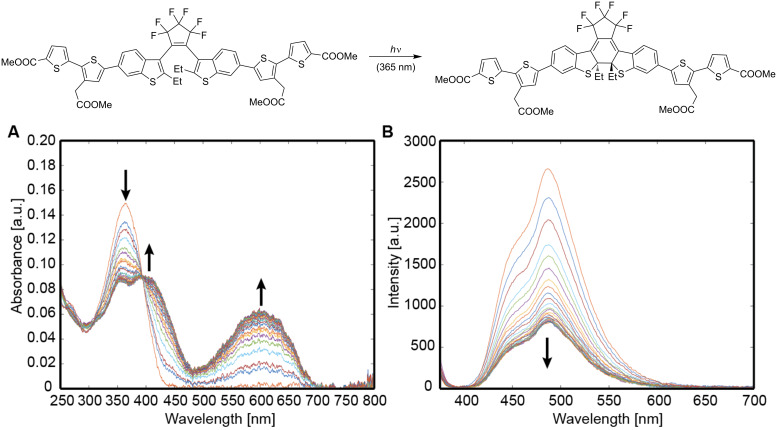
Absorption (A) and emission (B) spectra of **SyTh_2_** in acetonitrile in the course of the cyclization reaction (irradiation at 365 nm). Emission was not fully depleted due to incomplete conversion in the photostationary state (PSS, α = 71%).

The absorption and emission properties as well as the photochromic properties of eight “thiophenylated” DAEs were studied in acetonitrile solutions (Table 1). The properties of the OFs (open forms) were obtained from diluted solutions at a known concentration prior to irradiation. Solutions were irradiated in a home-made setup [5] with 355 nm light until the photostationary state (PSS_365 nm_). Figure 2 shows changes of absorption and emission spectra in the course of the switching process for compound **SyTh_2_**. Irradiated solutions in the PSS_365 nm_ were analyzed by HPLC at the isosbestic point. Conversion to the CF (closed form) α_PSS_ = [CF]/([CF] + [OF]) was calculated from the proportion of the area corresponding to the CF (area_CF_/[area_CF_ + area_OF_]). The absorption spectrum of the CF was calculated from the absorption spectrum at the PSS_365 nm_ and the absorption spectrum of the OF, using the obtained conversion. The obtained spectra for all CF’s matched, within experimental errors, the absorption recorded on the diode array detector of the HPLC, at the center of the elution peak of the corresponding isomer (see for example, Figures S26 and S38 in Supporting Information File 1). Emission properties of the CF’s were obtained from solutions at the PSS_365 nm_, with excitation at a wavelength at which the OF’s do not absorb (only the CF absorbs). The data on switching kinetics is given in Table 2 and discussed in the next section.

**Table 1:** Optical properties of the photochromic DAEs (in acetonitrile solution).

	Absorption					Emission						Stokes shift [nm] (cm^−1^)
	OF^a^		CF^a^		Separation^b^ [nm] (cm^-1^)	OF			CF			
	λ_abs_ [nm]	ε/10^3^ [M^−1^cm^−1^]	λ_abs_ [nm]	ε/10^3^ [M^−1^cm^−1^]		λ_em_^c^ (λ_ex_) [nm]	φ_Fl_^d^	τ_Fl_^e^ [ns]	λ_em_^c^ (λ_ex_) [nm]	φ_Fl_^d^	τ_Fl_^e^ [ns]	

**AsTh_1_**	327	25.8	562	13.5	235 (12800)	410 (320)	0.01	0.20	–	–	–	83 (6190)
**SyTh_1_**	328	50.6	584	21.3	256 (13400)	410 (340)	0.01	0.23	–	–	–	82 (6100)
**AsTh_2_**	363	20.0	566	12.1	203 (9880)	462 (360)	0.09	0.24	–	–	–	99 (5900)
**SyTh_2_**	363	63.1	591	32.5	228 (10600)	469 (410)	0.08	0.24	–	–	–	106 (6230)
**AsOTh_1_**	356	15.5	447	37.1	91 (5720)	–	–	–	550 (460)	0.36	1.63	103 (4190)
**SyOTh_1_**	355	31.4	478	53.5	123 (7250)	–	–	–	560 (470)	0.48	1.91	82 (3060)
**AsOTh_2_**	385	36.4	480	58.6	95 (5140)	–	–	–	656 (460)	0.16	1.28	176 (5590)
**SyOTh_2_**	384	47.9	524	63.8	140 (6960)	–	–	–	670 (530)	0.18	1.16	146 (4160)
**3**^f^ [16]	258	16.5	536	9.6	278 (20100)	–	–	–	–	–	–	–
**6** [4]	310	5.3	412	23.7	102 (7990)	464	0.01	0.26^g^ 5.04^h^	509	0.06	0.51	154^i^ (10700)^i^ 97^j^ (4630)^j^

^a^Wavelength and extinction coefficient at the absorption maxima. ^b^Separation between absorption maxima of OF and CF. ^c^Position of the emission maxima (excitation wavelength). ^d^Fluorescence quantum yield. ^e^Fluorescence lifetime. ^f^In hexane solution. ^g^Parallel isomer. ^h^Antiparallel isomer. ^i^OF (open form). ^j^CF (closed form).

**Figure 3:**
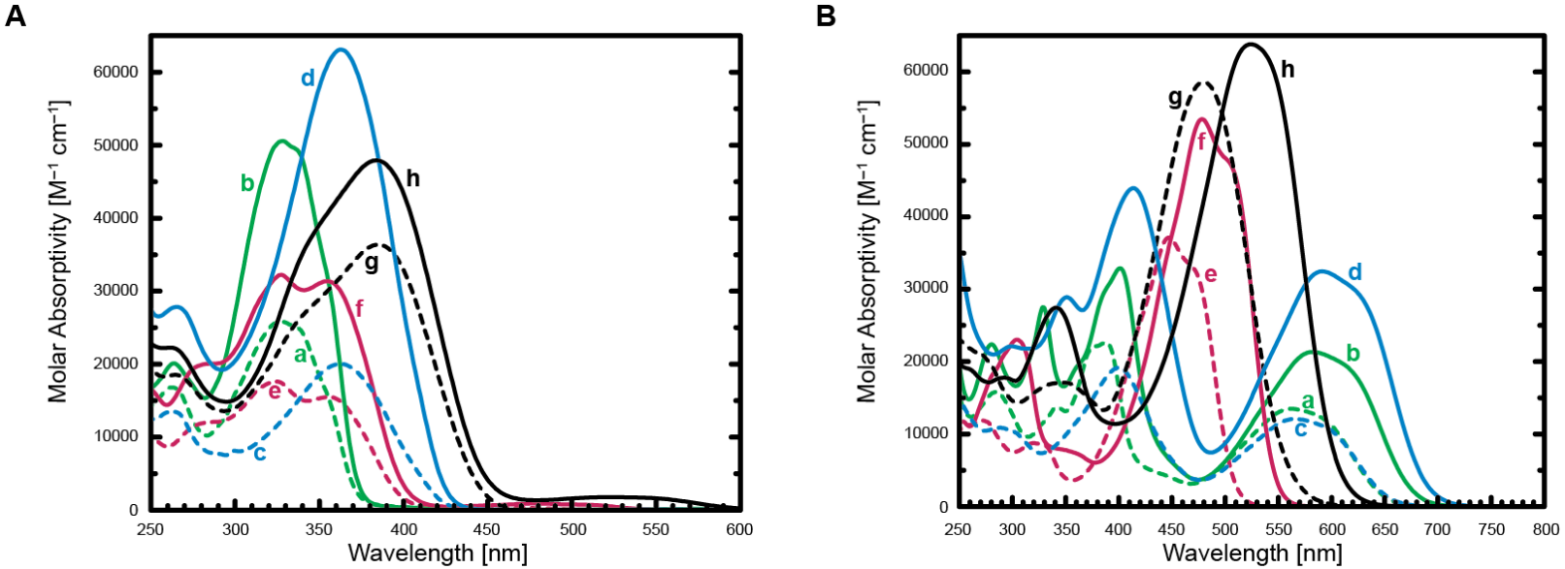
Absorption spectra of the OFs (A) and CFs (B) of **AsTh_1_** (a), **SyTh_1_** (b), **AsTh_2_** (c), **SyTh_2_** (d), **AsOTh_1_** (e), **SyOTh_1_** (f), **AsOTh_2_** (g), and **SyOTh_2_** (h). The absorption of the OFs in (A) above 460 nm is due to the presence of low amounts of the corresponding CFs (formed during manipulation of the solids and/or preparation of the solutions).

We first analyzed the absorption spectra of the open forms (see Figure 3A) in more detail. Addition of thiophene rings to the core structures **3** and **6** induced a red-shift in the OF (Table 1) [4,16]. Their absorption maxima were located between 327 nm and 385 nm, and present interesting correlations. First, the positions of the main absorption bands of the corresponding symmetric and asymmetric DAEs were very similar. The symmetric DAEs have higher absorption coefficients than their asymmetric counterparts (by a factor of 1.3–3.2). Considering the very similar band positions, this indicates that in the OF each benzothiophene unit (with aligning thiophene group(s)) contributes into absorption independently. It was confirmed that asymmetric compounds with “compact” structures can be transformed to the closed form with the same light as bulkier analogs (the latter featuring slower ring-opening reactions; see Table 2). Second, a bithiophene residue induces a red-shift of 30–35 nm in the absorption of the OF with respect to a mono-thiophene ring. The oxidized DAEs (OF) absorbed at higher wavelengths than the corresponding nonoxidized analogs (by ca. 20–30 nm), and a splitting of the band is observed with a shoulder appearing at the blue edge. In general, substitution in the OF with a bithiophene side chain induced a slightly larger red-shift than oxidation of benzothiophene units.

The absorption maxima of the CFs were located between 447 nm and 591 nm; they present remarkably larger differences than the corresponding OFs (see Figure 3B). Compared with the parent compounds **3** and **6** [4,16], the CFs of all new DAEs displayed, again, red-shifted absorption bands. Unlike for the OFs (Figure 3A), oxidation produces a strong blue shift of the absorption bands of the CF (compare the curves a–d and e–h in Figure 3B). This shift is stepwise reduced from 124 nm in the unsubstituted compounds (**3**→**6**), to 65 nm in the presence of four thiophene residues (**SyTh_2_**→**SyOTh_2_**). It is worth noting that for asymmetric compounds (**AsTh_2_**→**AsOTh_2_**), the shift (20 nm) is smaller than for symmetric DAEs having the same total number of thiophene rings (**SyTh_1_**→**SyOTh_1_**). All CFs showed several absorption bands, while nonoxidized DAEs have two distinct absorption bands in the visible range. The data in Figure 3 indicate that, contrary to the OF, both benzo[*b*]thiophene moieties (with side rings) interact and determine the location of the absorption band in the CF. If we compare the absorption spectra of **SyOTh_1_** and **AsOTh_2_**, we see that the most red-shifted bands have very similar positions. This is a remarkable feature, as both compounds consist of the same parts: two oxidized benzothiophenes and, in total, two thiophenes as substituents. It appears that the sequence of the building blocks is less important than their nature and quantity. The same regularity holds true (with less precision) for the most red-shifted absorption bands of **SyTh_1_** and **AsTh_2_** (CFs have 18 nm difference in band positions); both DAEs consisting of same building blocks, again!

If we consider the positions of the absorption bands of the OF and CF of each oxidized DAE, we observe that they are separated “only” by 90–140 nm. On the other hand, in nonoxidized DAEs, these bands are separated by 200–260 nm. This large difference is justified by the fact that the oxidation red-shifts the band positions of the OF, and blue-shifts the band positions of the CF. A conversion of the separation between the absorption bands from wavelengths to wavenumbers allows the evaluation of the relative energy gaps between the HOMO and LUMO. We can see that the energy gap between HOMO and LUMO in nonoxidized DAEs is decreased to a higher extent upon cyclization than in oxidized DAEs. Furthermore, we noted that asymmetric DAEs have a smaller separation between absorption maxima of OF and CF than their symmetric counterparts. We observed that nonoxidized DAEs exhibited a higher extinction coefficient in the OF than in the CF (by a factor of 1.7–2.4); oxidized DAEs revealed an opposite trend (higher absorption in the CF by a factor of 1.3–2.4).

**Figure 4:**
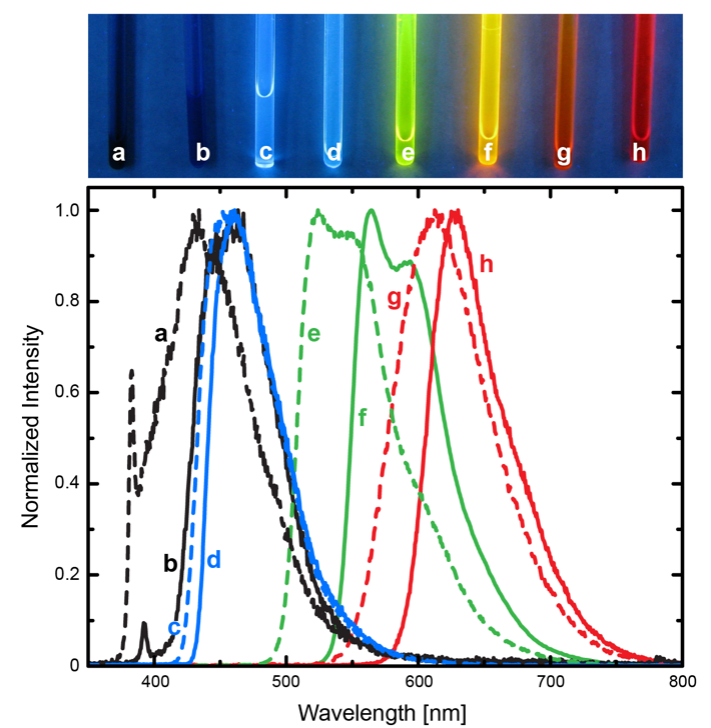
Solutions of compounds **AsTh_1_** (a), **SyTh_1_** (b), **AsTh_2_** (c), **SyTh_2_** (d), **AsOTh_1_** (e), **SyOTh_1_** (f), **AsOTh_2_** (g), and **SyOTh_2_** (h) in CDCl_3_ as mixtures of OFs and CFs under irradiation with 366 nm (top), and the corresponding emission spectra in MeCN (bottom). Emission of the compounds covers the range from UV to the blue and red regions. Sharp peaks at ca. 390 nm (a and b) are Raman bands of the solvent.

New DAEs have very different emission properties (see Figure 4 and Table 1). the oxidized DAEs strongly emit in their CFs, and the nonoxidized DAEs emit in their OFs, but rather weakly. Interestingly, unsubstituted DAEs **3** and **6** (Scheme 1) were either nonemissive at all (**3**), or very poorly emissive in both forms (**6**) [4,16]. The new DAEs – when nonoxidized – possessed blue emission with maxima between 410 nm and 470 nm. In this case, the difference in emission between asymmetric and symmetric substitution patterns was negligible. This feature can be explained by the fact that due to the twisted conformation of the open form, the emission transition involves the isolated benzothiophene unit (including the side ring). As expected, in MeCN quantum yields of nonoxidized compounds with a bithiophene side chain were higher (ca. 8% compared to 1% for a monothiophene derivative), but still moderate. Fluorescence lifetimes were short (0.2 ns), in accordance with moderate quantum yields.

The oxidized compounds were strongly fluorescent in their closed forms, with emission maxima in the range between 550 nm and 670 nm. The difference between the positions of emission bands of the structurally related symmetric and asymmetric compounds was small, compared to the change caused by an additional thiophene ring to the benzothiophene core. Oxidized DAEs with monothiophene units as side chains exhibited higher fluorescence quantum yields than the structurally related DAEs with bithiophenes (>35% compared to <20%). Longer fluorescent lifetimes (1.3–1.9 ns) of the oxidized DAEs (CFs) correspond to the better fluorescence quantum yields. They exceeded lifetimes measured for the fluorescence of the unsubstituted DAE **6** by a factor of 4, and the fluorescence quantum yield of the CF of **6** was surpassed by the oxidized DAEs with thiophene side rings by a factor of 3–8 [4]. The open forms of the substituted oxidized compounds were nonemissive.

The Stokes shift of the OFs of nonoxidized DAEs was always ca. 6000 cm^−1^, independent of the substitution pattern. For comparison, this value was much lower than the Stokes shift of the OF of compound **6** (10,700 cm^−1^) [4]. The Stokes shift of the CFs of oxidized DAEs varied between 3000 cm^−1^ and 5600 cm^−1^, which is comparable with the Stokes shift observed for the CF of **6** (4600 cm^−1^) [4]. In the case of oxidized DAEs, asymmetric compounds possessed higher Stokes shifts by ca. 1000 cm^−1^ than symmetric DAEs. Only compound **AsOTh_2_** has a higher Stokes shift than the CF of unsubstituted DAE **6**.

Two important observations were made in this part of the work. First, the OFs of oxidized DAEs absorb at higher wavelengths, and their CFs absorb at lower wavelengths than the corresponding isomers of the nonoxidized counterparts. Second, the CFs of nonoxidized DAEs were found to be nonemissive. This important property will be taken into account in the design of the future photoswitchable compounds with ratiometric fluorescence modulation.

### Switching kinetics

Table 2 contains an overview of the most important parameters related to the switching kinetics.

**Table 2:** Switching kinetics of the photochromic compounds (in acetonitrile solution).

	α^a^ [%]	φ_OF→CF_^b^	φ_CF→OF_^c^	φ_on-switching/_φ_off-switching_	*N*_ph_^d^

**AsTh_1_**	36	0.13	0.14	1.1^e^	<1
**SyTh_1_**	71	0.16	0.07	0.4^e^	<1
**AsTh_2_**	28	0.03	0.15	5.0^e^	3
**SyTh_2_**	71	0.04	0.06	1.5^e^	2
**AsOTh_1_**	>98	9.0 · 10^−2^	1.6 · 10^−3^	56^f^	225
**SyOTh_1_**	>98	2.4 · 10^−2^	8.8 · 10^−5^	273^f^	5230
**AsOTh_2_**	>98	7.6 · 10^−5^	–^g^	–	–
**SyOTh_2_**	>99	1.9 · 10^−4^	–^g^	–	–
**3**^h^ [16]	53^i^	0.39^i^	0.35^j^	–	–
**6** [4]	31	0.24^i^	0.25^k^	–	–

^a^Conversion into the CF in the PSS (365 nm). ^b^Irradiation at 365 nm. ^c^Irradiation at 530 nm. ^d^The number of average emitted photons before off-switching is proportional to the ratio of fluorescence quantum yield and off-switching quantum yield; the off-switching quantum yield is φ_OF→CF_ for nonoxidized and φ_CF→OF_ for oxidized compounds. ^e^Ratio of cycloreversion and cyclization. ^f^Ratio of cyclization and cycloreversion. ^g^Irreversible bleaching overcame the cycloreversion reaction. ^h^In hexane. ^i^Irradiation at 313 nm. ^j^Irradiation at 517 nm. ^k^Irradiation at 405 nm.

All compounds, except **AsOTh_2_** and **SyOTh_2_**, were reversibly switchable between open and closed forms by irradiation with 365 nm (cyclization) and 470 nm (cycloreversion) light. For compounds **AsOTh_2_** and **SyOTh_2_**, irreversible photobleaching was the main reaction path observed after irradiation with visible (470–530 nm) light (see Figures S55 and S61 in Supporting Information File 1). Considering the trend for other DAEs [1,5,9–10], we expect the cycloreversion quantum yields of compounds **AsOTh_2_** and **SyOTh_2_** to be <10^−6^–10^−7^, which means that the bleaching quantum yields are on the order of >10^−5^–10^−6^. The nonoxidized DAEs were also switchable by irradiation of the OF at 530 nm. In general, nonoxidized DAEs switched faster in both directions than the oxidized DAEs (by 1–4 orders of magnitude). Furthermore, the nonoxidized compounds (apart from **SyTh_1_**) revealed higher cycloreversion quantum yields (φ_CF→OF_) than cyclization quantum yields (φ_OF→CF_); the oxidized compounds showed an opposite trend. As a result, the degree of conversion in the photostationary state found for nonoxidized compounds was moderate <70% (ca. 30% for asymmetric and 70% for symmetric dyes), but the oxidized analogs show complete conversions (>98%). Interestingly, decoration of the core structures **3** and **6** (Scheme 1) with thiophene rings lowered the switching quantum yields by several orders of magnitude. It became obvious that the rate of cyclization and cycloreversion reactions decreased when the conjugation path became longer (transition from the unsubstituted core to monothiophene and then to bithiophene derivatives). In all cases, asymmetric substitution allowed faster cycloreversion than symmetric substitution, which is a positive effect regarding potential application of these dyes as reversible fluorescent markers in RESOLFT microscopy [5].

**Figure 5:**
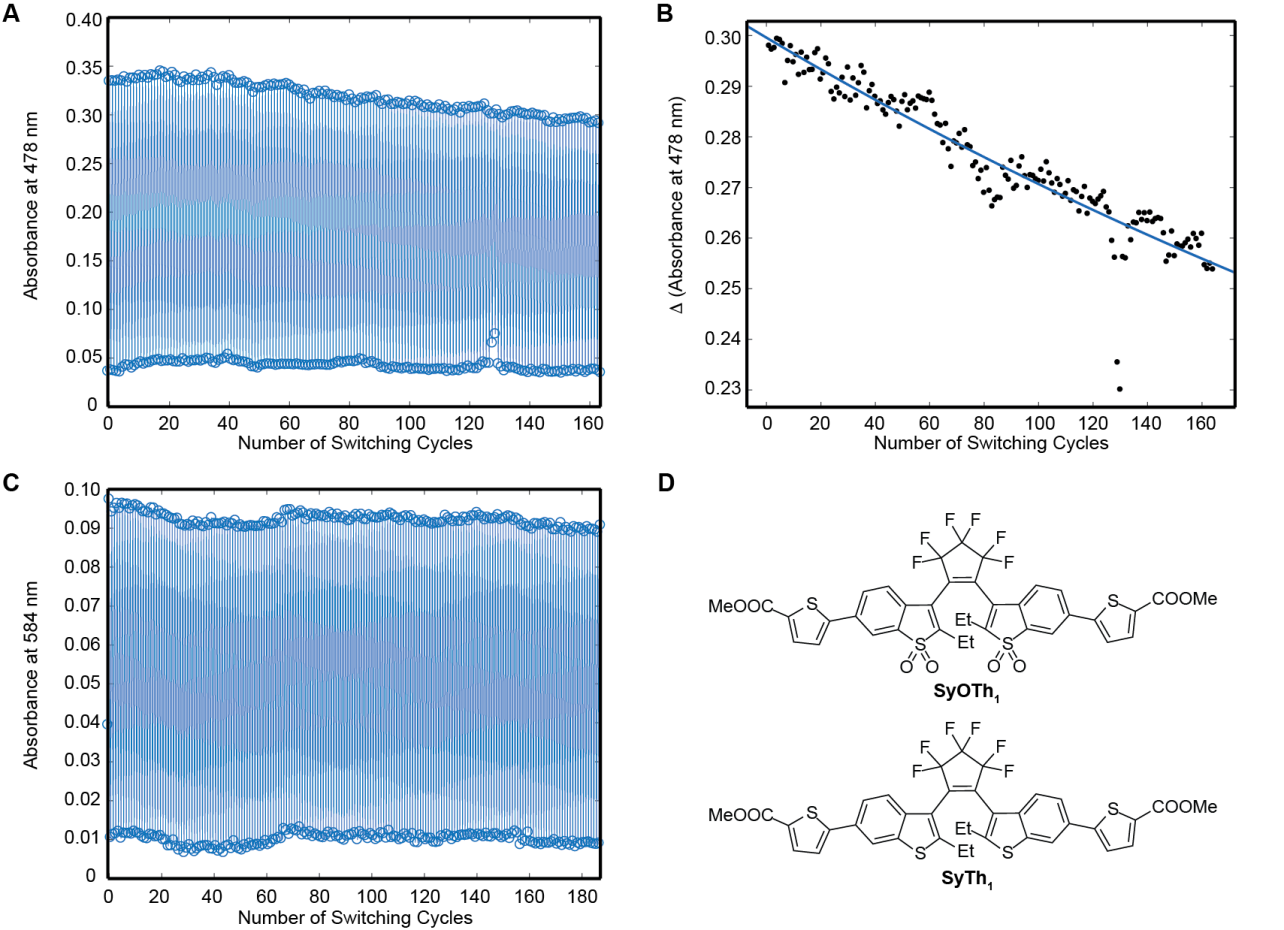
Fatigue resistances of compounds **SyOTh_1_** (A and B) and **SyTh_1_** (C). Parts A and C show the absorbance at certain wavelengths after cyclization (top values) and cycloreversion (bottom values). Part B shows the change in absorbance at 478 nm for each full switching cycle (CF–OF–CF) for compound **SyOTh_1_**. Dots: the measured values; blue line: an exponential fit. Part D shows the structures of both compounds.

Two processes – fluorescence and off-switching – compete for the depopulation of the same excited state [10]. Hence, the ratio of fluorescence quantum yield and off-switching quantum yield is proportional to the number of emitted photons (*N*_ph_) per cycle or per incursion into the fluorescent state (i.e., before off-switching takes place). Due to its high fluorescence quantum yield (φ_fl_) and low cycloreversion quantum yield (φ_CF→OF_), compound **SyOTh_1_** is expected to emit more than 5200 photons per cycle on average. Furthermore, compound **SyOTh_1_** proved to be highly fatigue resistant. As shown in Figure 5, it endured >160 switching cycles in acetonitrile solution while losing only ca. 15% of its absorption. The extrapolation of the change in absorption yielded about 340 switching cycles, until a 50% signal loss occurs. Thus, this compound is a good candidate for a marker applicable in STORM/PALM and MINFLUX super-resolution techniques [17–18]. It even showed superior switching kinetics (regarding the ratio of fluorescence quantum yield and cycloreversion quantum yield) compared to DAEs which were already applied in super-resolution microscopy [10,15]. The nonoxidized analog of **SyOTh_1_**, compound **SyTh_1_**, showed even higher fatigue resistance. In acetonitrile solution, extrapolation of the first 160 switching cycles predicted that >3500 switching cycles may be expected before a 50% signal loss will occur. The high fatigue resistance of compound **SyTh_1_** indicated that nonoxidized DAEs may, in general, be more photostable than oxidized DAEs. However, due to poor emission, **SyTh_1_** was not considered as a fluorescence marker. Another promising compound for applications in super-resolution microscopy was DAE **AsOTh_1_**. A high emission efficiency and a relatively fast cycloreversion, makes it a good candidate for a photoswitch applicable in RESOLFT microscopy.

### Urbach tail effect

**Figure 6:**
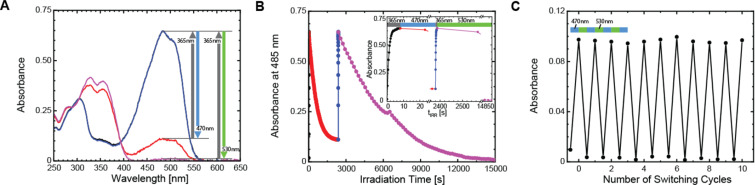
(A) Absorption spectra of compound **SyOTh_1_** in MeCN at the photostationary states under irradiation with 365 nm light (blue/black lines), 470 nm light (red line), and 530 nm light (purple line). Part B, irradiation kinetics (same color code). Part C, ten complete *on*/*off* switching cycles performed with visible light.

Under irradiation with visible light, most DAEs show complete conversion into the OF, because OFs do not absorb at wavelengths corresponding to the absorption band of the CF. However, compound **SyOTh_1_** presented a remarkably “low” value of α_CF→OF_ [470 nm] = 0.9 in MeCN. A similar behavior has been recently reported and ascribed to the so-called Urbach tail effect [19–20], i.e., the ring-closing photoreaction is induced by absorbing very weak hot bands of the OF, in the visible range. Neither nonoxidized DAEs, nor the asymmetric compound **AsOTh_1_** show this effect at observable levels; the other oxidized compounds are “irreversible”. To further investigate this phenomenon, **SyOTh_1_** was irradiated with visible light of even longer wavelength (530 nm). Remarkably, conversion from the CF to the OF was practically complete (Figure 6). Thus, this photochromic DAE can be switched “on” with 470 nm light to an extent of 10%, and switched “off” with 530 nm light. In Figure 6C we present ten switching cycles performed with visible light, alternating between blue and green irradiation (no UV light was used for switching!). The emission signal (not shown) presents the same on/off behavior.

## Conclusion

We prepared eight 1,2-bis[(2-ethylbenzo[*b*]thiophene)-3-yl]perfluorocyclopentenes with oxidized and nonoxidized cores and decorated them with mono- and bithiophene rings in symmetric and asymmetric fashions. By studying the optical properties of the new compounds, we found that DAEs with nonoxidized benzothiophene units possess fluorescent open forms with low emission efficiencies. The new DAEs are relatively fast switches, and, in particular, the nonoxidized DAEs exhibited high cycloreversion quantum yields. Due to a high on/off ratio of >270, good fatigue resistance and large fluorescence quantum yield, compound **SyOTh_1_** may be an excellent marker for single-molecule based super-resolution fluorescence microscopy, e.g., MINFLUX and STORM. In addition, **SyOTh_1_** presents a remarkable *on*-switching of 10% of its maximal fluorescence signal with 470 nm light, and the ring-closure reaction was fully reversible by irradiation with longer wavelengths. This offers the possibility of reversible switching a DAE only with visible, avoiding harmful (cytotoxic) UV irradiation. In view of the forthcoming solubilizing strategies applicable to fluorescent DAEs and intended for the crucial improvement of their fatigue resistance in aqueous solutions [21], the switching core of compound **AsOTh_1_** is expected to provide a promising marker for RESOLFT microscopy (after attaching the reactive group).

## Supporting Information

File 1 Experimental part and additional spectra of synthesized compounds.

## References

[1] Irie M, Fukaminato T, Matsuda K, Kobatake S (2014). Chem Rev.

[2] Jeong Y-C, Yang S I, Ahn K-H, Kim E (2005). Chem Commun.

[3] Jeong Y-C, Yang S I, Kim E, Ahn K-H (2006). Tetrahedron.

[4] Barrez E, Laurent G, Pavageau C, Sliwa M, Métivier R (2018). Phys Chem Chem Phys.

[5] Uno K, Bossi M L, Konen T, Belov V N, Irie M, Hell S W (2019). Adv Opt Mater.

[6] Åslund A, Herland A, Hammarström P, Nilsson K P R, Jonsson B-H, Inganäs O, Konradsson P (2007). Bioconjugate Chem.

[7] Becker R S, Seixas de Melo J, Maçanita A L, Elisei F (1996). J Phys Chem.

[8] Krayushkin M M, Bogacheva A M, Levchenko K S, Kobeleva O I, Valova T M, Barachevskii V A, Pozzo J-L, Struchkova M I, Shmelin P S, Kalik M A (2013). Mendeleev Commun.

[9] Uno K, Niikura H, Morimoto M, Ishibashi Y, Miyasaka H, Irie M (2011). J Am Chem Soc.

[10] Roubinet B, Weber M, Shojaei H, Bates M, Bossi M L, Belov V N, Irie M, Hell S W (2017). J Am Chem Soc.

[11] Chotana G A, Kallepalli V A, Maleczka R E, Smith M R (2008). Tetrahedron.

[12] Palamà I, Di Maria F, Viola I, Fabiano E, Gigli G, Bettini C, Barbarella G (2011). J Am Chem Soc.

[13] Klingstedt T, Shirani H, Mahler J, Wegenast-Braun B M, Nyström S, Goedert M, Jucker M, Nilsson K P R (2015). Chem – Eur J.

[14] Simon R A, Shirani H, Åslund K O A, Bäck M, Haroutunian V, Gandy S, Nilsson K P R (2014). Chem – Eur J.

[15] Roubinet B, Bossi M L, Alt P, Leutenegger M, Shojaei H, Schnorrenberg S, Nizamov S, Irie M, Belov V N, Hell S W (2016). Angew Chem, Int Ed.

[16] Yamaguchi T, Irie M (2006). J Photochem Photobiol, A.

[17] Henriques R, Griffiths C, Hesper Rego E, Mhlanga M M (2011). Biopolymers.

[18] Balzarotti F, Eilers Y, Gwosch K C, Gynnå A H, Westphal V, Stefani F D, Elf J, Hell S W (2017). Science.

[19] Kashihara R, Morimoto M, Ito S, Miyasaka H, Irie M (2017). J Am Chem Soc.

[20] Arai Y, Ito S, Fujita H, Yoneda Y, Kaji T, Takei S, Kashihara R, Morimoto M, Irie M, Miyasaka H (2017). Chem Commun.

[21] Uno K, Bossi M L, Irie M, Belov V N, Hell S W (2019). J Am Chem Soc.

