# Supplementary material for: Mono- and bithiophene-substituted diarylethene photoswitches with emissive open or closed forms
Source: Beilstein J Org Chem. 2019 Oct 1;15:2344–54. doi: 10.3762/bjoc.15.227 (PMC6808216; doi:10.3762/bjoc.15.227)
Supplement: File 1 — Experimental part and additional spectra of synthesized compounds. [file Beilstein_J_Org_Chem-15-2344-s001.pdf]

BEILSTEIN JOURNAL OF ORGANIC CHEMISTRY

Supporting Information

for

Mono- and bithiophene-substituted diarylethene photoswitches with emissive open or closed forms

A. Lennart Schleper, Mariano L. Bossi, Vladimir N. Belov and Stefan W. Hell

Beilstein J. Org. Chem. **2019,** *15,* 2344–2354. doi:10.3762/bjoc.15.227

Experimental part and additional spectra of synthesized compounds

License and Terms: This is a supporting information file under the terms of the Creative Commons Attribution License (http://creativecommons.org/licenses/by/4.0). Please note that the reuse, redistribution and reproduction in particular requires that the authors and source are credited.
The license is subject to the *Beilstein Journal of Organic Chemistry* terms and conditions: (https://www.beilstein-journals.org/bjoc)

Table of contents

Materials S2

Methods S3

Synthetic procedures S4

2-Ethylbenzo[*b*]thiophene (**1**) S4

3-Bromo-2-ethylbenzo[*b*]thiophene (**2**) S4

3,3'-(Perfluorocyclopent-1-ene-1,2-diyl)bis(2-ethylbenzo[*b*]thiophene) (**3**) S5

2-Ethyl-3-(2-(2-ethylbenzo[*b*]thiophen-3-yl)-3,3,4,4,5,5-hexafluorocyclopent-1-en-1-yl)-6-iodobenzo[b]thiophene (**4**) S5

3,3'-(Perfluorocyclopent-1-ene-1,2-diyl)bis(2-ethyl-6-iodobenzo[*b*]thiophene) (**5**) S6

3,3'-(Perfluorocyclopent-1-ene-1,2-diyl)bis(2-ethylbenzo[*b*]thiophene 1,1-dioxide) (**6**) S7

2-Ethyl-3-(2-(2-ethyl-1,1-dioxidobenzo[*b*]thiophen-3-yl)-3,3,4,4,5,5-hexafluorocyclopent-1-en-1-yl)-6-iodobenzo[*b*]thiophene 1,1-dioxide (**7**) S7

3,3'-(Perfluorocyclopent-1-ene-1,2-diyl)bis(2-ethyl-6-iodobenzo[*b*]thiophene 1,1-dioxide) (**8**) . S8

Methyl 5-(4,4,5,5-tetramethyl-1,3,2-dioxaborolan-2-yl)thiophene-2-carboxylate (**9**) S8

Methyl 2-(2-bromothiophen-3-yl)acetate (**10**) S9

Methyl 3'-(2-methoxy-2-oxoethyl)-[2,2'-bithiophene]-5-carboxylate (**11**) S9

Methyl 3'-(2-methoxy-2-oxoethyl)-5'-(4,4,5,5-tetramethyl-1,3,2-dioxaborolan-2-yl)-[2,2'-bithiophene]-5-carboxylate (**12**) S10

General procedure for the coupling of compounds **4**, **5**, **7**, and **8** with boronic acid esters **9** and **12** (GP) S10

AsTh$_1$ S10

SyTh$_1$ S11

AsOTh$_1$ S11

SyOTh$_1$ S12

AsTh$_2$ S12

SyTh$_2$ S13

AsOTh$_2$ S14

SyOTh$_2$ S14

SyTh$_2$-H S14

Additional images and spectra S16

NMR spectra, HPLC traces and photophysical data of new compounds S18

References S50

Materials

If not stated otherwise, the following chemicals were used without further purification: acetic acid (AcOH, 100%, EMSURE), acetonitrile (MeCN, for liquid chromatography/for analysis), benzo[*b*]thiophene (98%, Sigma Aldrich), bromoethane (98%, Fluka), *n*-butyl lithium solution (*n*-BuLi, 1.6 M in hexane, Sigma Aldrich), bis(1,5-cyclooctadiene)di-μ-methoxydiiridium(I) ([Ir(OMe)(COD)]2, 98%, STREM Chemicals), dichloromethane (DCM, for analysis), diethyl ether (Et_2O, for analysis), 1,4-dioxane (for analysis), 4,4'-di-*tert*-butyl-2,2'-dipyridyl ([*t*-Bu(bpy)]2, 98%, Sigma Aldrich), ethyl acetate (EA, for analysis), hexane (for analysis), hydrochloric acid (37%), aqueous hydrogen peroxide solution (H_2O_2, 50%), Sigma Aldrich), iodine (99.5%), iodine monochloride (ICl, ACS grade, Alfa Aesar), methanol (MeOH, for liquid chromatography), methyl thiophene-2-carboxylate (97%, abcr), *N*-bromosuccinimde (NBS, 99%, Sigma Aldrich), octafluorocyclopentene (98%, TCI), PEPPSI™-IPr (98%, Sigma Aldrich), periodic acid (99.5%, Fluka), potassium carbonate (K_2CO_3, for analysis), silica gel (SiO_2, 0.04-0.063 mm, Macherey & Nagel), sodium carbonate (Na_2CO_3, anhydrous), sodium chloride (NaCl, for analysis), sodium hydroxide solution (NaOH, 1 mol/L), sodium sulfate (Na_2SO_4, for analysis), sodium thiosulfate ($Na_2S_2O_3$, pentahydrate, for analysis), sulfuric acid (95–97%), tetrahydrofuran (THF, analysis grade), 4,4,5,5-tetramethyl-1,3,2-dioxaboralane (HB(pin), TCI, 97%), 3-thiopheneacetic acid (98%, Alfa Aesar), tricyclohexylphosphine solution ($P(Cy)_3$, 18% in toluene, Sigma Aldrich), trifluoroacetic acid (TFA, Sigma Aldrich), and tris(dibenzylideneacetone)dipalladium(0) ($Pd_2(dba)_3$, Pd 21.5%, Alfa Aesar). Anhydrous solvents were obtained by dynamic and static procedures with molecular sieves (3 Å, AppliChem). The residual water content was measured by Karl Fischer titration. Deuterated solvents were purchased from Deutero GmbH ($CDCl_3$ and CD_2Cl_2) and MERCK (DMF-d_7). Normal phase (SiO_2, Alugram Sil G/UV 254, Macherey & Nagel) and reverse phase (RP, TLC silica gel 60 RP-18 F254s, MERCK) TLC plates were purchased from the indicated distributors.

Methods

The NMR spectra were recorded on an Agilent 400-MR DD2 spectrometer. The chemical shifts (δ, ppm) were referenced to TMS by using the following chemical shifts of the residual solvent protons: $CDCl_3$ = 7.26; CD_2Cl_2 = 5.32; DMF-d_7 = 8.03 (s), 2.92 (quint), 2.75 (quint). Low resolution mass spectra (EI/ESI) were recorded using a Varian 500-MS system for ESI and a Thermo Scientific Focus GC gas chromatograph with a Thermo Scientific DSQ II analyzer for EI. High resolution mass spectra were recorded using a maXis and a micrOTOF mass spectrometer from Bruker Daltonics. Analytical high performance liquid chromatography (analytical HPLC) was performed using an UltiMate 3000 system from Thermo Fisher Scientific. Preparative high performance liquid chromatography (preparative HPLC, reverse phase) was carried out on a puriFlash 4250 system from Interchim. The following columns and solvent systems were used. System A: Interchim US10C18HQ-250/46 column (250 × 4.6 mm) with 0.1% aqueous TFA and MeCN (linear gradient from 30:70 → 0:100 within 30 min) at a flow rate of 1.2 mL/min. System B: Knauer Eurospher II 100-5 C18 250 × 16 mm column with 0.1% aqueous TFA and MeCN (linear gradient from 80:20 → 0:100 within 30 min, followed by 0:100) at a flow rate of 16 mL/min. System C: Knauer Eurospher II 100-5 C18 250 × 30 mm column with 0.1% aqueous TFA and MeCN (linear gradient from 20:80 → 0:100 within 30 min, followed by 0:100) at a flow rate of 45 mL/min. System D: Interchim US10C18HQ-250/212 column (250 × 21.2 mm) with 0.1% aqueous TFA and MeCN (linear gradient from 25:75 → 0:100 within 20 min, followed by 0:100) at a flow rate of 20 mL/min. UV–vis absorption and emission measurements where performed using a Cary 5000 UV-Vis-NIR spectrophotometer, a Cary Eclipse fluorescence spectrophotometer (both from Agilent Technologies), and a FluoTime 3000 fluorescence lifetime spectrometer (from Pico Quant). Irradiation experiments were performed in a home-made setup, consisting of LEDs as irradiation sources (Thorlabs, models M365L2 -365 nm/7.5 nm FWHM-, M470L3 -470 nm/25 nm FWHM-, and M530L3 -530 nm/33 nm FWHM-), a CCD array spectrometer (Ocean Optics, Flame Series), and a portable data acquisition device (myDAQ, National Instruments). The systems allowed for monitoring absorption and emission spectra before and after defined irradiation time intervals, with light of an appropriated wavelength. Samples were contained in a 3.5 mL quartz cuvette (Hellma Analytics), and were continuously stirred during irradiation and all measurements. The photon flux was measured using

a chemical actinometer (azobenzene in methanol [1] or Aberchrome 670 in toluene [2]), in identical geometry as the samples. Fluorescence spectra of DAEs in chloroform were recorded on a LS-55 fluorescence spectrometer from Perkin Elmer. Fluorescence quantum yields were measured using the following reference standards [3]: Fluorescein in 0.1 M NaOH (φ_{Fl} = 0.95), rhodamine 101 in methanol (φ_{Fl} = 1.00), and 9,10-diphenylanthracene in cyclohexane (φ_{Fl} = 0.90), were used for the corresponding emission range. All DAEs samples were diluted in acetonitrile to a concentration of 2–20 μM.

Synthetic procedures

2-Ethylbenzo[*b*]thiophene (1)

Under argon atmosphere, *n*-BuLi (1.6 m in hexane, 83.8 mL, 134 mmol) was added dropwise to a cooled (−78 °C) solution of benzo[*b*]thiophene (15.0 g, 112 mmol) in THF (180 mL), and the resulting emulsion was stirred for 4.5 h. Then, bromoethane (20.0 mL, 134 mmol) was added dropwise, and the reaction mixture was stirred for 18 h whilst slowly warming to room temperature. The reaction was quenched by the addition of water (50 mL), and the aqueous phase was extracted with Et_2O (3 × 50 mL). The combined organic solutions were dried over Na_2SO_4, and the solvent was evaporated under reduced pressure to yield 1 (15.6 g, 96.2 mmol, 86%) as off-white oil. The crude product was used without further purification. TLC (SiO_2, hexane): R_f = 0.33. ^{1}H NMR (400 MHz, CD_2Cl_2): δ [ppm] = 7.76 (d, $^3J_{H,H}$ = 7.6 Hz, 1 H), 7.67 (d, $^3J_{H,H}$ = 7.6 Hz, 1 H), 7.30 (ddd, $^3J_{H,H}$ = 7.6 Hz, $^3J_{H,H}$ = 7.6 Hz, $^4J_{H,H}$ = 1.3 Hz, 1 H), 7.24 (ddd, $^3J_{H,H}$ = 7.6 Hz, $^3J_{H,H}$ = 7.6 Hz, $^4J_{H,H}$ = 1.3 Hz, 1 H), 7.03 (d, $^4J_{H,H}$ = 1.2 Hz, 1 H), 2.93 (qd, $^3J_{H,H}$ = 7.6 Hz, $^4J_{H,H}$ = 1.2 Hz, 2 H), 1.37 (t, $^3J_{H,H}$ = 7.6 Hz, 3 H); data in accordance with the literature [4]. $C_{10}H_{10}S$. EI-MS: *m/z* (rel. int., %) = 162.12 (61) [$M^{+\bullet}$], 147.07 (100) [$M^{+\bullet}$–CH_3].

3-Bromo-2-ethylbenzo[*b*]thiophene (2)

Under argon atmosphere, *N*-bromosuccinimide (6.55 g, 36.8 mmol) was added to a −5 °C solution of **1** (5.53 g, 34.1 mmol) in THF (62 mL), and the solution was stirred for 14 hours. The reaction was quenched by addition of Na_2CO_3 solution (saturated, 20 mL) and $Na_2S_2O_3$ solution (saturated, 20 mL), and stirred for 30 minutes. The aqueous phase was separated and extracted with Et_2O (3 × 40 mL). After

evaporation of the solvent, the crude product was dissolved in hexane and filtered over SiO_2 (ca. 10 g) to give **2** as off-white oil (7.62 g, 31.6 mmol, 93%). TLC (SiO_2, hexane): R_f = 0.43. ^{1}H NMR (400 MHz, $CDCl_3$): δ [ppm] = 7.76-7.72 (m, 2 H), 7.41 (ddd, $^3J_{H,H}$ = 7.6 Hz, $^3J_{H,H}$ = 7.6 Hz, $^4J_{H,H}$ = 1.1 Hz, 1 H), 7.32 (ddd, $^3J_{H,H}$ = 7.6 Hz, $^3J_{H,H}$ = 7.6 Hz, $^4J_{H,H}$ = 1.1 Hz, 1 H), 2.97 (q, $^3J_{H,H}$ = 7.5 Hz, 2 H), 1.35 (t, $^3J_{H,H}$ = 7.5 Hz, 3 H); data in accordance with the literature [5]. $C_{10}H_9BrS$. EI-MS: *m/z* (rel. int., %) = 242.01 (61) [$M^{+\bullet}$, ^{81}Br], 239.96 (65) [$M^{+\bullet}$, ^{79}Br], 226.97 (100) [$M^{+\bullet}$–CH_3, ^{81}Br], 224.96 (96) [$M^{+\bullet}$–CH_3, ^{79}Br], 161.08 (46) [$M^{+\bullet}$–Br].

3,3'-(Perfluorocyclopent-1-ene-1,2-diyl)bis(2-ethylbenzo[*b*]thiophene) (3)

Under argon atmosphere, *n*-BuLi (1.6 M in hexane, 13 mL, 20.8 mmol) was added dropwise to a cooled (−78 °C) solution of **2** (4.39 g, 18.2 mmol) in dry Et_2O (160 mL). After stirring for 4 h, perfluorocyclopentene (1.22 mL, 9.10 mmol) was added slowly (in the course of one hour). The solution was slowly warmed to room temperature, and after stirring for 3 h, hydrochloric acid (1 m, 100 mL) was added. The aqueous phase was extracted with Et_2O (3 × 50 mL), the combined organic solutions dried over Na_2SO_4, the solvent evaporated under reduced pressure, and the crude product purified by column chromatography (ca. 200 g SiO_2, hexane). **3** (2.30 g, 4.63 mmol, 51%) was obtained as colorless crystals. TLC (SiO_2, hexane): R_f = 0.17. ^{1}H NMR (400 MHz, $CDCl_3$): δ [ppm] = 7.73-7.67 (m, 2.8 H), 7.63-7.58 (m, 1.1 H), 7.39 (t, $^3J_{H,H}$ = 7.3 Hz, 1.4 H), 7.31 (t, $^3J_{H,H}$ = 7.3 Hz, 1.5 H), 7.20-7.17 (m, 1.1 H) 2.99-2.89 (m, 0.6 H), 2.81-2.68 (m, 2.0 H), 2.50-2.41 (m, 1.4 H), 1.30 (t, $^3J_{H,H}$ = 7.5 Hz, 4.2 H), 0.80 (t, $^3J_{H,H}$ = 7.5 Hz, 1.8 H); data in accordance with the literature [6]. ^{19}F NMR (376 MHz, $CDCl_3$): δ [-ppm] = 109.4-111.3 (m, 4 H), 132.7 (quint, $^3J_{F,F}$ = 5.2 Hz, 1.4 H), 132.7-132.8 (m, 0.6 H). $C_{25}H_{18}F_6S_2$. EI-MS: *m/z* (rel. int., %) = 496.14 (100) [$M^{+\bullet}$], 439.06 (64) [$M^{+\bullet}$–CH_3S], 419.07 (52) [$M^{+\bullet}$–C_6H_5].

2-Ethyl-3-(2-(2-ethylbenzo[*b*]thiophen-3-yl)-3,3,4,4,5,5-hexafluorocyclopent-1-en-1-yl)-6-iodobenzo[*b*]thiophene (4)

In the course of 4 h, a ground mixture of iodine (69.5 mg, 274 μmol) and periodic acid (31.2 mg, 137 μmol) was added in four equal portions to a heated (45 °C) solution of **3** (200 mg, 403 μmol) in acetic acid (7 mL), water (0.2 mL), and sulfuric acid (0.15 mL). The solution was stirred for 2 h at 80 °C, and then cooled to room temperature. Then, the reaction mixture was poured onto crushed ice (50 g), stirred

for one hour, and filtered. The precipitate was rinsed with water (30 mL), dissolved in DCM (30 mL), and saturated aqueous solutions of Na_2CO_3 (20 mL) and $Na_2S_2O_3$ (20 mL) were added. The emulsion was stirred for 30 minutes, the phases separated and the aqueous phase extracted with DCM (3 × 30 mL). The combined organic extracts were dried over Na_2SO_4 and after evaporation of the solvent, the crude product was purified by preparative HPLC (System C, eluted after 31 min) and lyophilized to give the monoiodide **4** (35 mg, 56 μmol, 14%) as colorless solid. TLC (SiO_2, hexane): R_f = 0.19. ^{1}H NMR (400 MHz, $CDCl_3$): δ [ppm] = 8.05 (s, 0.5 H), 7.95 (s, 0.3 H), 7.72 (d, $^3J_{H,H}$ = 7.6 Hz, 0.7 H), 7.67-7.64 (m, 1.6 H), 7.53-7.52 (m, 0.3 H), 7.46-7.36 (m, 1.6 H), 7.31 (t, $^3J_{H,H}$ = 7.6 Hz, 1.0 H), 7.22-7.20 (m, 0.6 H), 2.96-2.86 (m, 0.6 H), 2.76-2.66 (m, 2.0 H), 2.47-2.36 (m, 1.4 H), 1.29 (t, $^3J_{H,H}$ = 7.4 Hz, 2.1 H), 0.85 (t, $^3J_{H,H}$ = 7.4 Hz, 2.2 H), 0.78 (t, $^3J_{H,H}$ = 7.4 Hz, 2.1 H). ^{19}F NMR (376 MHz, $CDCl_3$): δ [-ppm] = 109.3-111.4 (m, 4 F), 132.7 (m, 1.4 F), 132.7-132.8 (m, 0.6 F). HR-MS (ESI, neg.), *m/z* = 620.9620 (found [M-H^+]), 620.9648 calculated.

3,3'-(Perfluorocyclopent-1-ene-1,2-diyl)bis(2-ethyl-6-iodobenzo[*b*]thiophene) (5)
In the course of 1.5 h, a ground mixture of iodine (253 mg, 997 μmol) and periodic acid (82.6 mg, 363 μmol) was added in ten portions to a heated (60 °C) solution of **3** (450 mg, 906 μmol) in acetic acid (12 mL), water (0.4 mL) and sulfuric acid (0.3 mL). The solution was stirred for 3 h at 70 °C, slowly cooled to room temperature and poured onto crushed ice (40 g). The suspension was filtered, the precipitate was dissolved in DCM, and the organic solution was washed with saturated aqueous Na_2CO_3 solution and $Na_2S_2O_3$ solution. The organic phase was dried over Na_2SO_4 and concentrated under reduced pressure to give an orange oil. Purification by column chromatography (ca. 60 g SiO_2, hexane) gave **5** (236 mg, 315 μmol, 35%) as colorless foam. TLC (SiO_2, hexane): R_f = 0.20. ^{1}H NMR (400 MHz, $CDCl_3$): δ [ppm] = 8.06 (d, $^4J_{H,H}$ = 1.5 Hz, 1.1 H), 7.99 (d, $^4J_{H,H}$ = 1.5 Hz, 0.4 H), 7.66 (dd, $^3J_{H,H}$ = 8.6 Hz, $^4J_{H,H}$ = 1.5 Hz, 1.5 H), 7.48 (dd, $^3J_{H,H}$ = 8.6 Hz, $^4J_{H,H}$ = 1.5 Hz, 0.6 H), 7.37 (d, $^3J_{H,H}$ = 8.6 Hz, 1.4 H), 7.23 (d, $^3J_{H,H}$ = 8.6 Hz, 0.6 H), 2.93-2.84 (m, 0.6 H), 2.75-2.62 (m, 2.2 H), 2.42-2.33 (m, 1.5 H), 1.28 (t, $^3J_{H,H}$ = 7.5 Hz, partially overlapped with grease signals), 0.84 (t, $^3J_{H,H}$ = 7.5 Hz, partially overlapped with grease signals). ^{19}F NMR (376 MHz, $CDCl_3$): δ [-ppm] = 109.2-111.6 (m, 3.8 F), 132.6 (quint, $^3J_{F,F}$ = 5.5 Hz, 1.5 F), 132.7-132.9 (m, 0.7 F). $C_{25}H_{16}F_6I_2S_2$. ESI-MS (pos.): *m/z* (rel. int., %) = 748.3 (100) [$M^{+\bullet}$], 622.3 (49) [M+H^+–I].

3,3'-(Perfluorocyclopent-1-ene-1,2-diyl)bis(2-ethylbenzo[*b*]thiophene 1,1-dioxide) (6)

Compound **3** (600 mg, 1.21 mmol) was dissolved in acetic acid (20 mL), and H_2O_2 solution (3.0 mL) was added. After stirring at 130 °C for 3 hours, the reaction mixture was cooled to room temperature, water (20 mL) was added under stirring, and the suspension was filtered. The precipitate was washed with water and dried in vacuo to give **6** (670 mg, 1.20 mmol, 99%) as colorless powder. TLC (SiO_2, hexane/EA 3:1): R_f = 0.15. ^{1}H NMR (400 MHz, $CDCl_3$): δ [ppm] = 7.76 (d, $^3J_{H,H}$ = 7.3 Hz, 1.1 H), 7.68 (d, $^3J_{H,H}$ = 7.3 Hz, 0.9 H), 7.64-7.55 (m, 2.6 H), 7.48-7.39 (m, 1.5 H), 7.20 (d, $^3J_{H,H}$ = 7.3 Hz, 1.1 H), 7.14 (d, $^3J_{H,H}$ = 7.3 Hz, 0.6 H), 2.71-2.50 (m, 3.0 H), 2.43-2.33 (m, 1.2 H), 1.40 (t, $^3J_{H,H}$ = 7.6 Hz, 2.4 H), 1.05 (t, $^3J_{H,H}$ = 7.6 Hz, 3.6 H). ^{19}F NMR (376 MHz, $CDCl_3$): δ [-ppm] = 109.9 (m, 3.3 F), 110.2 (m, 0.7 F), 132.0-132.1 (m, 0.3 F), 132.2 (quint, $^3J_{F,F}$ = 5.1 Hz, 1.3 F), 132.4 (m, 0.4 F). $C_{25}H_{18}F_6O_4S_2$. ESI-MS (pos.): *m/z* (rel. int., %) = 599.3 (100) [$M+K^+$], 583.3 (78) [$M+Na^+$].

2-Ethyl-3-(2-(2-ethyl-1,1-dioxidobenzo[*b*]thiophen-3-yl)-3,3,4,4,5,5-hexafluorocyclopent-1-en-1-yl)-6-iodobenzo[*b*]thiophene 1,1-dioxide (7)

To a cooled solution of **6** (500 mg, 892 μmol) in conc. sulfuric acid (7 mL), a ground mixture of iodine (154 mg, 607 μmol) and periodic acid (69 mg, 303 μmol) was added in one portion. The reaction mixture was stirred for one hour, poured onto crushed ice (50 g), and stirred for another hour. After filtration and rinsing with water (30 mL), the precipitate was dissolved in DCM (30 mL), and saturated aqueous solutions of Na_2CO_3 (20 mL) and $Na_2S_2O_3$ (20 mL) were added. The emulsion was stirred for 30 minutes. Then, the phases were separated and the aqueous phase extracted with DCM (3 × 40 mL). The combined organic extracts were dried over Na_2SO_4, and after evaporation of the solvent, a brown solid (550 mg) was obtained. The crude product was purified by column chromatography (ca. 40 g, SiO_2, hexane/EA 1:0 $\rightarrow$ 2:1) to give the monoiodide **7** (173 mg, 252 μmol, 28%) as yellow foam. Additionally, diiodide 8 (41.0 mg, 50.5 μmol, 6%) was isolated as yellow foam. TLC (SiO_2, hexane/EA 3:1): R_f = 0.29. ^{1}H NMR (400 MHz, $CDCl_3$): δ [ppm] = 8.05 (d, $^4J_{H,H}$ = 1.3 Hz, 0.4 H), 7.97-7.93 (m, 0.8 H), 7.78-7.70 (m, 1.1 H), 7.64-7.56 (m, 1.1 H), 7.52-7.44 (m, 0.7 H), 7.19 (d, $^3J_{H,H}$ = 7.5 Hz, 0.5 H), 7.08 (d, $^3J_{H,H}$ = 7.3 Hz, 0.3 H), 6.92 (d, $^3J_{H,H}$ = 8.3 Hz, 0.5 H), 6.86 (d, $^3J_{H,H}$ = 8.1 Hz, 0.3 H), 2.68-2.46 (m, 2.3 H), 2.42-2.29 (m, 1.0 H), 1.42-1.37 (m, 2.3 H), 1.09 (t, $^3J_{H,H}$ = 7.6 Hz, 1.9 H), 1.01

(t, $^3J_{H,H}$ = 7.3 Hz, 1.8 H). ^{19}F NMR (376 MHz, $CDCl_3$): δ [-ppm] = 109.9-110.2 (m, 4.1 F), 132.0-132.1 (m, 0.3 F), 132.1-132.2 (m, 1.2 F), 132.3-132.4 (m, 0.4 F). $C_{25}H_{17}F_6IO_4S_2$. HR-MS (ESI, pos.), *m/z* = 686.9577 (found [M+H$^+$]), 686.9590 calculated.

3,3'-(Perfluorocyclopent-1-ene-1,2-diyl)bis(2-ethyl-6-iodobenzo[*b*]thiophene 1,1-dioxide) (8)

To a cooled (0 °C) solution of **6** (650 mg, 1.16 mmol) in sulfuric acid (16 mL), a mixture of ground iodine (1.03 g, 4.06 mmol) and periodic acid (463 mg, 2.02 mmol) was added in one portion. After stirring for 3 hours at 0 °C, the solution was added to stirred ice (50 g), and the mixture was stirred for 1 hour. After filtration, the solid was dissolved in DCM (20 mL), and aq. NaOH (10%, 30 mL) and $Na_2S_2O_3$ solution (1 mol/L, 30 mL) were added. After separation of the phases, and extraction of the aqueous phase with DCM (3 × 40 mL), the organic phases was concentrated in vacuo, and the crude product was purified by column chromatography (ca. 50 g SiO_2, hexane/EA 2:1) to give **8** (586 mg, 721 μmol, 62%) as yellow foam. TLC (SiO_2, hexane/EA 3:1): R_f = 0.45. ^{1}H NMR (400 MHz, $CDCl_3$): δ [ppm] = 8.06 ($^4J_{H,H}$ = 1.4 Hz, 1.0 H), 8.01 ($^4J_{H,H}$ = 1.4 Hz, 1.7 H), 7.95 (dd, $^3J_{H,H}$ = 8.1 Hz, $^4J_{H,H}$ = 1.4 Hz, 1.1 H), 7.78 (dd, $^3J_{H,H}$ = 8.1 Hz, $^4J_{H,H}$ = 1.4 Hz, 0.8 H), 6.91 (d, $^3J_{H,H}$ = 8.1 Hz, 1.2 H), 6.80 (d, $^3J_{H,H}$ = 8.1 Hz, 0.8 H), 2.63-2.42 (m, 2.9 H), 2.37-2.27 (m, 1.2 H), 1.38 (t, $^3J_{H,H}$ = 7.6 Hz, 2.7 H), 1.06 (t, $^3J_{H,H}$ = 7.6 Hz, 3.7 H); data in accordance with the literature [7]. ^{19}F NMR (376 MHz, $CDCl_3$): δ [-ppm] = 109.9 (t, $^3J_{F,F}$ = 5.1 Hz, 2.5 F), 110.0 (t, $^3J_{F,F}$ = 5.1 Hz, 1.6 F), 132.1 (quint, $^3J_{F,F}$ = 5.1 Hz, 0.4 H), 132.2 (quint, $^3J_{F,F}$ = 5.1 Hz, 1.2 F), 132.3 (quint, $^3J_{F,F}$ = 5.1 Hz, 0.4 F). $C_{25}H_{16}F_6I_2O_4S_2$. ESI-MS (neg.): *m/z* (rel. int., %) = 811.9 (100) [M–H$^+$].

Methyl 5-(4,4,5,5-tetramethyl-1,3,2-dioxaborolan-2-yl)thiophene-2-carboxylate (9)

Under argon atmosphere, 4,4,5,5-tetramethyl-1,3,2-dioxaboralane (HB(pin), 1.01 mL, 6.96 mmol) was added to a degassed solution of methyl thiophene-2-carboxylate (738 μL, 6.33 mmol), 4,4'-di-*tert*-butyl-2,2'-bipyridine (8.5 mg, 31.7 μmol), and $[Ir(OMe)(COD)]_2$ (8.0 mg, 15.8 μmol) in THF (25 mL). The reaction mixture was heated to 70 °C and stirred for 16 h. Then, the solvent was evaporated under reduced pressure, and compound **9** (1.65 g, 6.15 mmol, 97%) was obtained as off-

white solid and used without further purification. TLC (SiO_2, DCM): R_f = 0.27; heavily smearing (probably due to decomposition on silica gel). ^{1}H NMR (400 MHz, $CDCl_3$): δ [ppm] = 7.81 (d, $^3J_{H,H}$ = 3.6 Hz,1 H), 7.55 (d, $^3J_{H,H}$ = 3.6 Hz, 1 H), 3.89 (s, 3 H), 1.35 (s, 12 H); data in accordance with the literature [8]. $C_{12}H_{17}BO_4S$. EI-MS: *m/z* (rel. int., %) = 268.11 (66) [$M^{+•}$], 253.09 (78) [$M^{+•}$–CH_3], 182.02 (100) [$M^{+•}$–$C_5H_{10}O$], 169.04 (77) [$M^{+•}$–$C_6H_{11}O$].

Methyl 2-(2-bromothiophen-3-yl)acetate (10)

A suspension of thiophene-3-yl acetic acid (2.22 g, 15.6 mmol) and *N*-bromosuccinimide (2.78 g, 15.6 mmol) in DCM (140 mL) was kept in an ultrasound bath for 30 minutes. The solvent was evaporated under reduced pressure, and methanol (140 mL) and conc. sulfuric acid (0.5 mL) were added. The solution was stirred at 70 °C for 16 hours. After cooling to room temperature, the solution was neutralized by the addition of saturated aqueous Na_2CO_3 (saturated), extracted with DCM (3 × 80 mL), and the combined organic solutions were dried over Na_2SO_4. After evaporation of the solvent, the crude product was purified by column chromatography (ca. 300 g SiO_2, hexane/EA 1:0 $\rightarrow$ 9:1) to give **10** (2.30 g, 15.6 mmol, 63%) as colorless oil. TLC (SiO_2, DCM): R_f = 0.59. ^{1}H NMR (400 MHz, $CDCl_3$): δ [ppm] = 7.24 (d, $^3J_{H,H}$ = 5.7 Hz, 1 H), 6.93 (d, $^3J_{H,H}$ = 5.7 Hz, 1 H), 3.72 (s, 3 H), 3.64 (s, 2 H); data in accordance with the literature [9]. $C_7H_7BrO_2S$. EI-MS: *m/z* (rel. int., %) = 235.98 (28) [$M^{+•}$, ^{81}Br], 233.97 (28) [$C_7H_7BrO_2S$, $M^{+•}$, ^{79}Br] 176.96 (100) [$M^{+•}$–$C_2H_3O_2$], 174.91 (100) [$M^{+•}$–$C_2H_3O_2$], 155.03 (66) [$M^{+•}$–Br].

Methyl 3'-(2-methoxy-2-oxoethyl)-[2,2'-bithiophene]-5-carboxylate (11)

Under argon atmosphere, PEPPSI-IPr (173 mg, 255 μmol) was added to a degassed suspension of **10** (1.20 g, 5.10 mmol), **9** (1.37 g, 5.10 mmol) and K_2CO_3 (2.12 g, 15.3 mmol) in dioxane/methanol (40 mL, 4:1). After stirring at 70 °C for 20 minutes, the solution was cooled to room temperature and the pH adjusted to 4 by the addition of 1 M hydrochloric acid. The solution was extracted with ethyl acetate (3 × 40 mL), and the combined organic extracts were washed with brine and dried over Na_2SO_4. The solvent was evaporated under reduced pressure, and the crude product was purified by column chromatography (ca. 300 g SiO_2, DCM) to give **11** (1.12 g, 3.78 mmol, 74%) as yellow oil. Within two days at room temperature, the oil solidified to give an off-white solid. M.p. = 75 °C. TLC (SiO_2, DCM): R_f = 0.25. ^{1}H NMR

(400 MHz, $CDCl_3$): δ [ppm] = 7.75 (d, $^3J_{H,H}$ = 3.9 Hz, 1 H), 7.30 (d, $^3J_{H,H}$ = 5.3 Hz, 1 H), 7.17 (d, $^3J_{H,H}$ = 3.9 Hz, 1 H), 7.06 (d, $^3J_{H,H}$ = 5.3 Hz, 1 H), 3.89 (s, 3 H), 3.79 (s, 2 H), 3.72 (s, 3 H); data in accordance with literature [10]. $C_{13}H_{12}O_4S_2$. EI-MS: *m/z* (rel. int., %) = 295.97 (100) [$M^{+\bullet}$], 236.95 (82) [$M^{+\bullet}$–$C_2H_3O_2$], 177.99 (90) [$M^{+\bullet}$–2x$C_2H_3O_2$].

Methyl 3'-(2-methoxy-2-oxoethyl)-5'-(4,4,5,5-tetramethyl-1,3,2-dioxaborolan-2-yl)-[2,2'-bithiophene]-5-carboxylate (12)

Under argon atmosphere, HB(pin) (237 μL, 1.64 mmol) was added to a solution of **11** (350 mg, 1.18 mmol), $[Ir(OMe)(COD)]_2$ (18.0 mg, 35.4 μmol), and 4,4'-di-*tert*-butyl-2,2'-bipyridine (19.0 mg, 70.9 μmol) in THF (3 mL), and the reaction mixture was stirred at 75 °C for 36 h. After evaporation of the solvent under reduced pressure, the crude product (**12**, 570 mg, 70% purity, 79% yield) was obtained as dark oil and used without further purification. TLC (SiO_2, DCM): R_f = 0.14; heavily smearing (probably due to decomposition on silica gel). ^1H NMR (400 MHz, $CDCl_3$): δ [ppm] = 7.75 (d, $^3J_{H,H}$ = 3.9 Hz, 1 H), 7.55 (s, 1 H), 7.21 (d, $^3J_{H,H}$ = 3.9 Hz, 1 H), 3.90 (s, 3 H), 3.79 (s, 2H), 3.72 (s, 3H), 1.34 (s, 12H); data in accordance with the literature [11]. $C_{19}H_{23}BO_6S_2$. EI-MS: *m/z* (rel. int., %) = 422.17 (100) [$M^{+\bullet}$], 363.15 (38) [$M^{+\bullet}$–$C_2H_3O_2$], 204.03 (49) [$M^{+\bullet}$–2x$C_2H_3O_2$–$C_6H_{12}O$].

General procedure for the coupling of compounds 4, 5, 7, and 8 with boronic acid esters 9 and 12 (GP)

According to literature protocols [7], a solution of tricyclohexylphosphine (0.36 equiv) in toluene (18%) was added to a degassed emulsion of compound **4**, **5**, **7**, or **8**, $Pd_2(dba)_3$ (0.18 equiv), the thiophenyl boronic acid pinacol ester **9** or **12** (1 equiv), THF, and saturated aqueous K_2CO_3 solution. After the reaction mixture was stirred at 60 °C for one hour, it was brought to pH 4 by the addition of 1 M hydrochloric acid. The phases were separated and the aqueous phase was extracted with DCM. The combined organic extracts were dried over Na_2SO_4 and after evaporation of the solvent, the crude product was purified by preparative HPLC and lyophilized.

AsTh$_1$

According to the GP, **4** (15 mg, 24 μmol), **9** (6.5 mg, 24 μmol), $Pd_2(dba)_3$ (4.0 mg, 4.3 μmol), $P(Cy)_3$ solution (23 μL, 8.7 μmol), sat. K_2CO_3 solution (2 mL), and THF

(2 mL) were stirred for one hour at 60 °C. The crude product (37 mg) was obtained as described in the GP as yellow oil. After preparative HPLC (system D, eluted after 27 min) and lyophilization, **AsTh$_1$** (4.0 mg, 6.3 μmol, 26%) was obtained as colorless solid. TLC (SiO_2, Hex/EA 1:1): R_f = 0.72.^{1}H NMR (400 MHz, $CDCl_3$): δ [ppm] = 7.97 (d, $^4J_{H,H}$ = 1.6 Hz, 0.5 H), 7.87 (d, $^4J_{H,H}$ = 1.6 Hz, 0.2 H), 7.78 (d, $^3J_{H,H}$ = 3.9 Hz, 0.6 H), 7.75-7.69 (m, 1.0 H), 7.70-7.61 (m, 2.1 H), 7.60-7.55 (m, 0.6 H), 7.50-7.36 (m, 1.0 H), 7.35-7.28 (m, 1.3 H), 7.25-2.17 (m, 1.2 H), 3.91 (s, 1.9 H), 3.89 (s, 0.9 H), 3.03-2.86 (m, 0.6 H), 2.80-2.62 (m, 2.0 H), 2.47-2.40 (m, 1.4 H), 1.33-1.28 (m, 2.3 H), 0.86 (t, $^3J_{H,H}$ = 7.5 Hz, 2.4 H), 0.80 (t, $^3J_{H,H}$ = 7.5 Hz, 2.1 H).^{19}F NMR (376 MHz, $CDCl_3$): δ [-ppm] = 109.3-111.4 (m, 4.0 F), 132.6-132.8 (2.0 F).$C_{31}H_{22}F_6O_2S_3$. HR-MS (ESI, pos.), *m/z* = 637.0754 (found [M+H$^+$]), 637.0759 calculated.

SyTh$_1$

According to the GP, **5** (35 mg, 47 μmol), **9** (25 mg, 94 μmol), $Pd_2(dba)_3$ (15 mg, 17 μmol), $P(Cy)_3$ solution (50 μL, 34 μmol), sat. K_2CO_3 solution (4 mL), and THF (4 mL) were stirred for one hour at 60 °C. The crude product (67 mg) was obtained as described in the GP as dark yellow oil. After preparative HPLC (system A, eluted after 39 min) and lyophilization, **SyTh$_1$** (7.7 mg, 9.9 μmol, 21%) was obtained as colorless solid. TLC (SiO_2, Hex/EA 1:1): R_f = 0.64. ^{1}H NMR (400 MHz, $CDCl_3$): δ [ppm] = 7.98 (s, 1.2 H), 7.89 (d, $^4J_{H,H}$ = 1.6 Hz, 0.5 H), 7.78 (d, $^3J_{H,H}$ = 3.9 Hz, 1.2 H), 7.73 (d, $^3J_{H,H}$ = 3.9 Hz, 0.6 H), 7.69-7.65 (m, 2.6 H), 7.58 (d, $^3J_{H,H}$ = 8.5 Hz, 0.6 H), 7.48 (dd, $^3J_{H,H}$ = 8.5 Hz, $^4J_{H,H}$ = 1.7 Hz, 0.6 H), 7.33 (d, $^3J_{H,H}$ = 3.9 Hz, 1.3 H), 7.25 (d, $^3J_{H,H}$ = 3.9 Hz, 0.7 H), 3.92 (s, 3.6 H), 3.89 (s, 1.7 H), 2.99-2.90 (m, 0.6 H), 2.81-2.69 (m, 1.9 H), 2.50-2.41 (m, 1.3 H), 1.32 (t, $^3J_{H,H}$ = 7.5 Hz, 2.6 H), 0.87 (t, $^3J_{H,H}$ = 7.5 Hz, 5.1 H, partially overlapped with grease signals). ^{19}F NMR (376 MHz, $CDCl_3$): δ [-ppm] = 109.3-111.4 (m, 3.9 F), 132.6 (quint, $^3J_{F,F}$ = 5.5 Hz, 1.5 F), 132.7-132.8 (m, 0.5 F). $C_{37}H_{26}F_6O_4S_4$. HR-MS (ESI, pos.), *m/z* = 777.0677 (found [M+H$^+$]), 777.0691 calculated.

AsOTh$_1$

According to the GP, **7** (32 mg, 47 μmol), **9** (13 mg, 47 μmol), $Pd_2(dba)_3$ (7.7 mg, 8.4 μmol), $P(Cy)_3$ solution (25 μL, 17 μmol), sat. K_2CO_3 solution (3 mL), and THF (3 mL) were stirred for one hour at 60 °C. The crude product (84 mg) was obtained as described in the GP as dark yellow oil. After preparative HPLC (system A, eluted

after 17 min) and lyophilization, **AsOTh1** (18 mg, 26 μmol, 56%) was obtained as yellow solid. Additionally, the closed form was isolated as orange solid (0.2 mg, 0.3 μmol, 1%, eluted after 22 min). TLC (SiO_2, Hex/EA 1:1): R_f = 0.54. ^{1}H NMR (400 MHz, $CDCl_3$): δ [ppm] = 7.98 (d, $^4J_{H,H}$ = 1.6 Hz, 0.5 H), 7.91 (d, $^4J_{H,H}$ = 1.6 Hz, 0.3 H), 7.84-7.76 (m, 2.1 H), 7.71-7.68 (m, 0.4 H), 7.65-7.56 (m, 1.5 H), 7.48-7.45 (m, 0.7 H), 7.39 (d, $^3J_{H,H}$ = 4.0 Hz, 0.6 H), 7.32 (d, $^4J_{H,H}$ = 4.0 Hz, 0.4 H), 7.24-7.20 (m, 1.1 H), 7.18-7.13 (m, 0.7 H), 3.93-3.91 (m, 2.9 H), 2.69-2.52 (m, 2.6 H), 2.44-2.35 (m, 1.2 H), 1.41 (t, $^3J_{H,H}$ = 7.6 Hz, 3.0 H), 1.10 (t, $^3J_{H,H}$ = 7.6 Hz, 2.1 H), 1.04 (t, $^3J_{H,H}$ = 7.6 Hz, 2.0 H). ^{19}F NMR (376 MHz, $CDCl_3$): δ [-ppm] = 109.8-110.2 (m, 3.9 F), 132.0-132.1 (m, 0.4 F), 132.2 (quint, 1.3 F), 132.3-132.4 (m, 0.3 F). $C_{31}H_{22}F_6O_6S_3$. HR-MS (ESI, pos.), *m/z* = 718.0817 (found [M+NH_4^+]), 718.0821 calculated.

SyOTh1

According to the GP, **8** (30 mg, 37 μmol), **9** (20 mg, 74 μmol), $Pd_2(dba)_3$ (6.1 mg, 6.7 μmol), $P(Cy)_3$ solution (20 μL, 13 μmol), sat. K_2CO_3 solution (2 mL), and THF (2 mL) were stirred for one hour at 60 °C. The crude product (24 mg) was obtained as described in the GP, followed by column chromatography (ca. 30 g SiO_2, DCM) as red foam. After preparative HPLC (system A, eluted after 22 min) and lyophilization, **SyOTh1** (20 mg, 27.8 μmol, 75%) was obtained as orange solid. Additionally, the closed form was isolated as red solid (0.3 mg, 0.4 μmol, 1%, eluted after 28 min). TLC (SiO_2, Hex/EA 1:1): R_f = 0.56. ^{1}H NMR (400 MHz, $CDCl_3$): δ [ppm] = 7.99 (d, $^4J_{H,H}$ = 1.5 Hz, 1.2 H), 7.93 (d, $^4J_{H,H}$ = 1.4 Hz, 0.8 H), 7.83 (dd, $^3J_{H,H}$ = 8.0 Hz, $^4J_{H,H}$ = 1.6 Hz, 1.4 H), 7.80 (d, $^3J_{H,H}$ = 3.9 Hz, 1.0 H), 7.75 (d, $^3J_{H,H}$ = 3.9 Hz, 0.7 H), 7.66 (dd, $^3J_{H,H}$ = 8.1 Hz, $^4J_{H,H}$ = 1.6 Hz, 0.7 H), 7.40 (d, $^3J_{H,H}$ = 3.9 Hz, 1.3 H), 7.33 (d, $^3J_{H,H}$ = 3.9 Hz, 0.9 H), 7.24 (d, $^3J_{H,H}$ = 8.1 Hz, 0.9 H), 7.17 (d, $^3J_{H,H}$ = 8.1 Hz, 0.6 H), 3.93 (s, 3.3 H), 3.90 (s, 2.1 H), 2.69-2.51 (m, 3.0 H), 2.44-2.36 (m, 1.2 H), 1.42 (t, $^3J_{H,H}$ = 7.5 Hz, 2.9 H), 1.10 (d, $^3J_{H,H}$ = 7.5 Hz, 4.0 H). ^{19}F NMR (376 MHz, $CDCl_3$): δ [-ppm] =109.8-110.0 (m, 4.1 F), 132.0-132.1 (m, 0.4 F), 132.1-132.2 (m, 1.2 F), 132.3 (m, 0.3 F). $C_{37}H_{26}F_6O_8S_4$. HR-MS (ESI, pos.), *m/z* = 858.0742 (found [M+NH_4^+]), 858.0753 calculated.

AsTh2

According to the GP, **4** (15 mg, 24 μmol), **12** (10 mg, 24 μmol), $Pd_2(dba)_3$ (4.0 mg, 4.3 μmol), $P(Cy)_3$ solution (13 μL, 8.7 μmol), sat. K_2CO_3 solution (2 mL), and THF

(2 mL) were stirred for one hour at 60 °C. The crude product (37 mg) was obtained as described in the GP as orange oil. After preparative HPLC (system D, eluted after 30 min) and lyophilization, **AsTh₂** (8.0 mg, 10 μmol, 42%) was obtained as yellow solid. TLC (SiO_2, Hex/EA 1:1): R_f = 0.64. ^{1}H NMR (400 MHz, $CDCl_3$): δ [ppm] = 7.93 (d, $^4J_{H,H}$ = 1.6 Hz, 0.6 H), 7.82 (d, $^4J_{H,H}$ = 1.6 Hz, 0.2 H), 7.78-7.75 (m, 0.9 H) 7.72 (d, $^3J_{H,H}$ = 7.8 Hz, 0.9 H), 7.67 (d, $^3J_{H,H}$ = 8.1 Hz, 1.5 H), 7.61 (dd, $^3J_{H,H}$ = 8.5 Hz, $^4J_{H,H}$ = 1.6 Hz, 0.9 H), 7.58-7.54 (m, 0.6 H), 7.42-7.36 (m, 1.1 H), 7.34-7.28 (m, 1.4 H), 7.24-7.16 (m, 1.8 H), 3.91 (s, 1.9 H), 3.90 (s, 0.9 H), 3.82 (s, 1.2 H), 3.79 (s, 0.5 H), 3.76 (s, 1.8 H), 3.74 (s, 0.8 H), 2.96-2.89 (m, 0.6 H), 2.78-2.67 (m, 2.1 H), 2.47-2.39 (m, 1.4 H), 1.32-1.28 (m, 2.1 H), 0.84 (t, $^3J_{H,H}$ = 7.5 Hz, 2.5 H), 0.79 (t, $^3J_{H,H}$ = 7.5 Hz, 2.1 H). ^{19}F NMR (376 MHz, $CDCl_3$): δ [-ppm] = 109.3-111.4 (m, 4.0 F), 132.6- 132.8 (m, 2.0 F). $C_{38}H_{28}F_6O_4S_4$. HR-MS (ESI, pos.), *m/z* = 791.0836 (found [M+H$^+$]), 791.0847 calculated.

SyTh₂

According to the GP, **5** (35 mg, 47 μmol), **12** (40 mg, 94 μmol), $Pd_2(dba)_3$ (15 mg, 17 μmol), $P(Cy)_3$ solution (50 μL, 34 μmol), sat. K_2CO_3 solution (4 mL), and THF (4 mL) were stirred for one hour at 60 °C. The crude product (101 mg) was obtained as described in the GP as dark yellow oil. After preparative HPLC (system A, eluted after 45 min) and lyophilization, **SyTh₂** (21 mg, 19 μmol, 41%) was obtained as light green solid. TLC (SiO_2, Hex/EA 1:1): R_f = 0.58. ^{1}H NMR (400 MHz, $CDCl_3$): δ [ppm] = 7.94 (d, $^4J_{H,H}$ = 1.6 Hz, 1.3 H), 7.84 (d, $^4J_{H,H}$ = 1.6 Hz, 0.6 H), 7.77 (d, $^3J_{H,H}$ = 3.9 Hz, 1.3 H), 7.74 (d, $^3J_{H,H}$ = 3.9 Hz, 0.6 H), 7.67 (d, $^3J_{H,H}$ = 8.3 Hz, 1.4 H), 7.62 (dd, $^3J_{H,H}$ = 8.5 Hz, $^4J_{H,H}$ = 1.6 Hz, 1.3 H), 7.55 (d, $^3J_{H,H}$ = 8.4 Hz, 0.6 H), 7.44 (dd, $^3J_{H,H}$ = 8.5 Hz, $^4J_{H,H}$ = 1.6 Hz, 0.6 H), 7.33 (s, 1.3 H), 7.25 (s, 0.5 H), 7.22 (d, $^3J_{H,H}$ = 3.9 Hz, 1.2 H), 7.18 (d, $^3J_{H,H}$ = 3.9 Hz, 0.5 H), 3.91 (s, 3.7 H), 3.90 (s, 1.6 H), 3.82 (s, 2.5 H), 3.78 (s, 1.3 H), 3.76 (s, 3.6 H), 3.72 (s, 1.6 H), 2.95-2.91 (m, 0.7 H), 2.79-2.70 (m, 2.1 H), 2.48-2.42 (m, 1.5 H), 1.31 (d, $^3J_{H,H}$ = 7.5 Hz, 3.0 H), 0.85 (d, $^3J_{H,H}$ = 7.5 Hz, 5.3 H, partially overlapped with grease signals). ^{19}F NMR (376 MHz, $CDCl_3$): δ [-ppm] = 109.2-111.4 (m, 3.9 F), 132.6-132.8 (m, 2.1 F). $C_{51}H_{38}F_6O_8S_6$. HR-MS (ESI, pos.), *m/z* = 1102.1125 (found [M+NH$_4^+$]), 1102.1133 calculated.

AsOTh$_2$

According to the GP, **7** (46 mg, 66 μmol), **12** (28 mg, 66 μmol), $Pd_2(dba)_3$ (11 mg, 12 μmol), $P(Cy)_3$ solution (36 μL, 24 μmol), sat. K_2CO_3 solution (3 mL), and THF (3 mL) were stirred for one hour at 60 °C. The crude product (110 mg) was obtained as described in the GP as orange oil. After preparative HPLC (system A, eluted after 22 min) and lyophilization, **AsOTh$_2$** (20 mg, 24 μmol, 36%) was obtained as orange solid. TLC (SiO_2, Hex/EA 1:1): R_f = 0.47. ^{1}H NMR (400 MHz, $CDCl_3$): δ [ppm] = 7.95 (d, $^4J_{H,H}$ = 1.6 Hz, 0.6 H), 7.87 (d, $^4J_{H,H}$ = 1.6 Hz, 0.4 H), 7.79-7.76 (m, 2.1 H), 7.71-7.69 (m, 0.4 H), 7.64-7.56 (m, 1.6 H), 7.50-7.44 (m, 0.8 H), 7.41 (s, 0.5 H), 7.34 (s, 0.4 H), 7.25-1.19 (m, 2.0 H), 7.15-7.12 (m, 0.7 H), 3.92 (s, 3.0 H), 3.82-3.46 (m, 4.6 H), 2.70-2.50 (m, 2.8 H), 2.43-2.35 (m, 1.2 H), 1.43-1.39 (m, 2.7 H), 1.09 (t, $^3J_{H,H}$ = 7.5 Hz, 2.2 H), 1.04 (t, $^3J_{H,H}$ = 7.5 Hz, 2.0 H). ^{19}F NMR (376 MHz, $CDCl_3$): δ [-ppm] = 109.9-110.2 (m, 4.1 F), 132.0-132.1 (m, 0.4 F), 132.2 (m, 1.2 F), 132.3-132.3 (m, 0.3 F). $C_{38}H_{28}F_6O_8S_4$. HR-MS (ESI, pos.), *m/z* = 872.0907 (found [M+NH_4^+]), 872.0909 calculated.

SyOTh$_2$

According to the GP, **8** (45 mg, 55 μmol), **12** (47 mg, 110 μmol), $Pd_2(dba)_3$ (9.0 mg, 10 μmol), $P(Cy)_3$ solution (30 μL, 20 μmol), sat. K_2CO_3 solution (3 mL), and THF (3 mL) were stirred for one hour at 60 °C. The crude product (29 mg) was obtained as described in the GP, followed by column chromatography (ca. 30 g SiO_2, DCM) as red foam. After preparative HPLC (System A, eluted after 28 min) and lyophilization, **SyOTh$_2$** (18 mg, 16 μmol 29%) was obtained as orange solid. TLC (SiO_2, Hex/EA 1:1): R_f = 0.43. ^{1}H NMR (400 MHz, $CDCl_3$): δ [ppm] = 7.96 (s, 1.1 H), 7.89 (s, 0.8 H), 7.79-7.75 (m, 2.9 H), 7.61 (d, $^3J_{H,H}$ = 7.1 Hz, 0.7 H), 7.41 (s, 1.1 H), 7.35 (s, 0.8 H), 7.25-1.19 (m, 2.9 H), 7.13 (d, $^3J_{H,H}$ = 7.9 Hz, 0.7 H), 3.92-3.90 (m, 5.9 H), 3.82-3.73 (m, 9.5 H), 2.68-2.53 (m, 2.7 H), 2.43-2.38 (m, 1.2 H), 1.42 (d, $^3J_{H,H}$ = 7.5 Hz, 3.4 H), 1.09 (d, $^3J_{H,H}$ = 7.5 Hz, 4.5 H). ^{19}F NMR (376 MHz, $CDCl_3$): δ [-ppm] = 109.8-110.0 (m, 4.0 F), 132.1-132.3 (m, 2.0 F). $C_{51}H_{38}F_6O_{12}S_6$. HR-MS (ESI, pos.), *m/z* = 1166.0925 (found [M+NH_4^+]), 1166.0930 calculated.

SyTh$_2$-H

Aqueous sodium hydroxide solution (1 M, 17 μL, 17 μmol) was added to a solution of **SyTh$_2$** (3.0 mg, 2.8 μmol) in dioxane (0.3 mL) and the emulsion was stirred for 16 h

at 60 °C. Afterwards, the emulsion was diluted with water and acetonitrile and the solution was lyophilized. The crude product was purified by preparative HPLC (system B, eluted after 31 min) and after lyophilization, **SyTh$_2$-H** (2.4 mg, 2.3 μmol, 84%) was obtained as light green solid. TLC (reverse phase, MeCN/H_2O/TFA 100:10:1): R_f = 0.35. ^{1}H NMR (400 MHz, $CDCl_3$): δ [ppm] = 8.42 (d, $^4J_{H,H}$ = 1.4 Hz, 1.2 H), 8.30 (d, $^4J_{H,H}$ = 1.4 Hz, 0.7 H), 7.93 (d, $^4J_{H,H}$ = 1.7 Hz, 0.4 H), 7.91 (d, $^4J_{H,H}$ = 1.7 Hz, 0.6 H), 7.89 (s, 0.3 H), 7.87-7.85 (m, 1.0 H), 7.84-7.81 (m, 1.3 H), 7.79 (d, $^3J_{H,H}$ = 3.9 Hz, 0.7 H), 7.74 (d, $^4J_{H,H}$ = 1.7 Hz, 0.3 H), 7.72 (s, 1.2 H), 7.60 (s, 0.6 H), 7.44 (d, $^3J_{H,H}$ = 3.9 Hz, 1.0 H), 7.39 (d, $^3J_{H,H}$ = 3.9 Hz, 0.6 H), 3.93 (s, 2.1 H), 3.87 (s, 1.2 H), 3.09-2.64 (m, overlapped with solvent signals), 1.35 (t, $^3J_{H,H}$ = 7.5 Hz, 2.7 H), 0.92 (t, $^3J_{H,H}$ = 7.5 Hz, 3.9 H). ^{19}F NMR (376 MHz, $CDCl_3$): δ [-ppm] = 109.2-111.5 (m, 4.1 F), 132.2-132.3 (m, 0.4 F), 132.3-132.4 (m, 1.2 F), 132.4-132.5 (m, 0.3 F). $C_{47}H_{30}F_6O_8S_6$. HR-MS (ESI, neg.), *m/z* = 1027.0085 (found [M–H$^+$]), 1027.0096 calculated.

Additional images and spectra

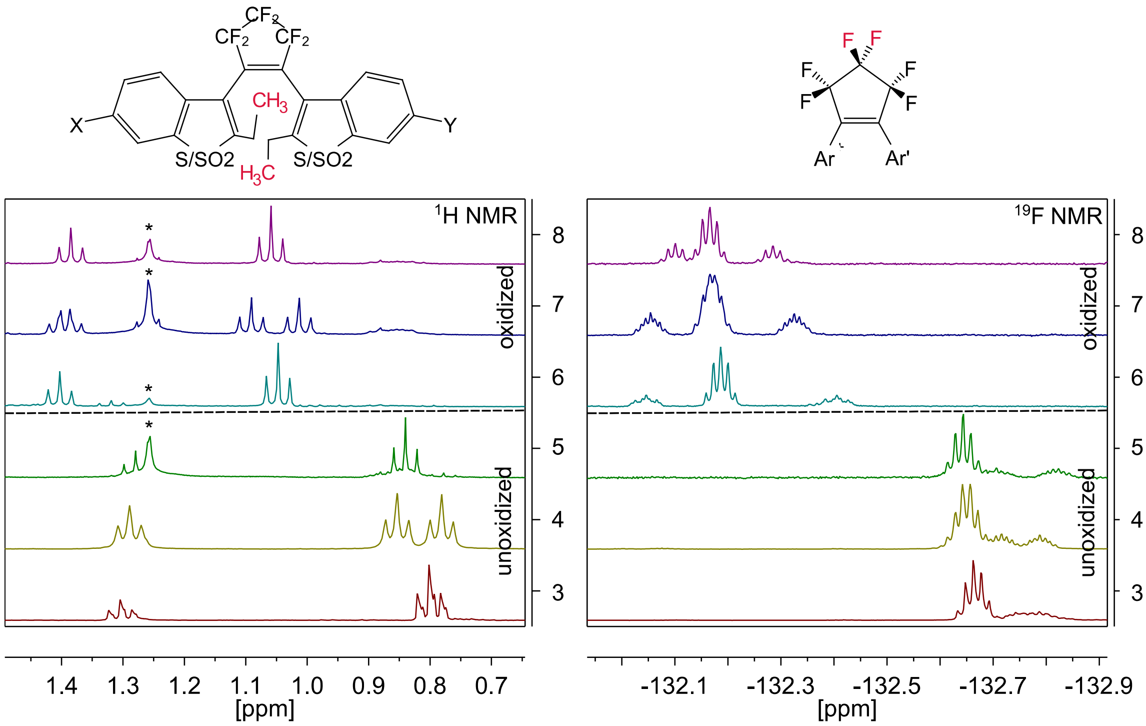

Figure S1: Sections of ^{1}H NMR and ^{19}F NMR spectra of compounds **3**–**8** (for structures, see main text), showing signals of methyl groups (left) and fluorine atoms (right). Compounds **3**, **5**, **6** and **8** are symmetric, compounds **4** and **7** asymmetric. In the left panel, the presence of more than two triplets for compounds **4** and **7** is conform with monoiodination (X = H, Y = I); *parallel*- and *antiparallel* conformers are present. In the right panel, the "quartet" seen in the ^{19}F NMR spectrum of compound **4** is due to overlapping of two triplets. The symmetric compounds **3**, **5**, **6** and **8** show quintets attributed to antiparallel conformers. Asymmetric compounds **4** and **7** display sextets due to overlapping of two quintets. Other signals stem from the parallel conformers; asterisks denote signals of grease (impurity).

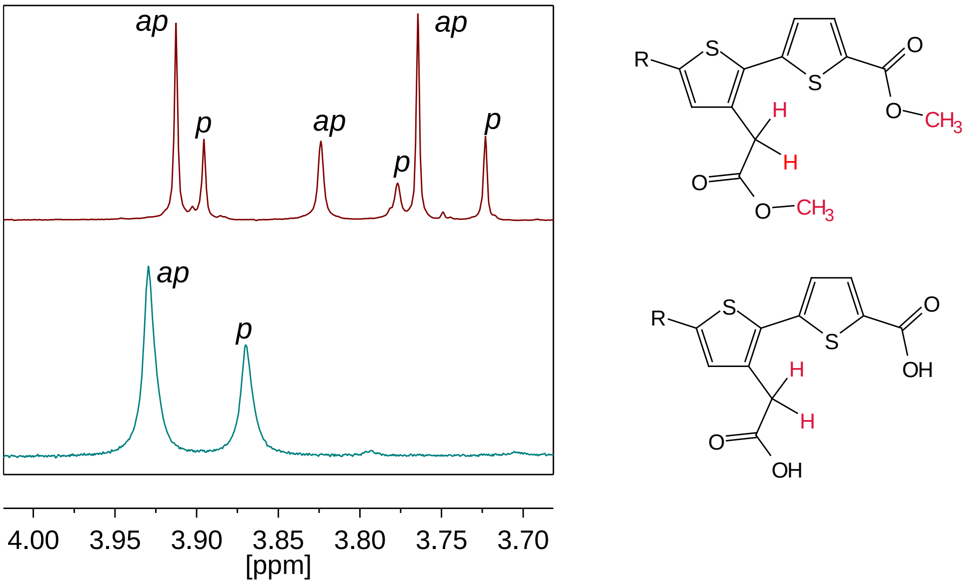

Figure S2: Left: sections of the ^{1}H NMR spectrum of compounds **SyTh₂** (top, in $CDCl_3$) and **SyTh₂-H** (bottom, in DMF-d_7). After hydrolysis of the ester groups, four of the six singlets disappeared and only the methylene singlets (p/ap) were observed. Right: structures of bithiophene residues with (highlighted) groups observed between 4.00 and 3.70 ppm.

NMR spectra, HPLC traces and photophysical data of new compounds

Compound 3

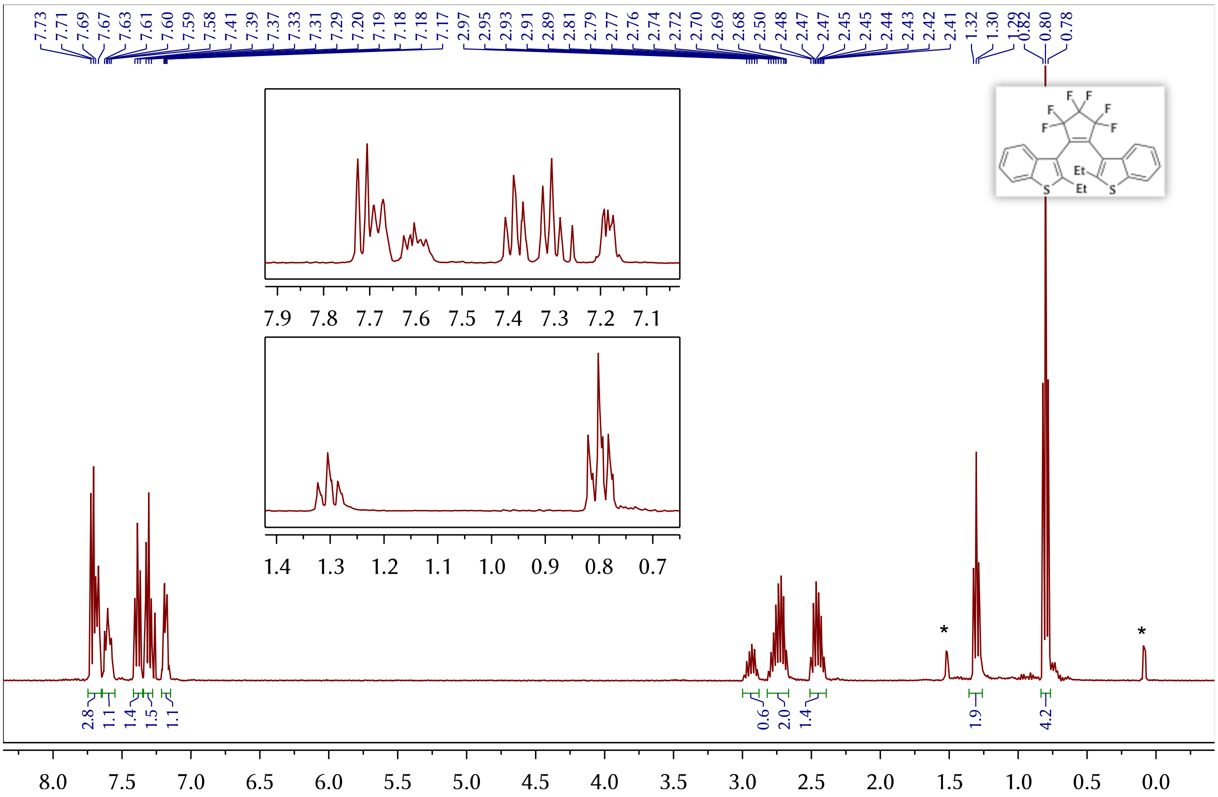

Figure S3: ^{1}H NMR spectrum of compound **3** in $CDCl_3$. Asterisks denote signals of TMS and water.

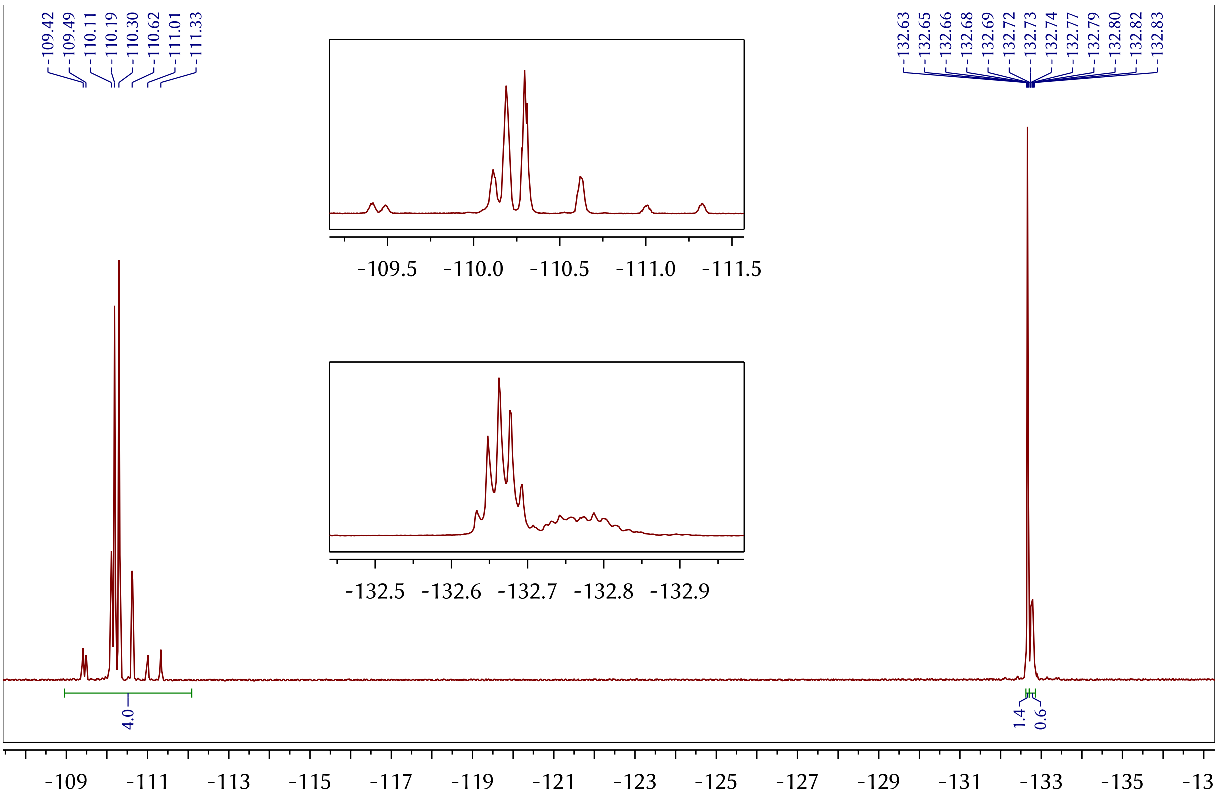

Figure S4: ^{19}F NMR spectrum of compound **3** in $CDCl_3$.

Compound 4

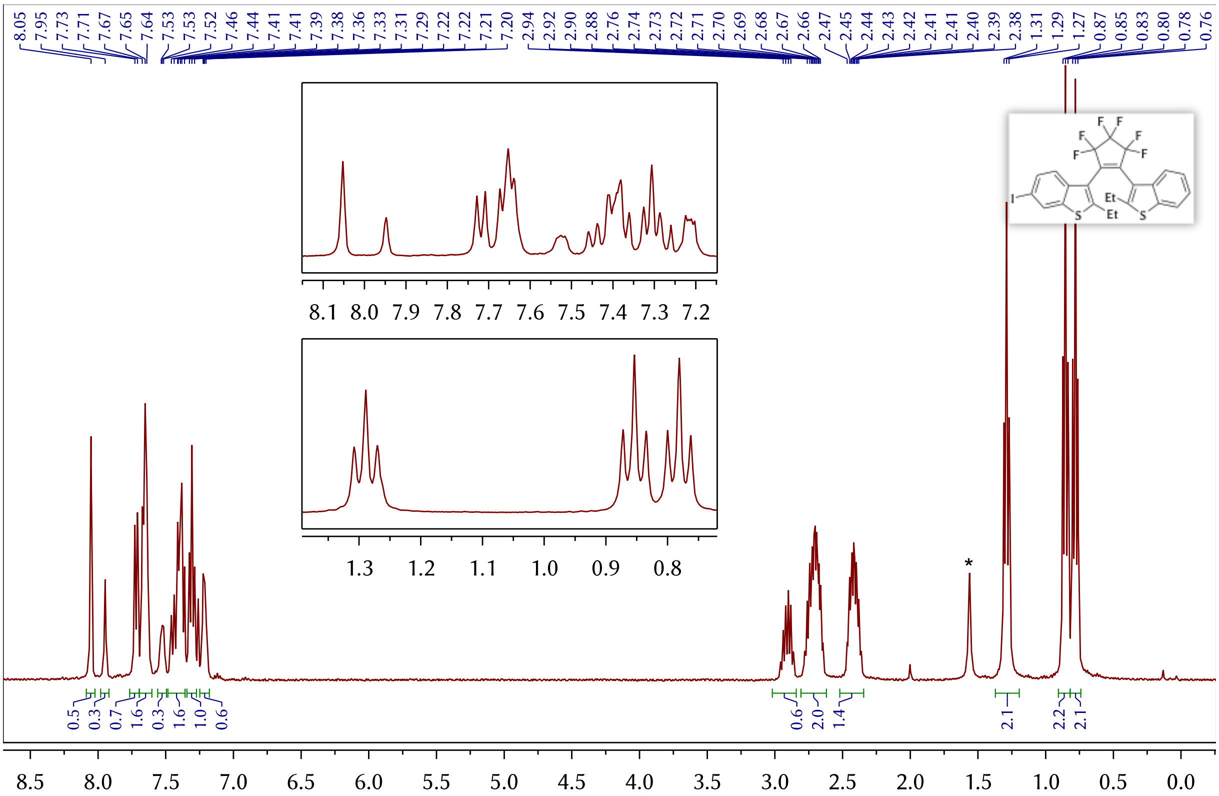

Figure S5: ^{1}H NMR spectrum of compound **4** in $CDCl_3$. The asterisk denotes the signal of water.

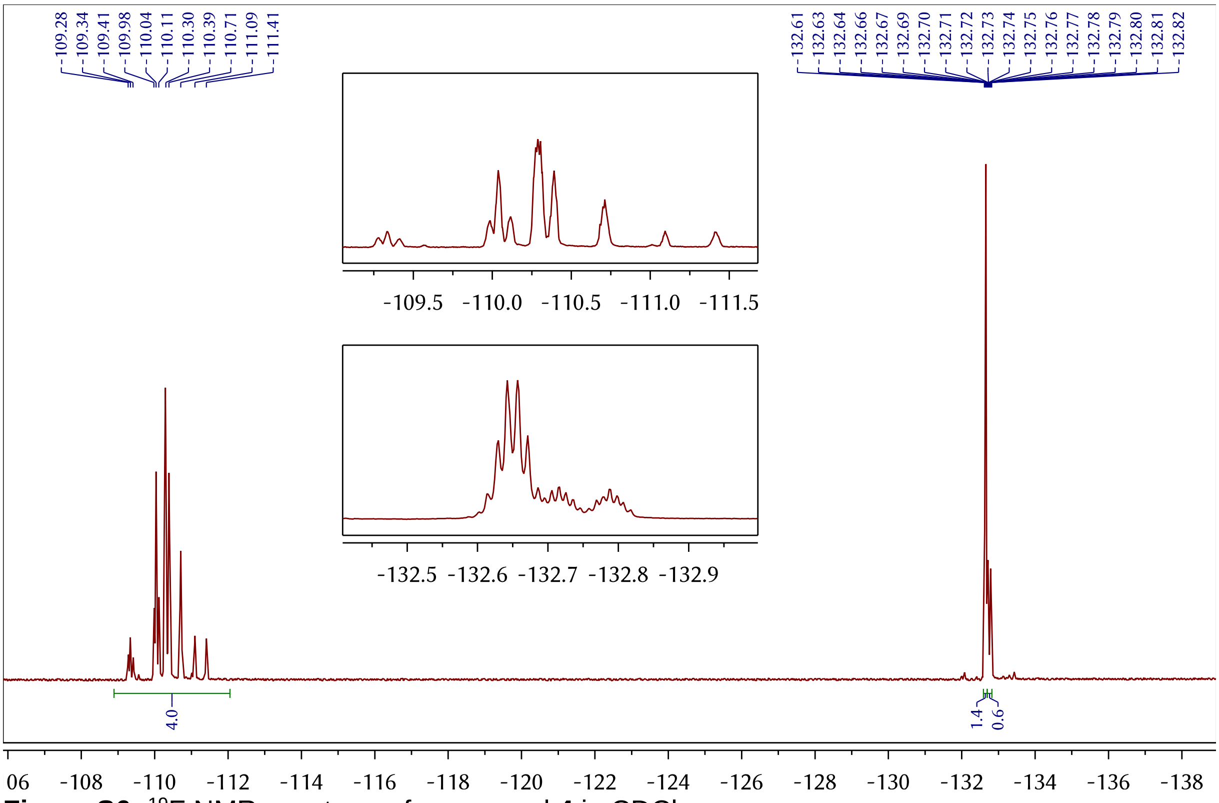

Figure S6: ^{19}F NMR spectrum of compound **4** in $CDCl_3$.

Compound 5

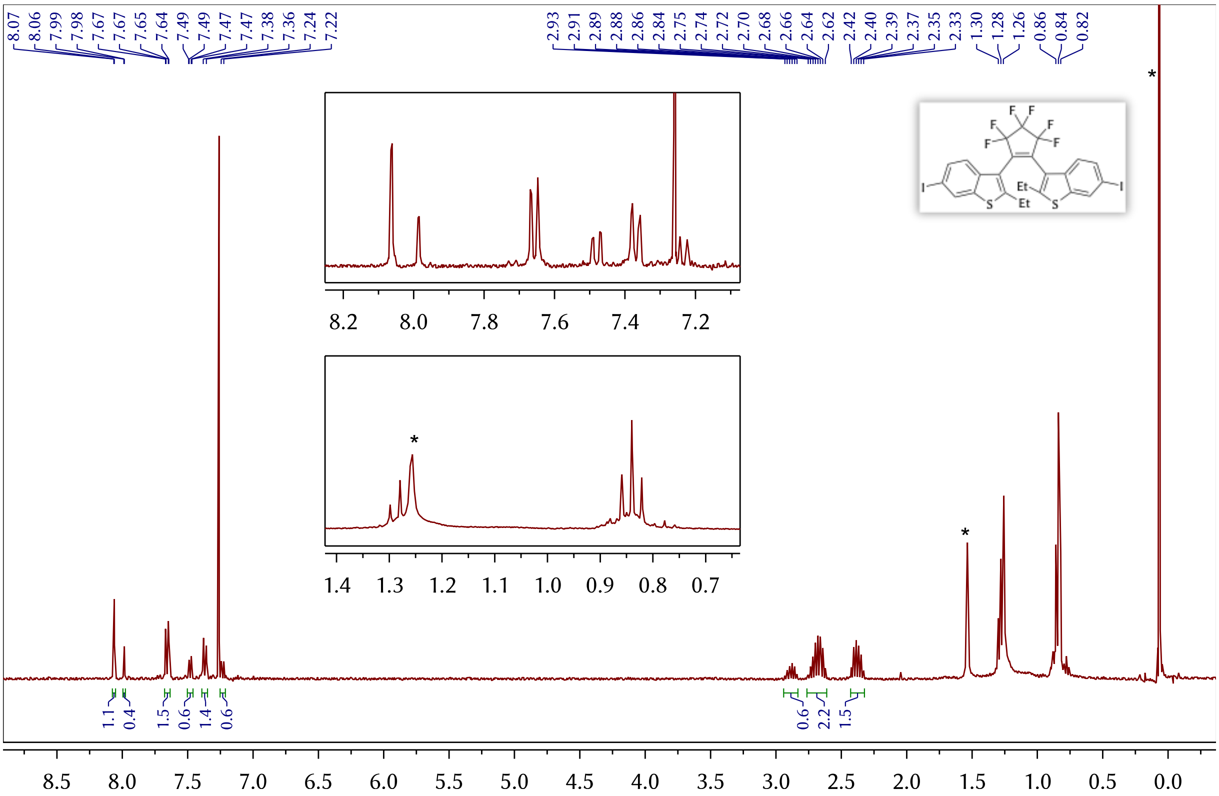

Figure S7: ^{1}H NMR spectrum of compound **5** in $CDCl_3$. Asterisks denote signals of TMS, grease, and water.

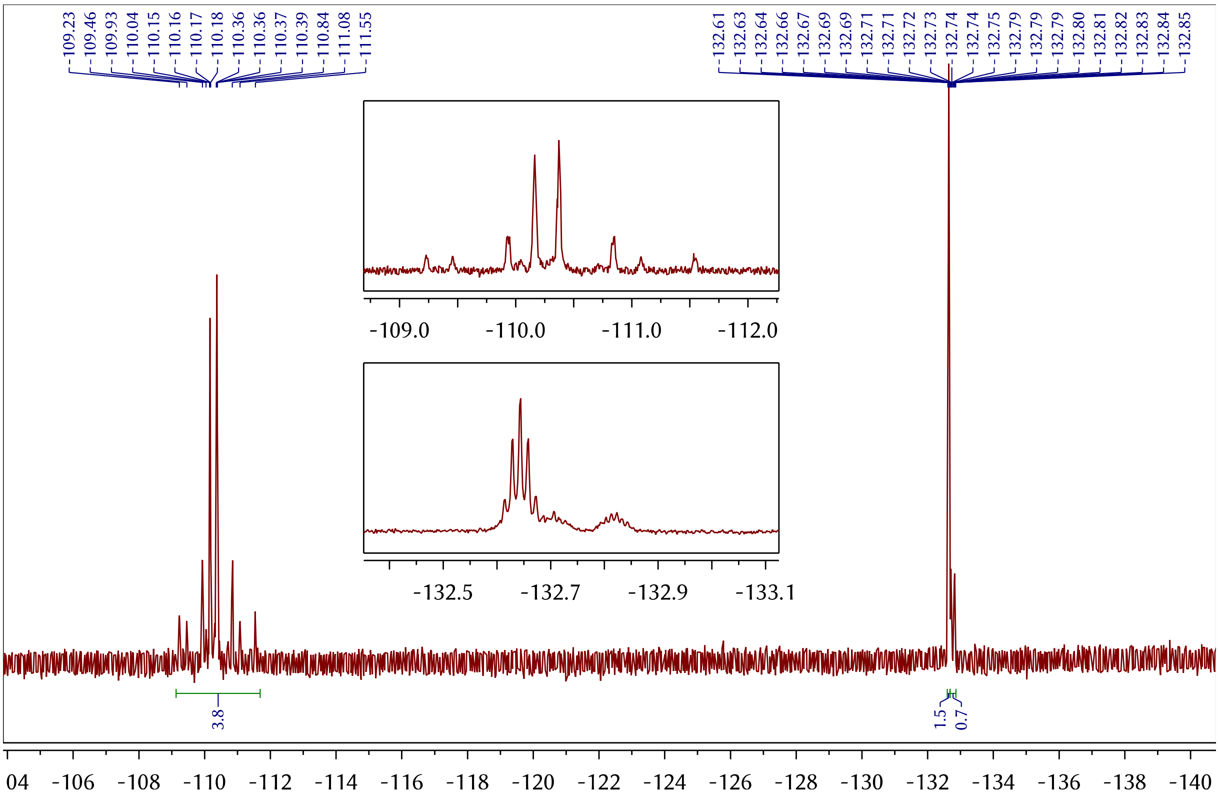

Figure S8: ^{19}F NMR spectrum of compound **5** in $CDCl_3$.

Compound 6

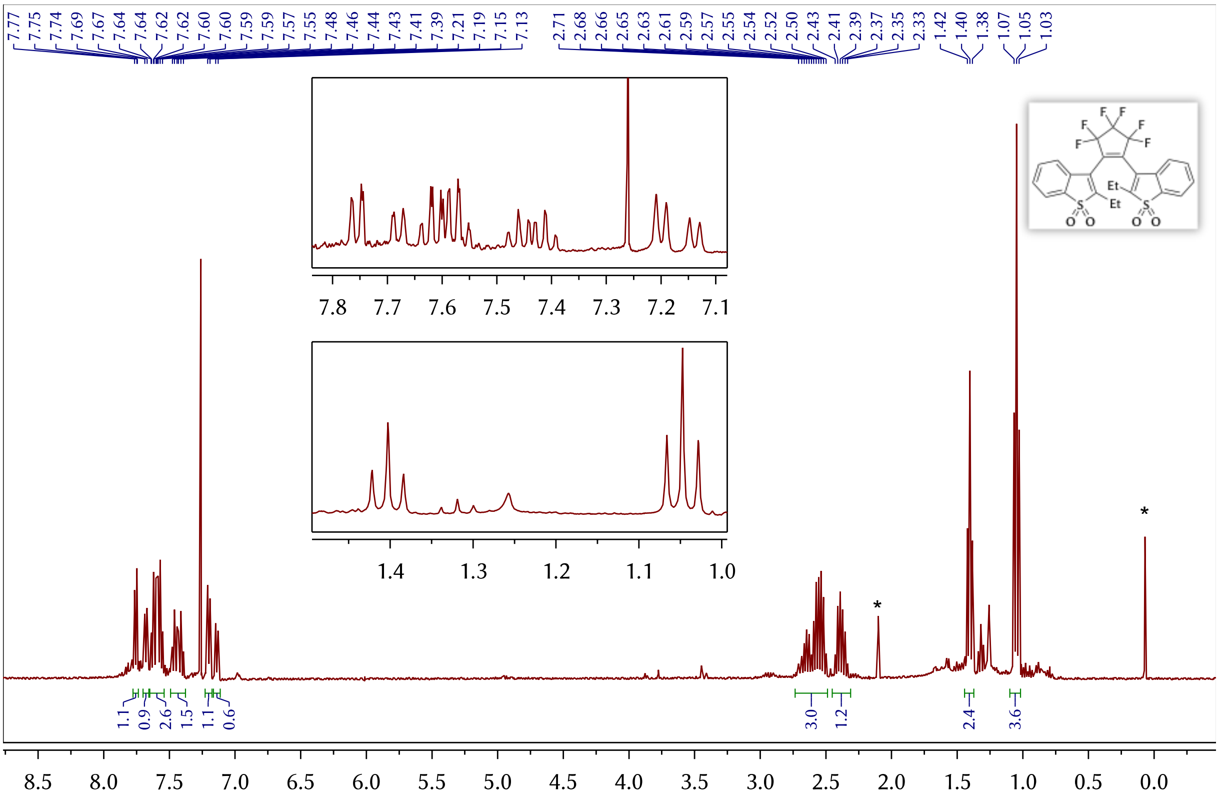

Figure S9: ^{1}H NMR spectrum of compound **6** in $CDCl_3$. Asterisks denote signals of TMS and acetic acid.

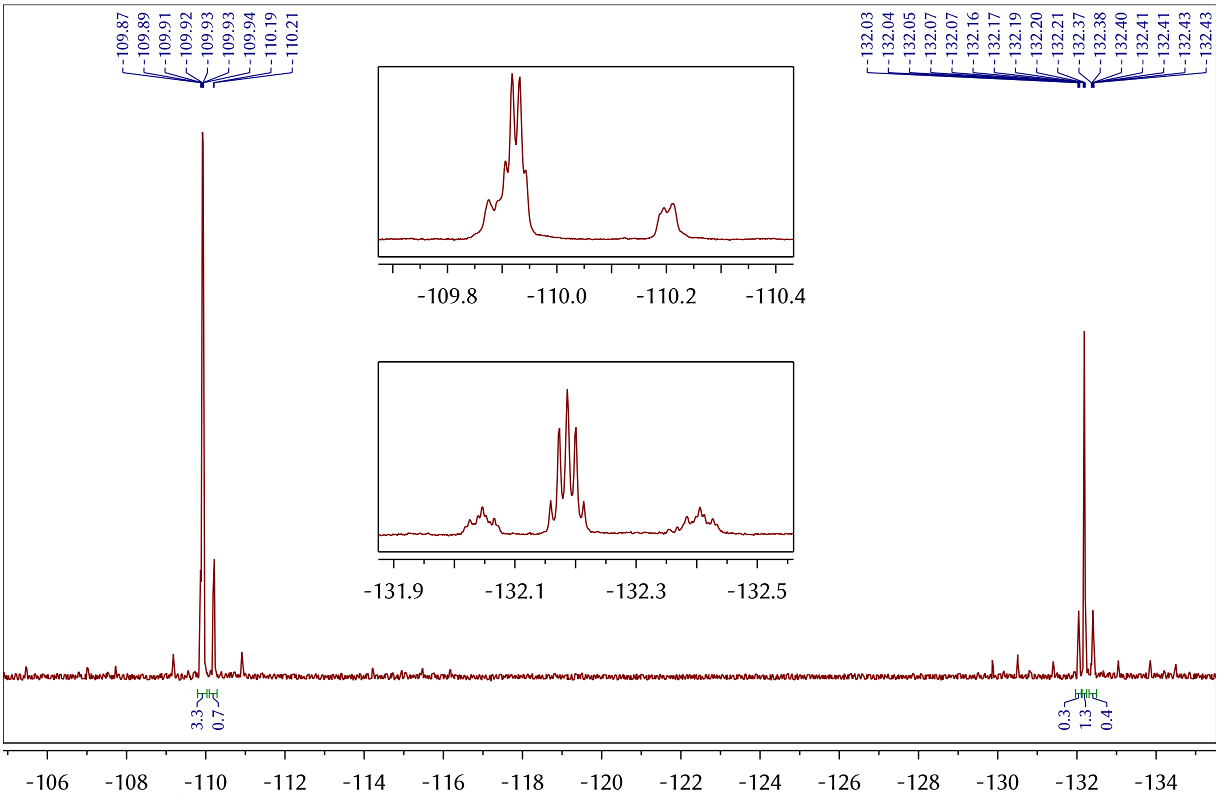

Figure S10: ^{19}F NMR spectrum of compound **6** in $CDCl_3$.

Compound 7

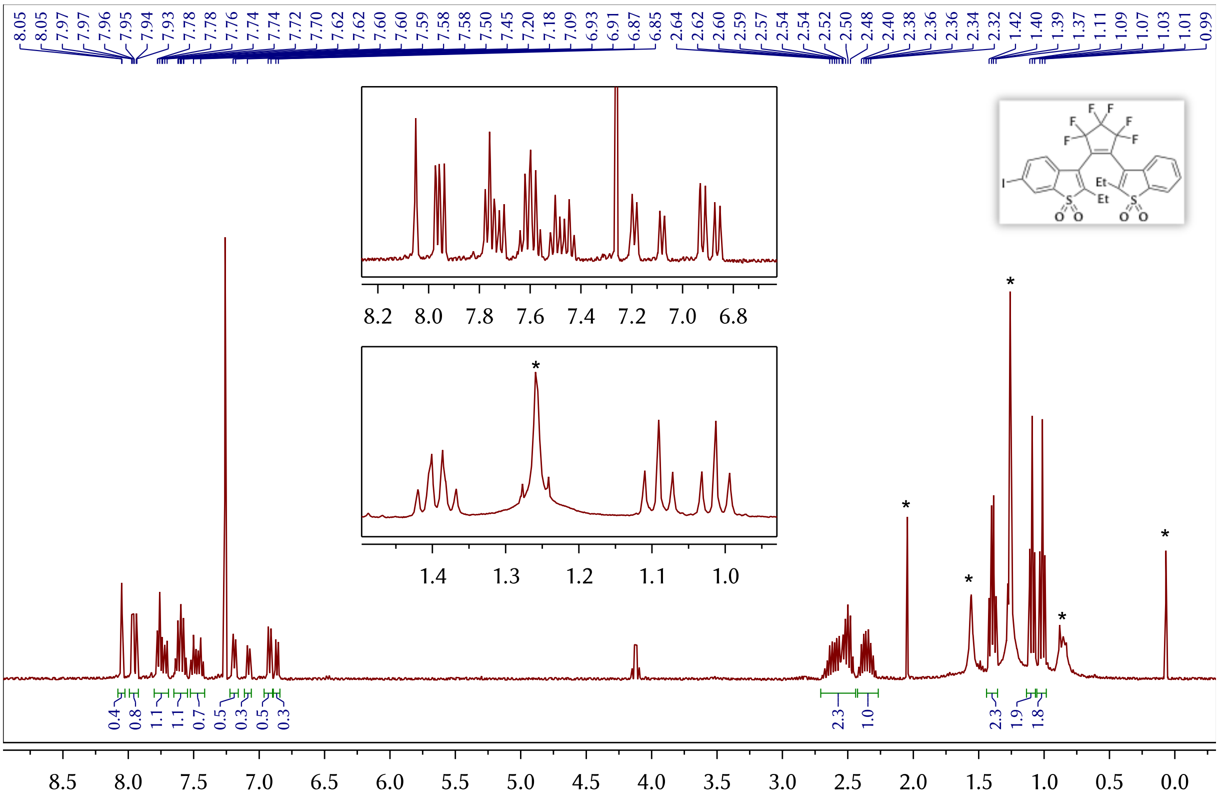

Figure S11: ^{1}H NMR spectrum of compound **7** in $CDCl_3$. Asterisks denote signals of TMS, grease, water, and acetonitrile.

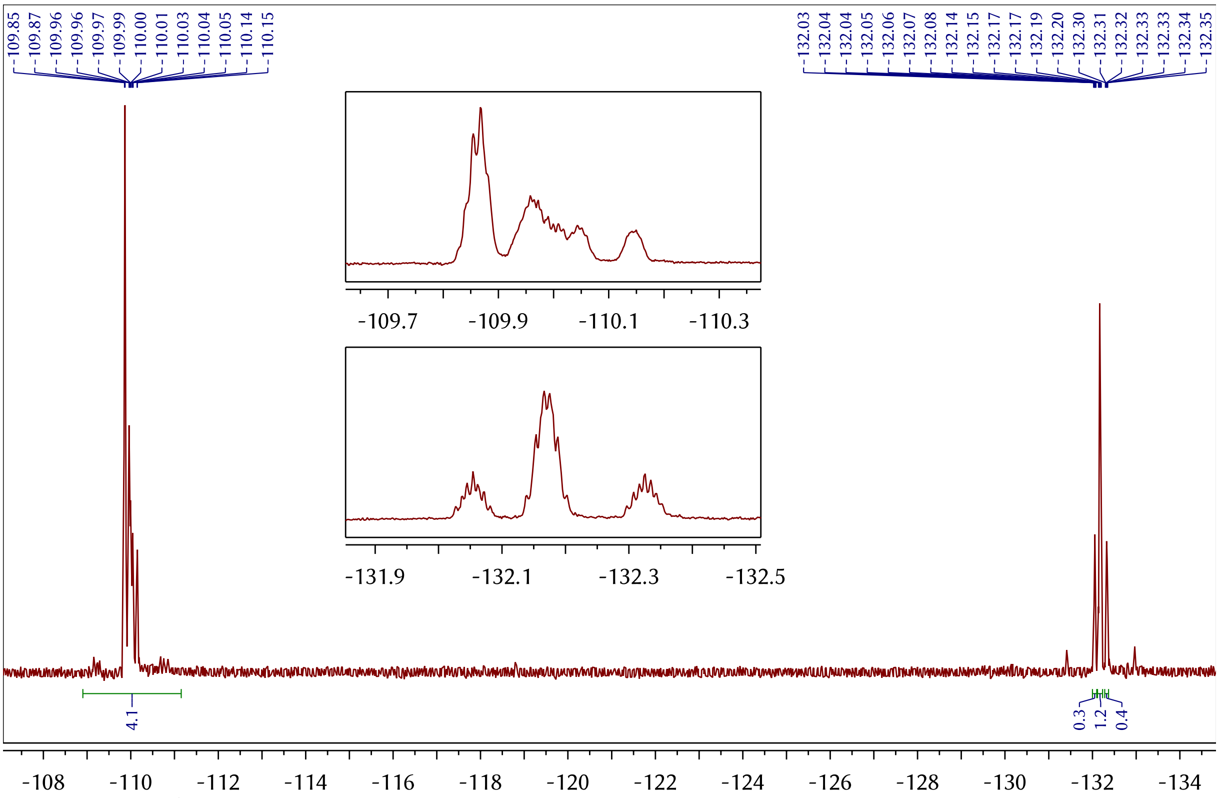

Figure S12: ^{19}F NMR spectrum of compound **7** in $CDCl_3$.

Compound 8

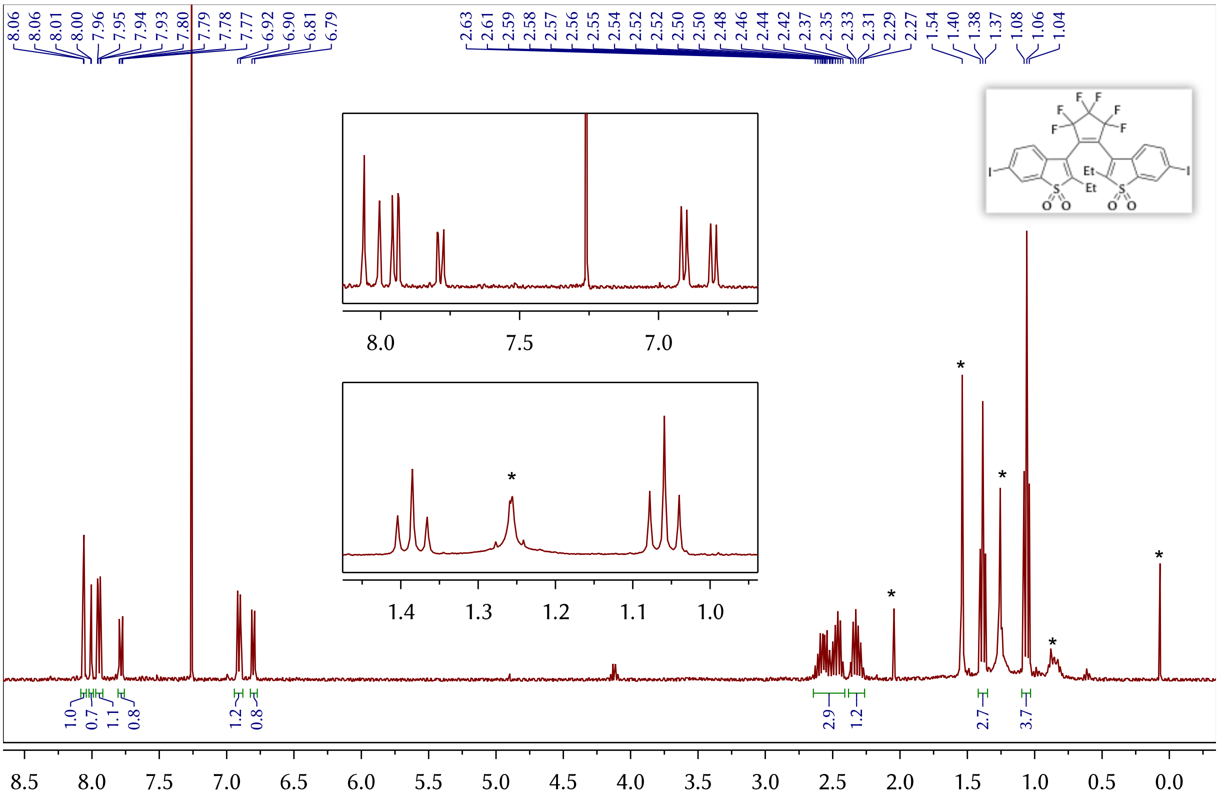

Figure S13: ^{1}H NMR spectra of compound **8** in $CDCl_3$. Asterisks denote signals of TMS, grease, water, and acetic acid.

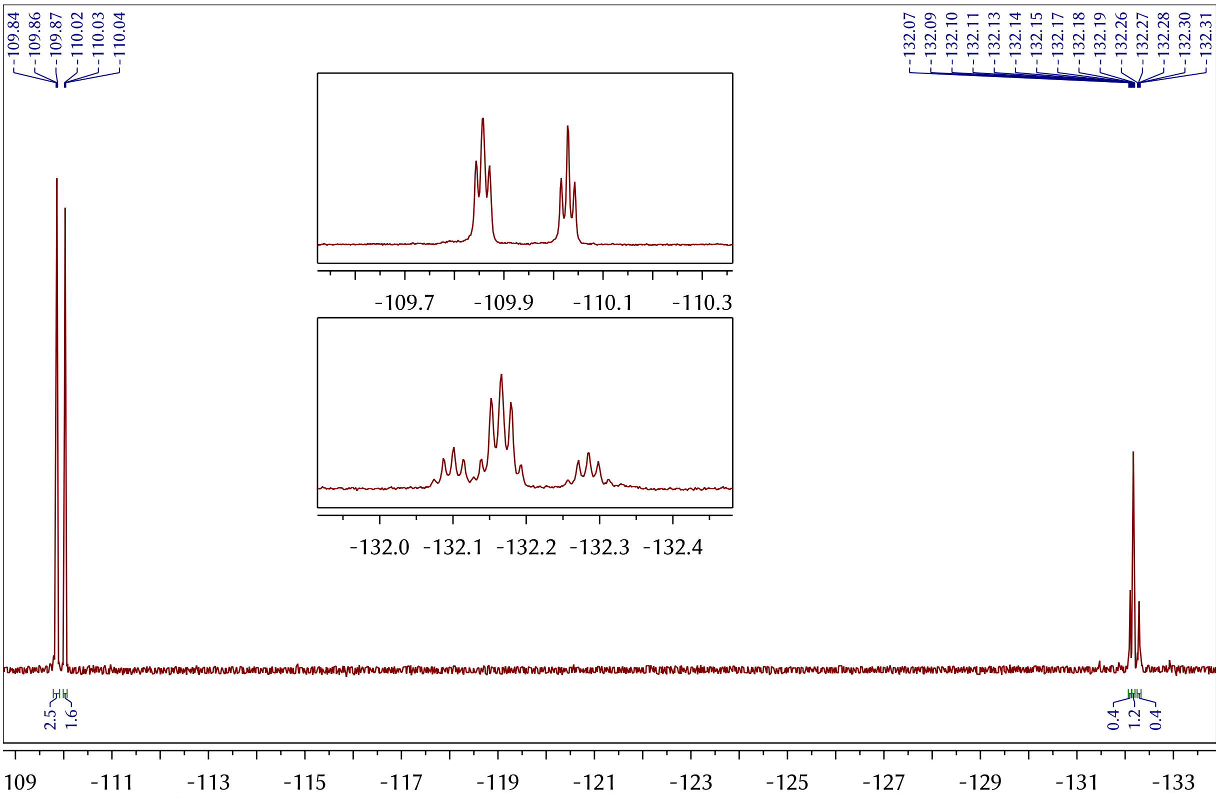

Figure S14: ^{19}F NMR spectrum of compound **8** in $CDCl_3$.

Compound AsTh₁

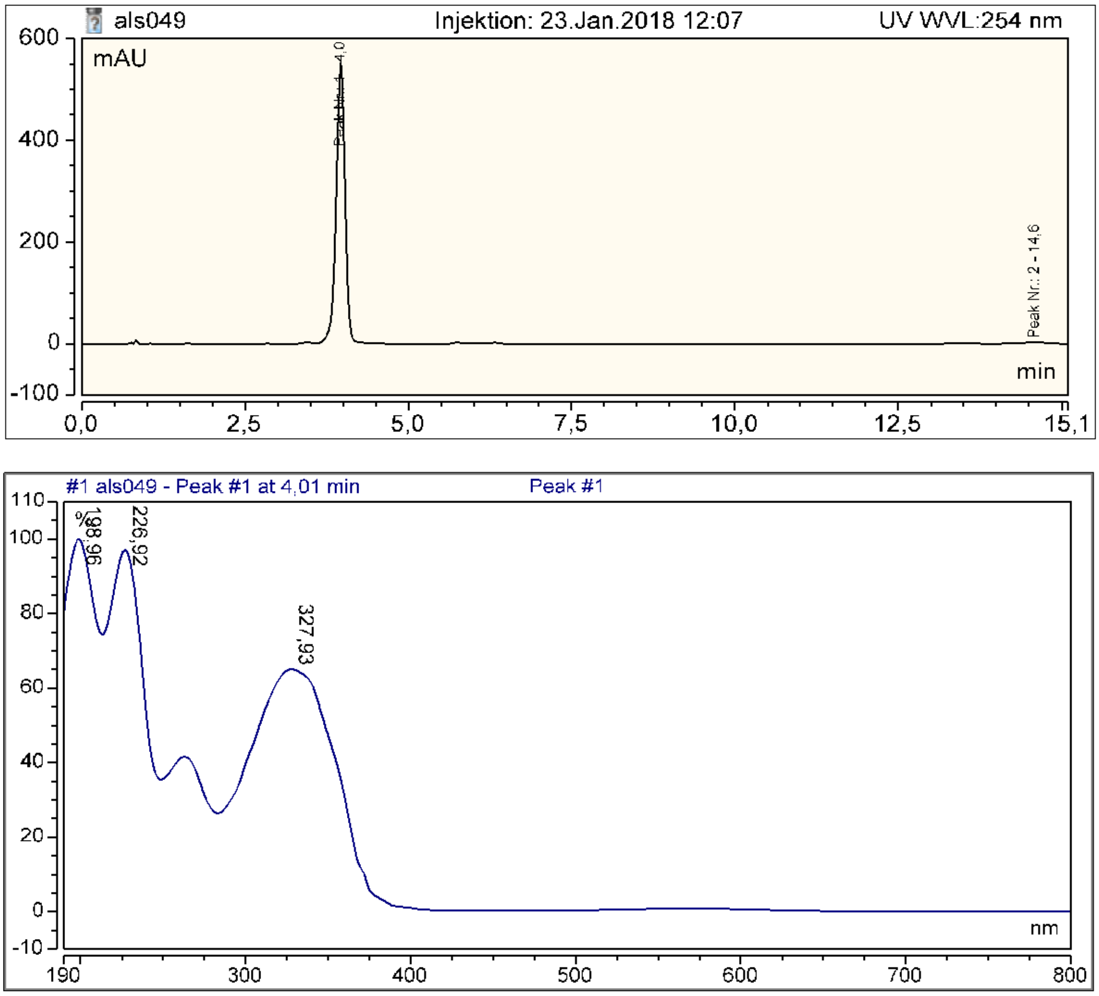

Figure S15: Analytical HPLC trace of compound **AsTh₁**.

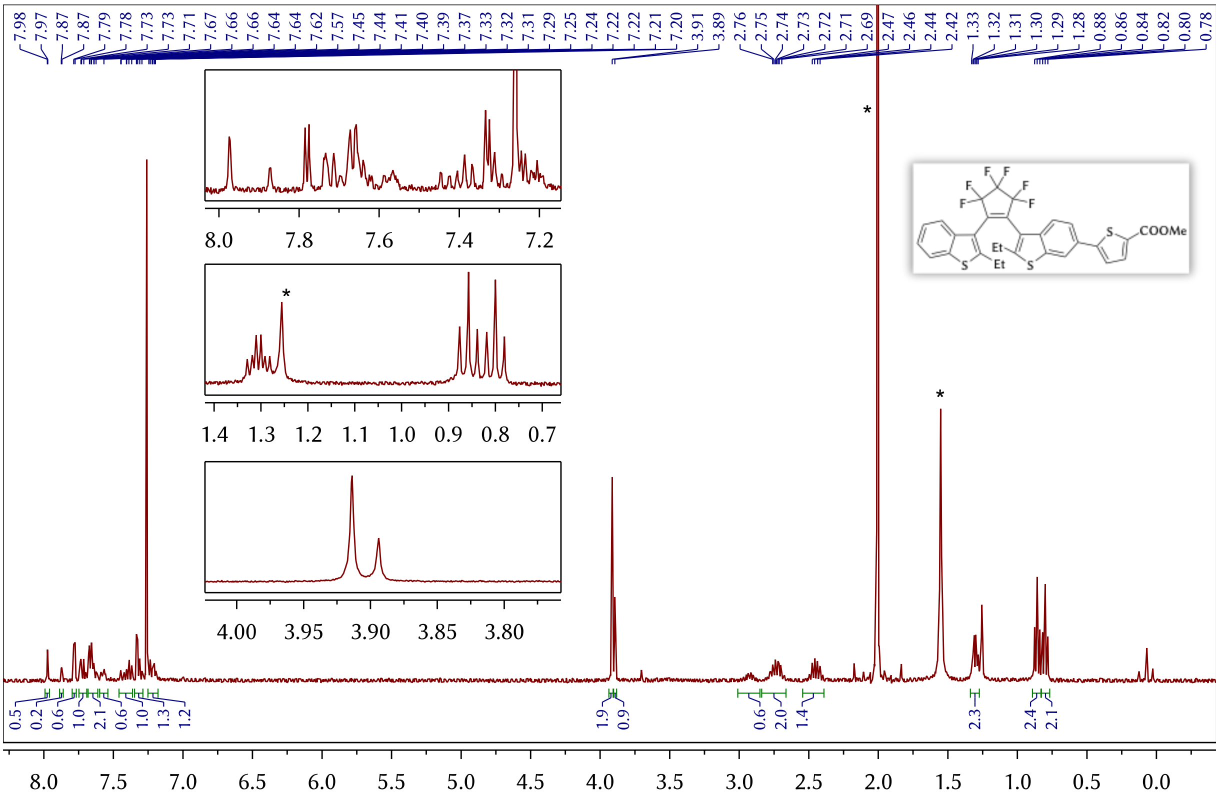

Figure S16: ^{1}H NMR spectrum of compound **AsTh$_1$** in $CDCl_3$. Asterisks denote signals of TMS, grease, water, and acetonitrile.

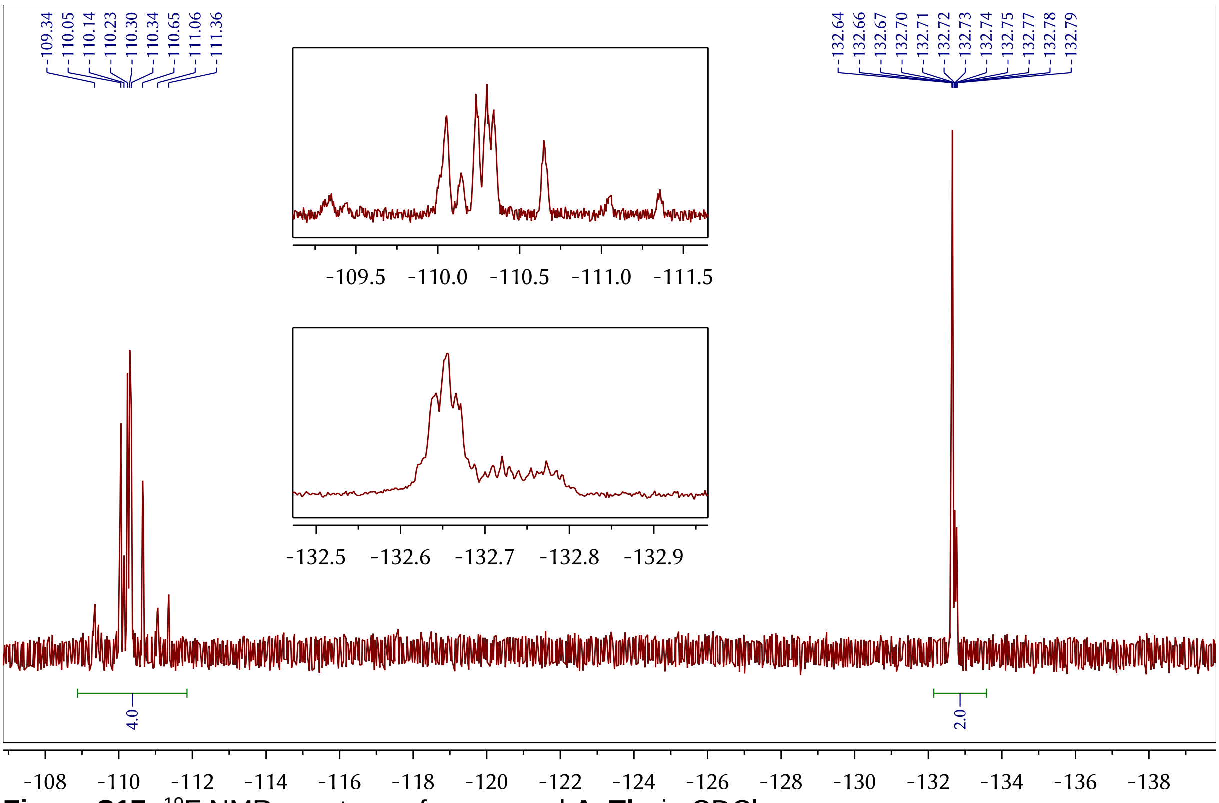

Figure S17: ^{19}F NMR spectrum of compound **AsTh$_1$** in $CDCl_3$.

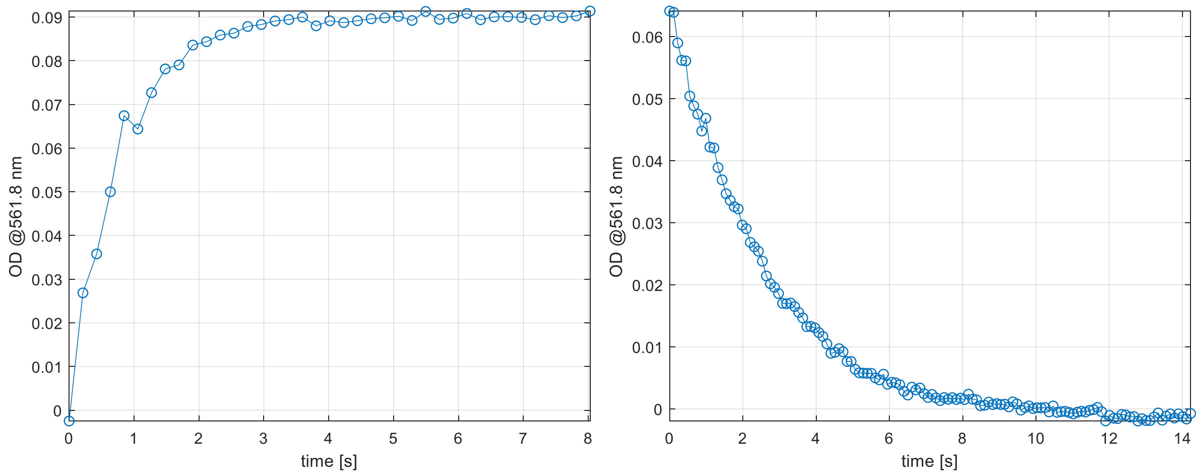

Figure S18: Absorption of **AsTh$_1$** in MeCN at ca. 560 nm during the cyclization (left, irradiation at 365 nm) and the cycloreversion (right, irradiation at 470 nm).

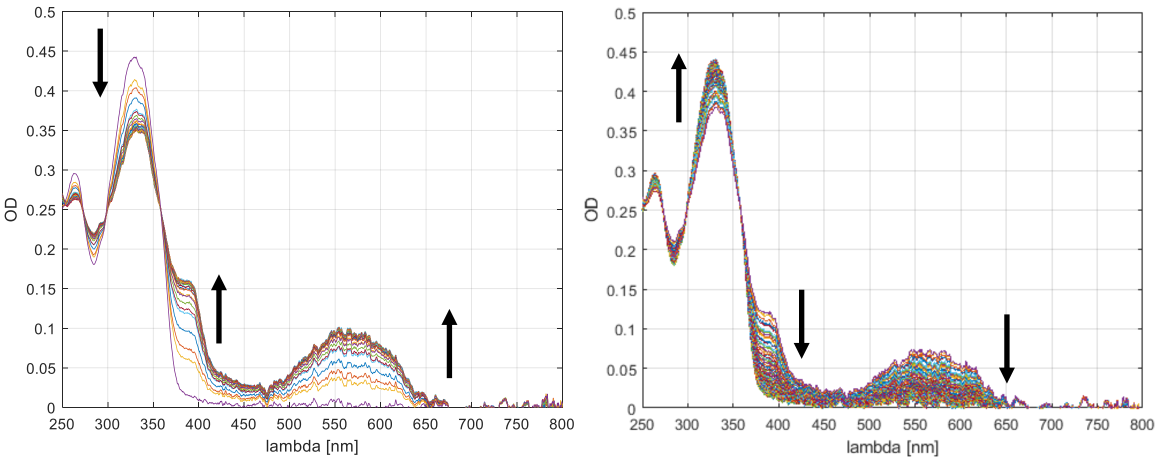

Figure S19: Absorption of **AsTh$_1$** in MeCN during the cyclization (left, irradiation at 365 nm) and the cycloreversion (right, irradiation at 470 nm).

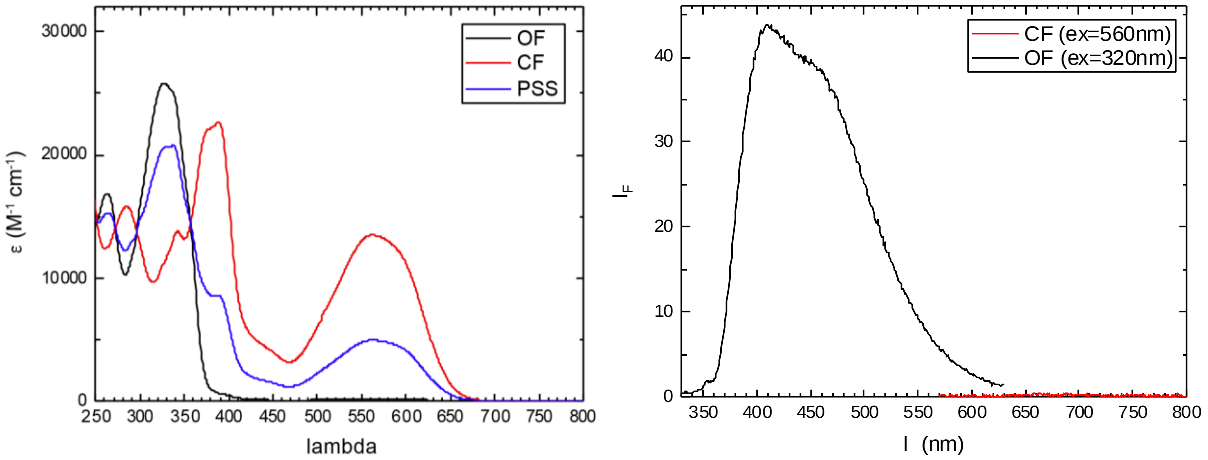

Figure S20: Absorption (left) and emission (right) spectra of **AsTh$_1$** in MeCN, as the open form (OF), closed form (CF), and the photo-stationary state at 365 nm (PSS).

Compound SyTh$_1$

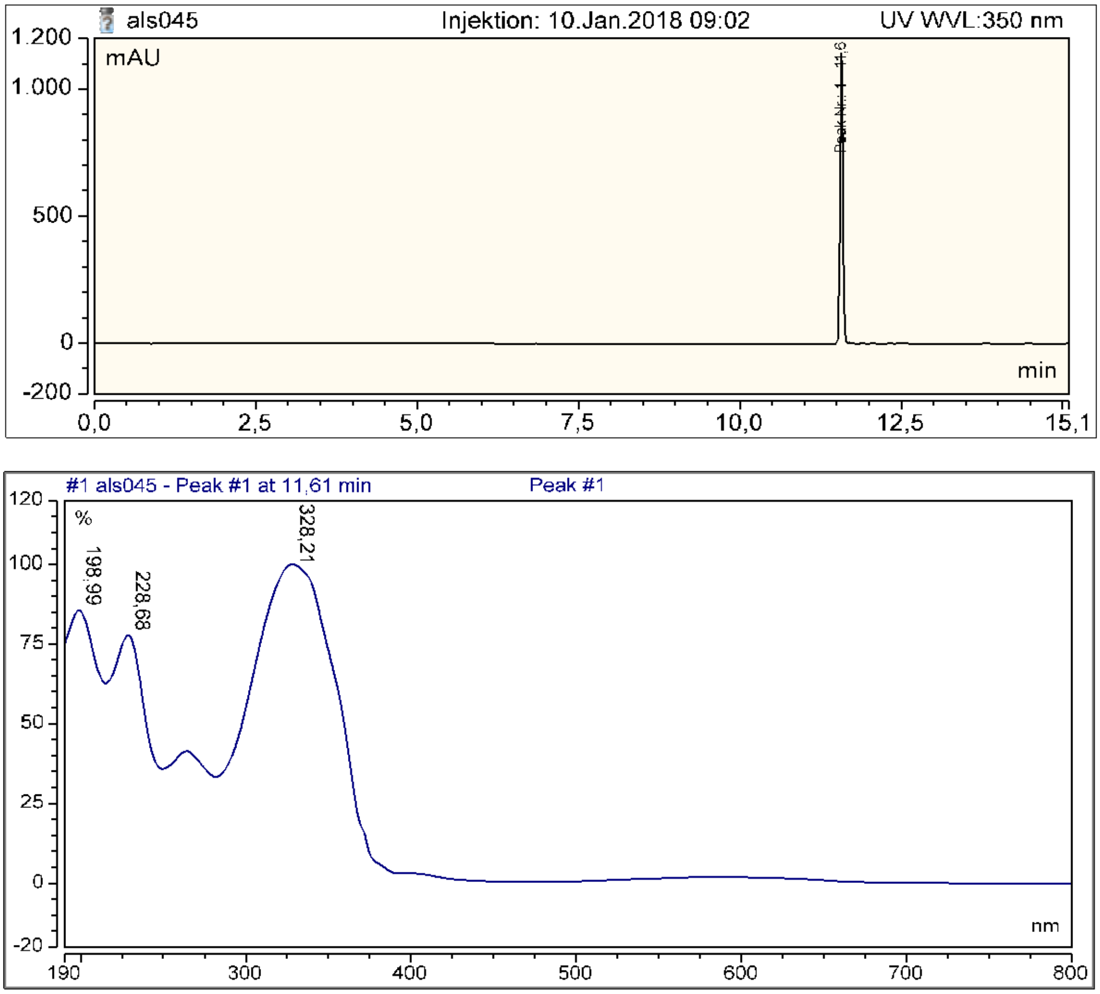

Figure S21: Analytical HPLC trace of compound **SyTh$_1$**.

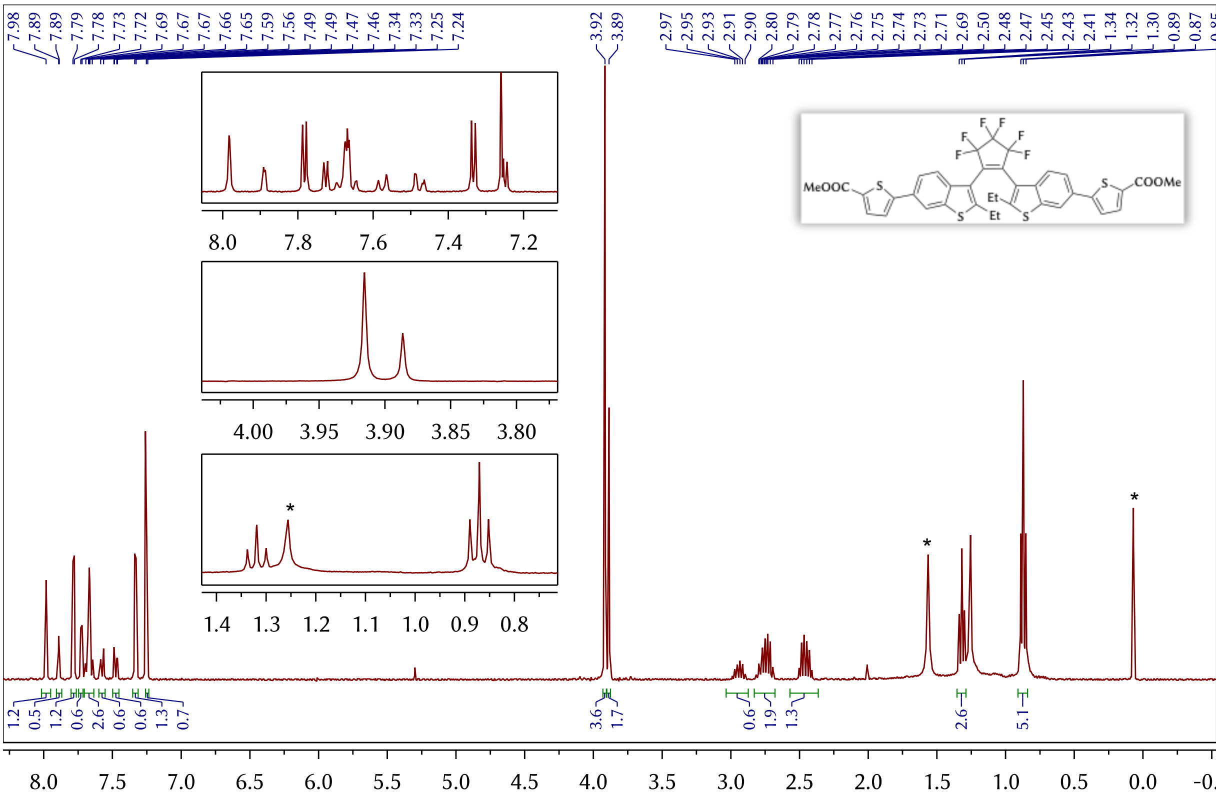

Figure S22: ^{1}H NMR spectrum of compound **SyTh$_1$** in $CDCl_3$. Asterisks denote signals of TMS, grease, and water.

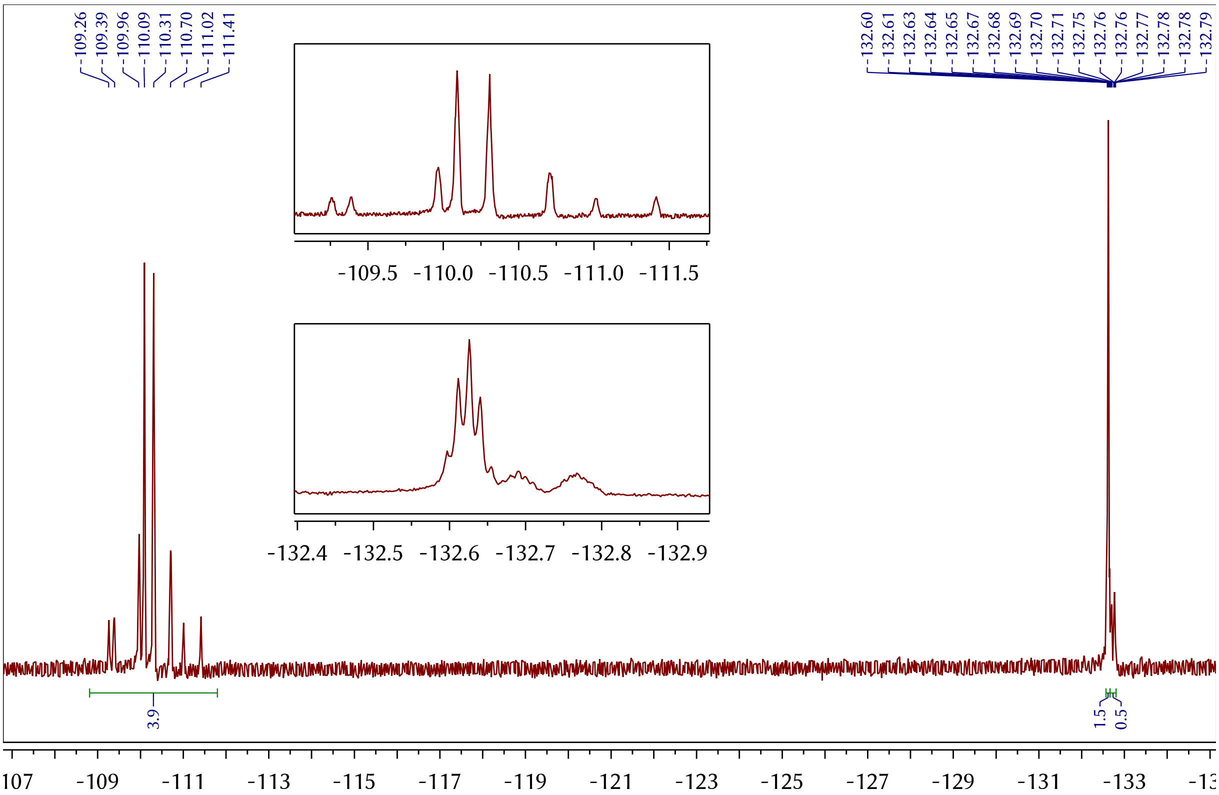

Figure S23: ^{19}F NMR spectrum of compound **SyTh$_1$** in $CDCl_3$.

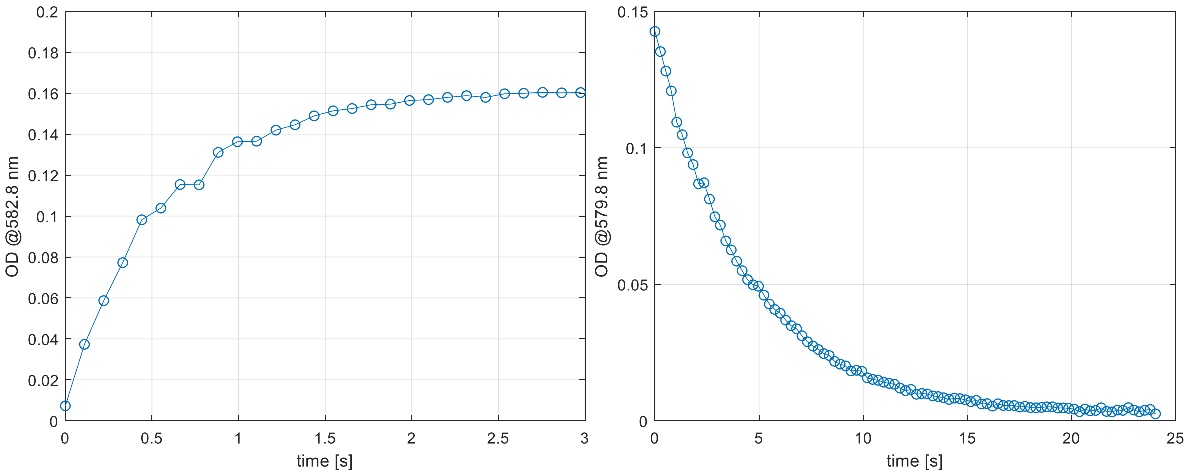

Figure S24: Absorption of **SyTh$_1$** in MeCN at ca. 580 nm during the cyclization (left, irradiation at 365 nm) and the cycloreversion (right, irradiation at 470 nm).

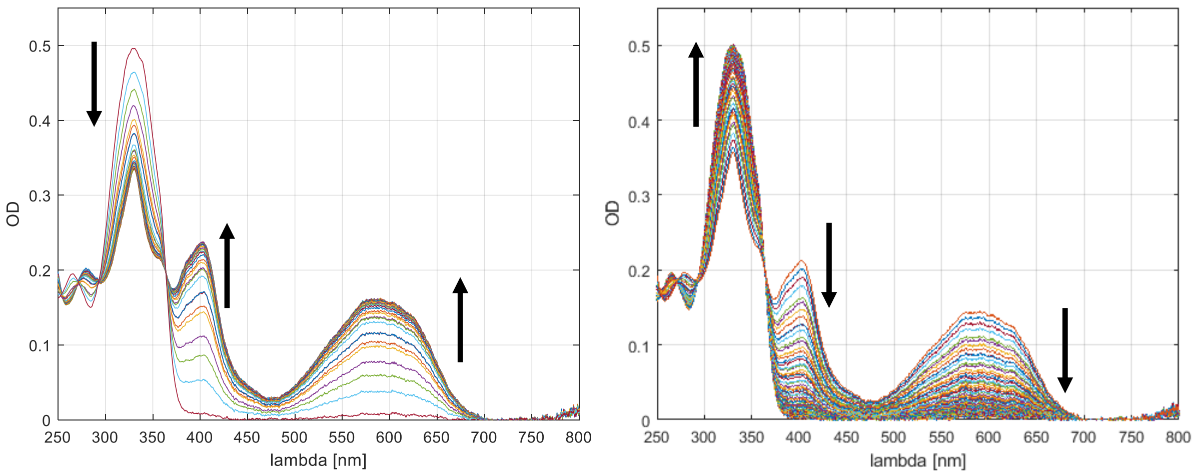

Figure S25: Absorption of **SyTh$_1$** in MeCN during the cyclization (left, irradiation at 365 nm) and the cycloreversion (right, irradiation at 470 nm).

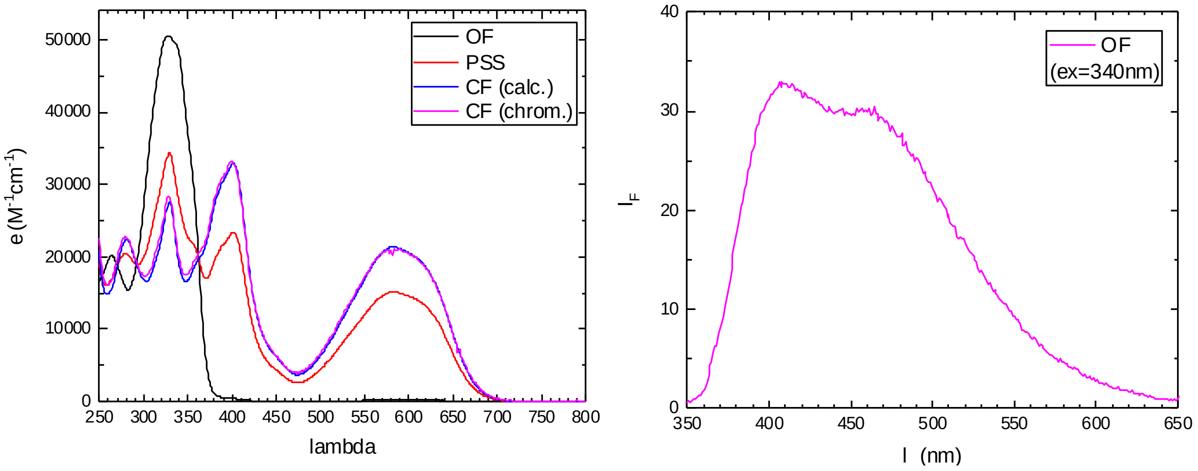

Figure S26: Absorption (left) and emission (right) spectra of compound **SyTh$_1$** in acetonitrile, as the open form (OF), closed form (CF), and the photo-stationary state at 365 nm (PSS). The spectrum of the CF obtained from the chromatogram is compared with the one calculated from the OF and the PSS, using the conversion in the PSS.

Compound AsOTh$_1$

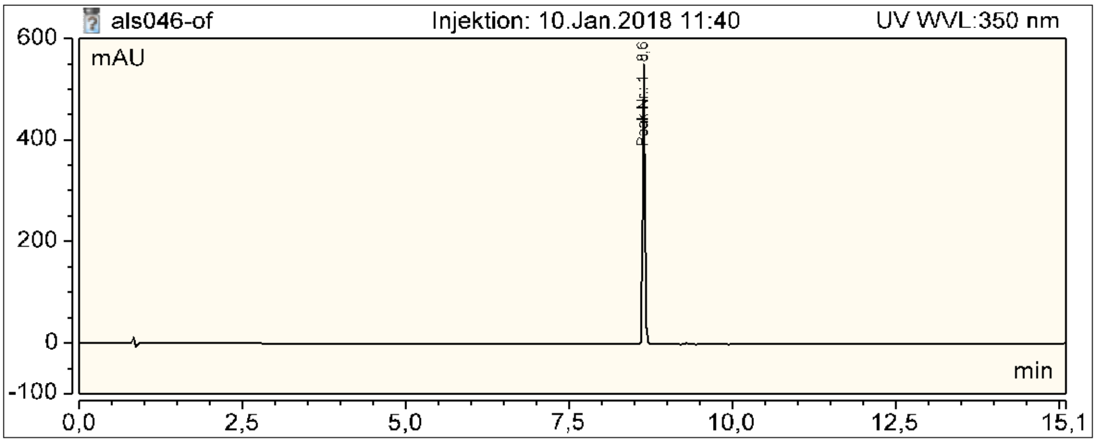

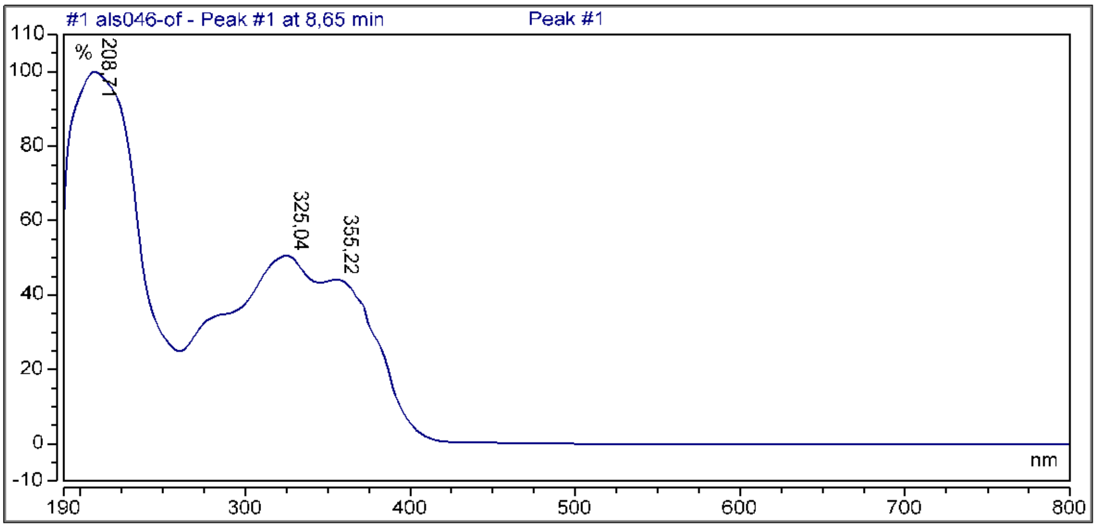

Figure S27: Analytical HPLC trace of compound **AsOTh$_1$**.

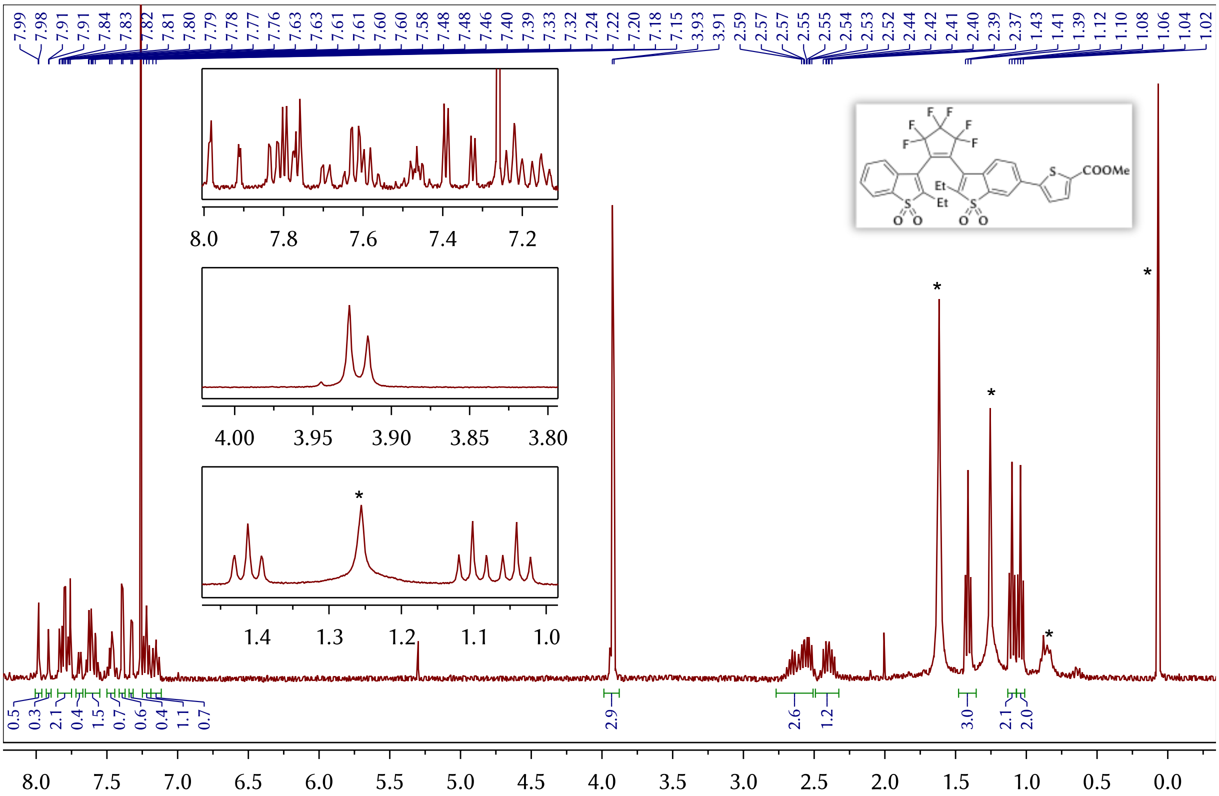

Figure S28: ^{1}H NMR spectrum of compound **AsOTh$_1$** in $CDCl_3$. Asterisks denote signals of TMS, grease, and water.

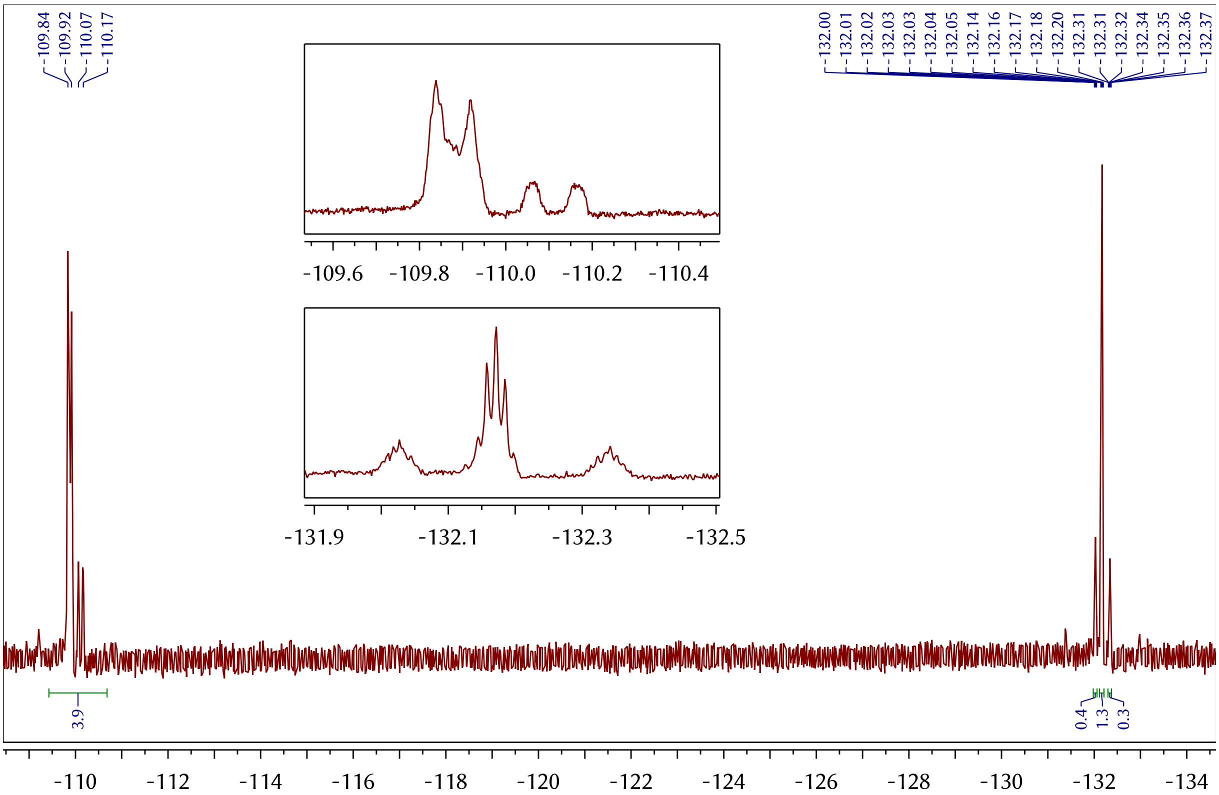

Figure S29: ^{19}F NMR spectrum of compound **AsOTh$_1$** in $CDCl_3$.

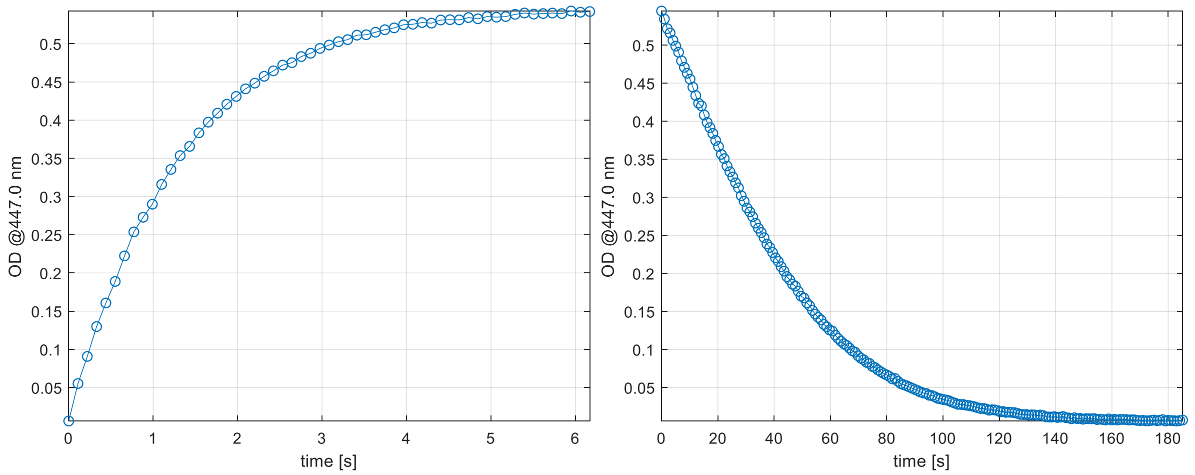

Figure S30: Absorption of **AsOTh$_1$** in MeCN at ca. 450 nm during the cyclization (left, irradiation at 365 nm) and the cycloreversion (right, irradiation at 470 nm).

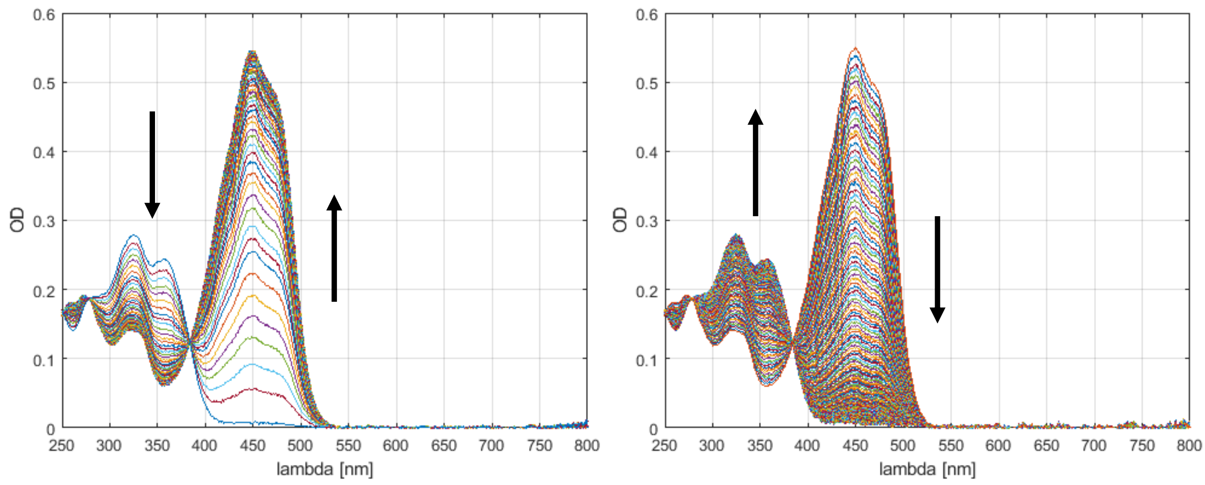

Figure S31: Absorption of **AsOTh$_1$** in MeCN during the cyclization (left, irradiation at 365 nm) and the cycloreversion (right, irradiation at 470 nm).

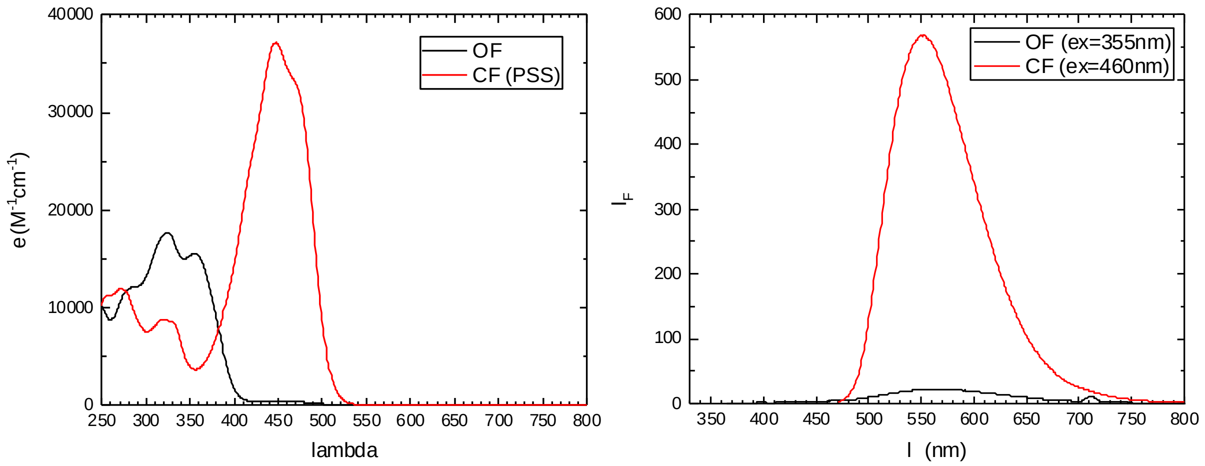

Figure S32: Absorption (left) and emission (right) spectra of compound **AsOTh$_1$** in MeCN, as the open form (OF) and the closed form (CF); in this case, the photo-stationary contains only the CF (conversion was complete).

Compound SyOTh$_1$

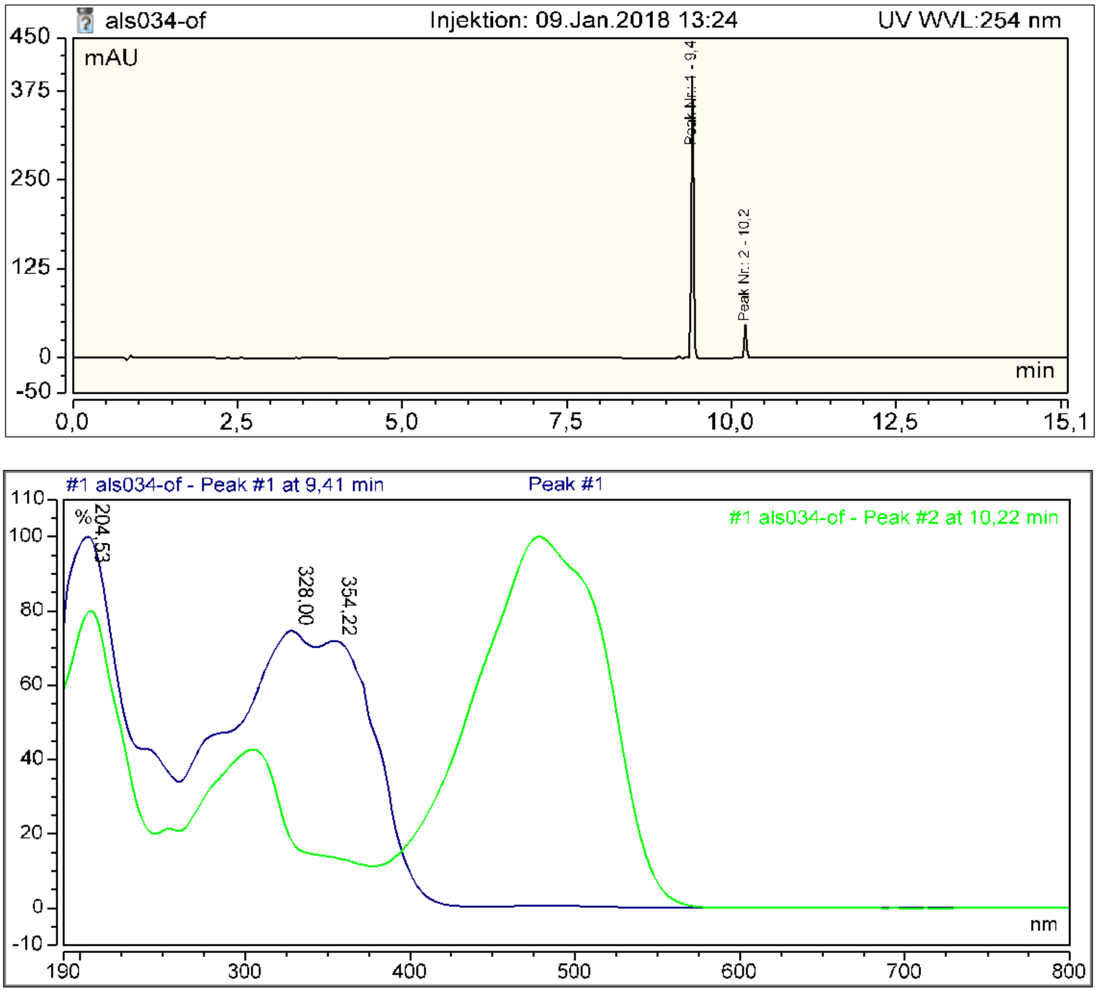

Figure S33: Analytical HPLC trace: peaks of the open and closed forms of compound **SyOTh$_1$**.

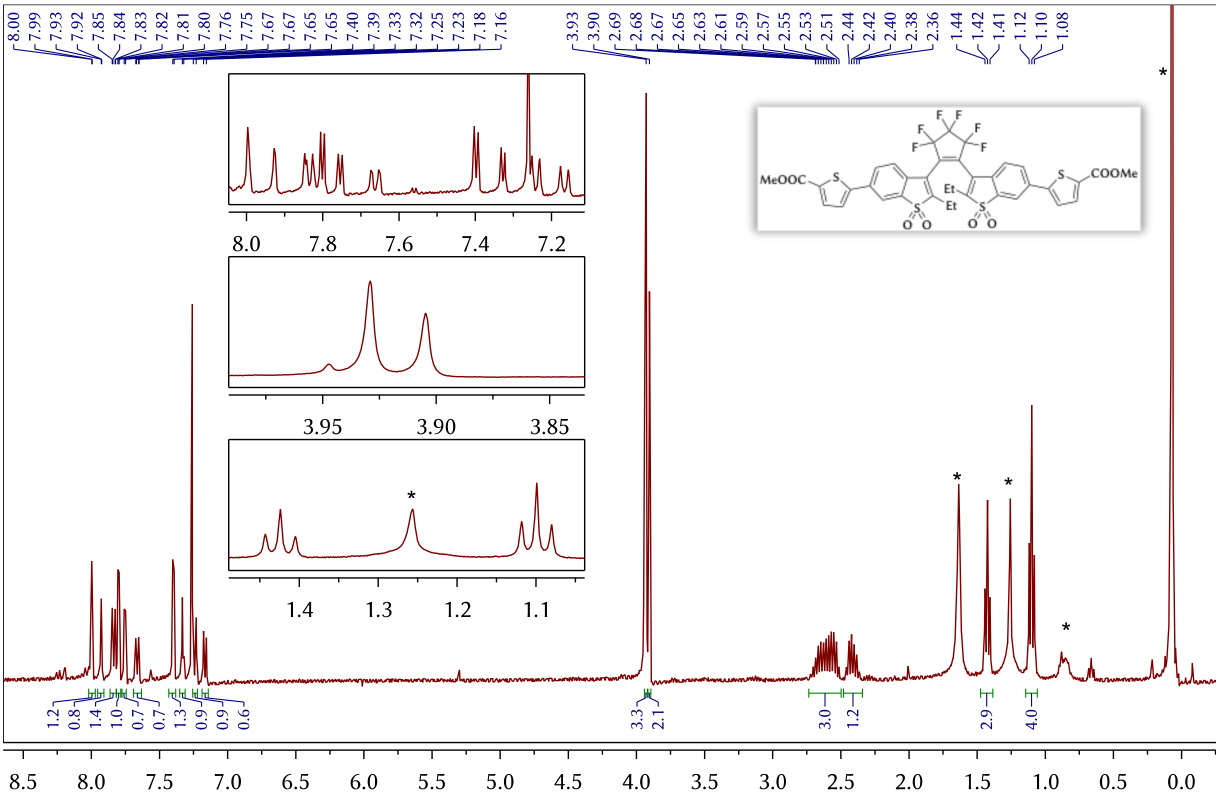

Figure S34: ^{1}H NMR spectrum of compound **SyOTh₁** in $CDCl_3$. Asterisks denote signals of TMS, grease, and water.

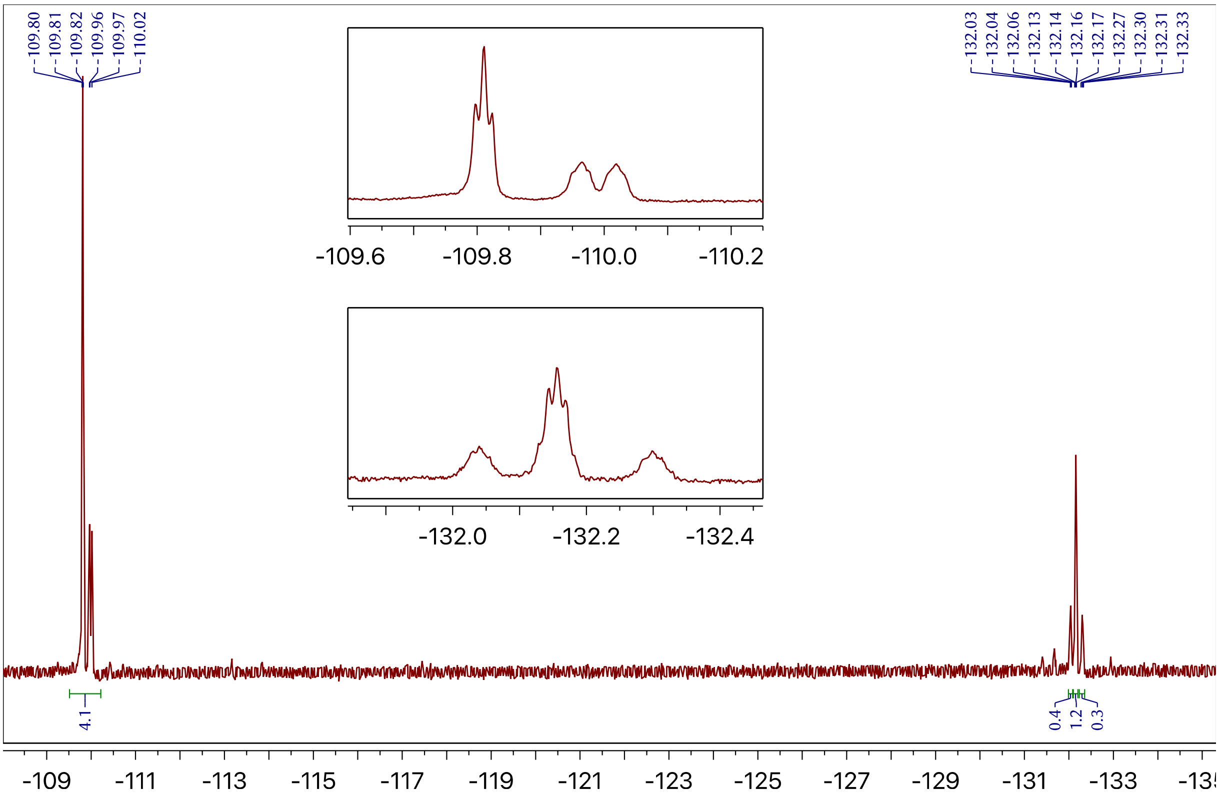

Figure S35: ^{19}F NMR spectrum of compound **SyOTh₁** in $CDCl_3$.

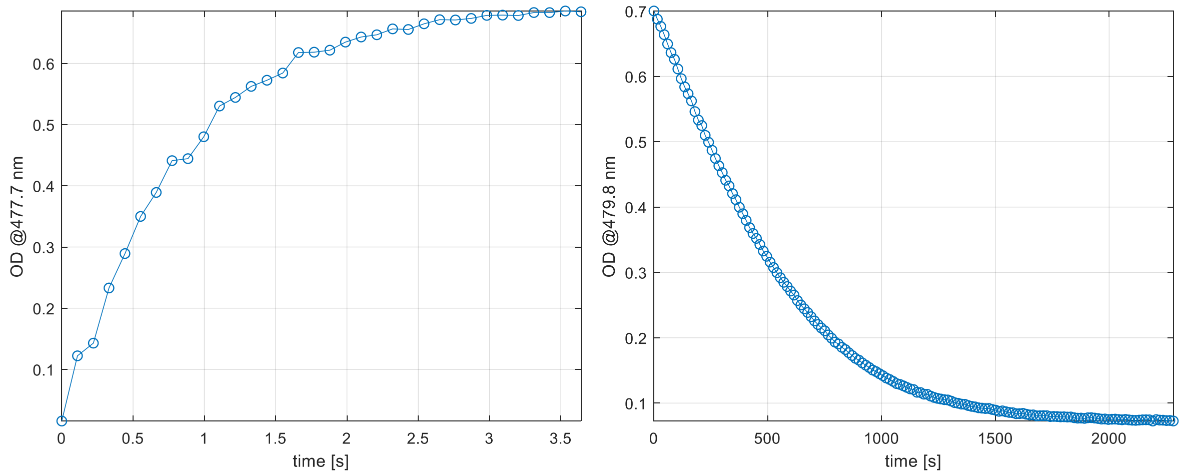

Figure S36: Absorption of **SyOTh$_1$** in MeCN at ca. 480 nm during the cyclization (left, irradiation at 365 nm) and the cycloreversion (right, irradiation at 470 nm).

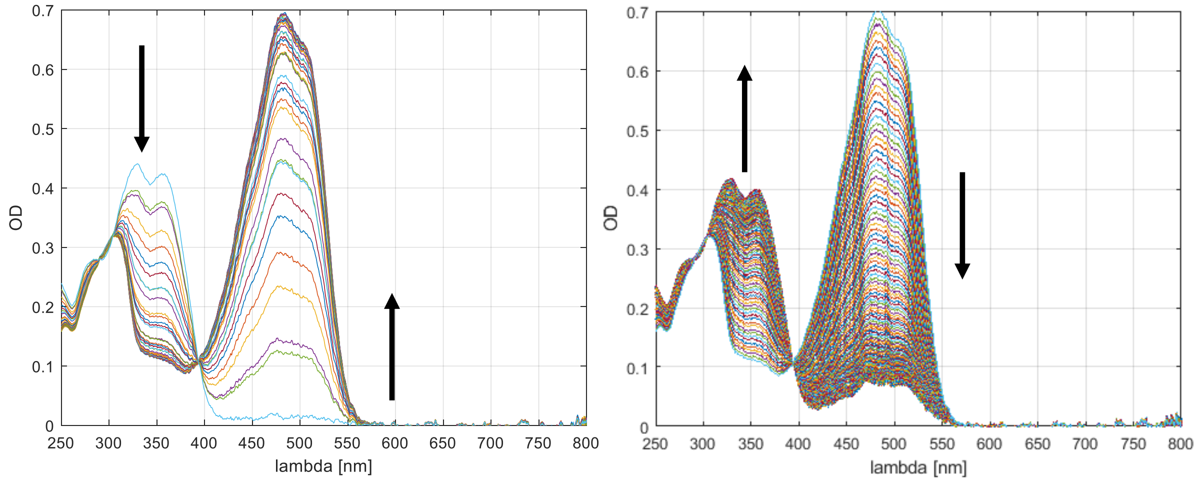

Figure S37: Absorption of **SyOTh$_1$** in MeCN during the cyclization (left, irradiation at 365 nm) and the cycloreversion (right, irradiation at 470 nm).

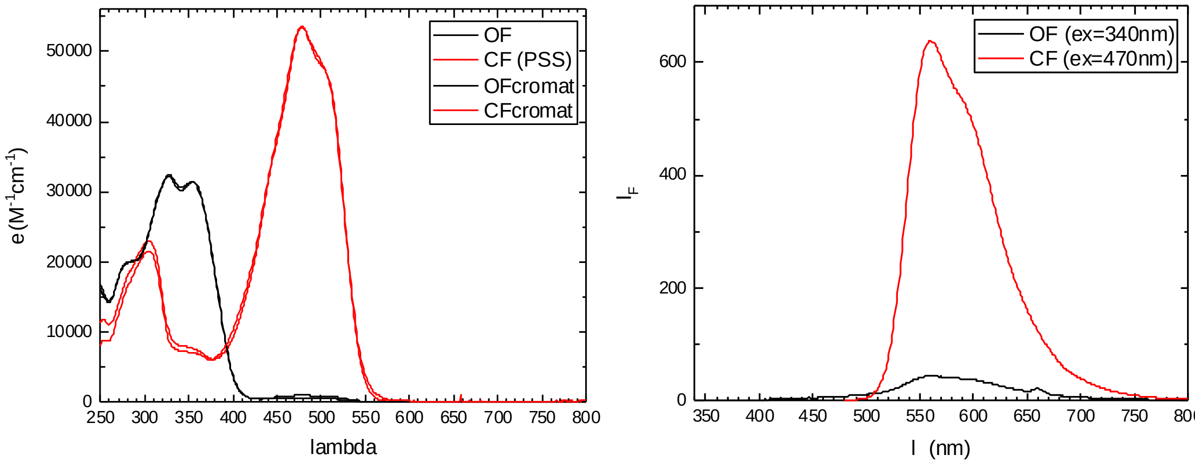

Figure S38: Absorption (left) and emission (right) spectra of compound **SyOTh$_1$** in MeCN, as the open form (OF) and the closed form (CF); in this case, the photo-stationary contains only the CF (conversion was complete). The spectrum of the OF and the CF obtained from the corresponding peaks on the chromatogram is also presented.

Compound $AsTh_2$

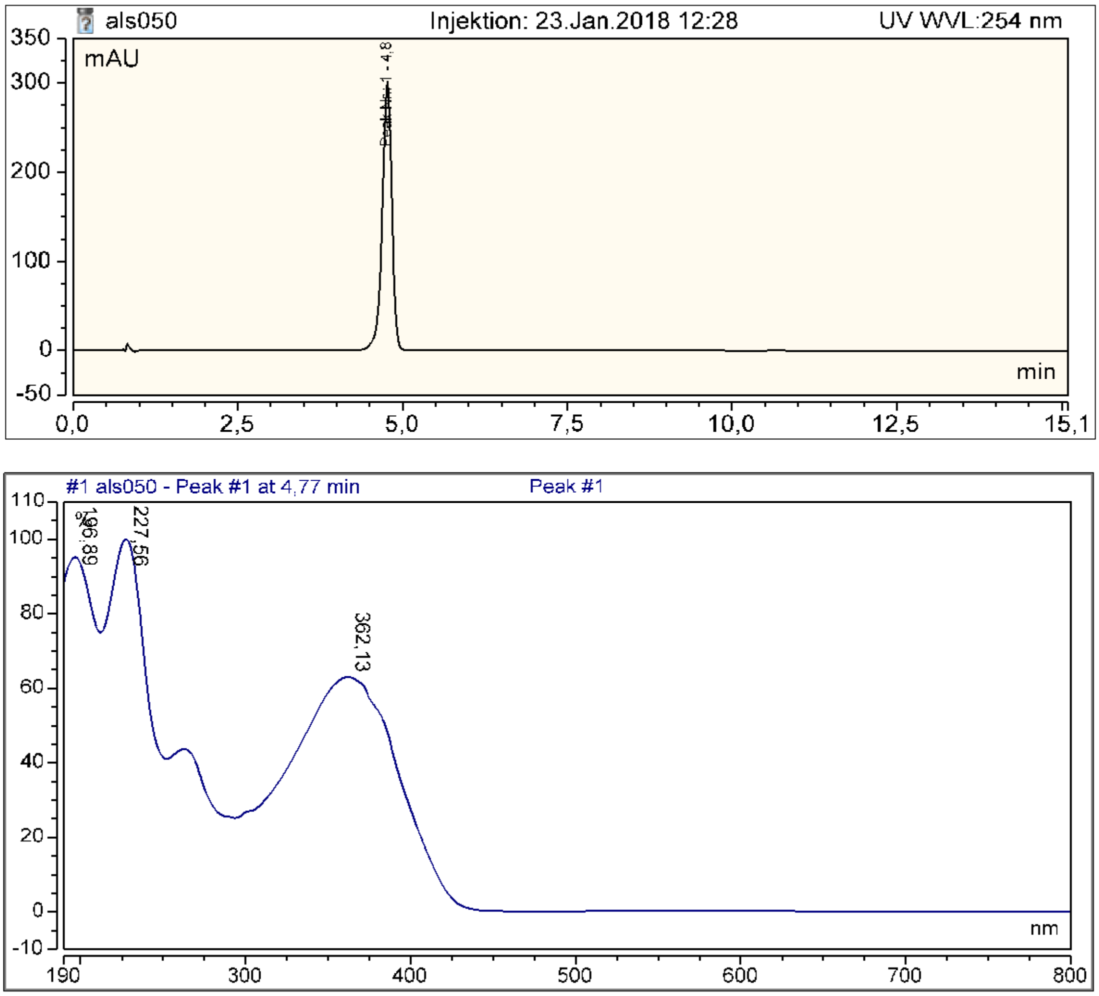

Figure S39: Analytical HPLC trace of compound **$AsTh_2$**.

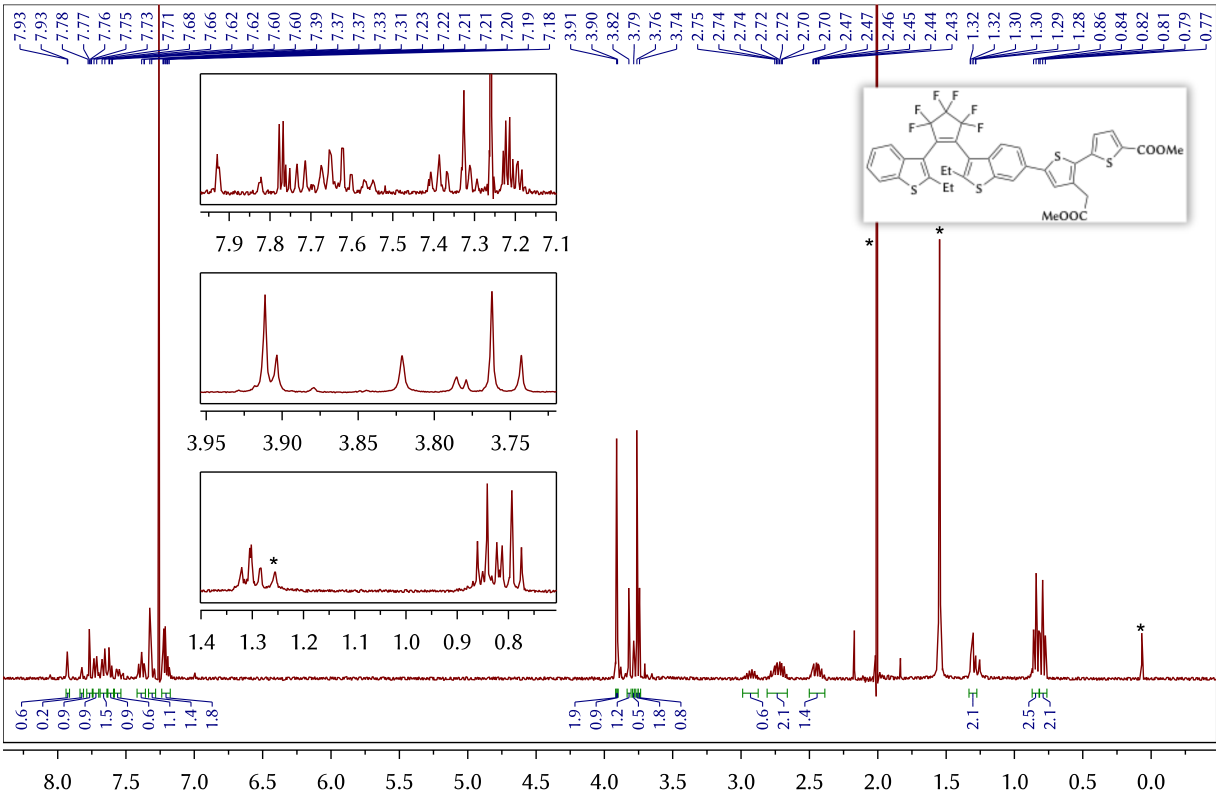

Figure S40: ^{1}H NMR spectrum of compound **AsTh$_2$** in $CDCl_3$. Asterisks denote signals of TMS, grease, water, and acetonitrile.

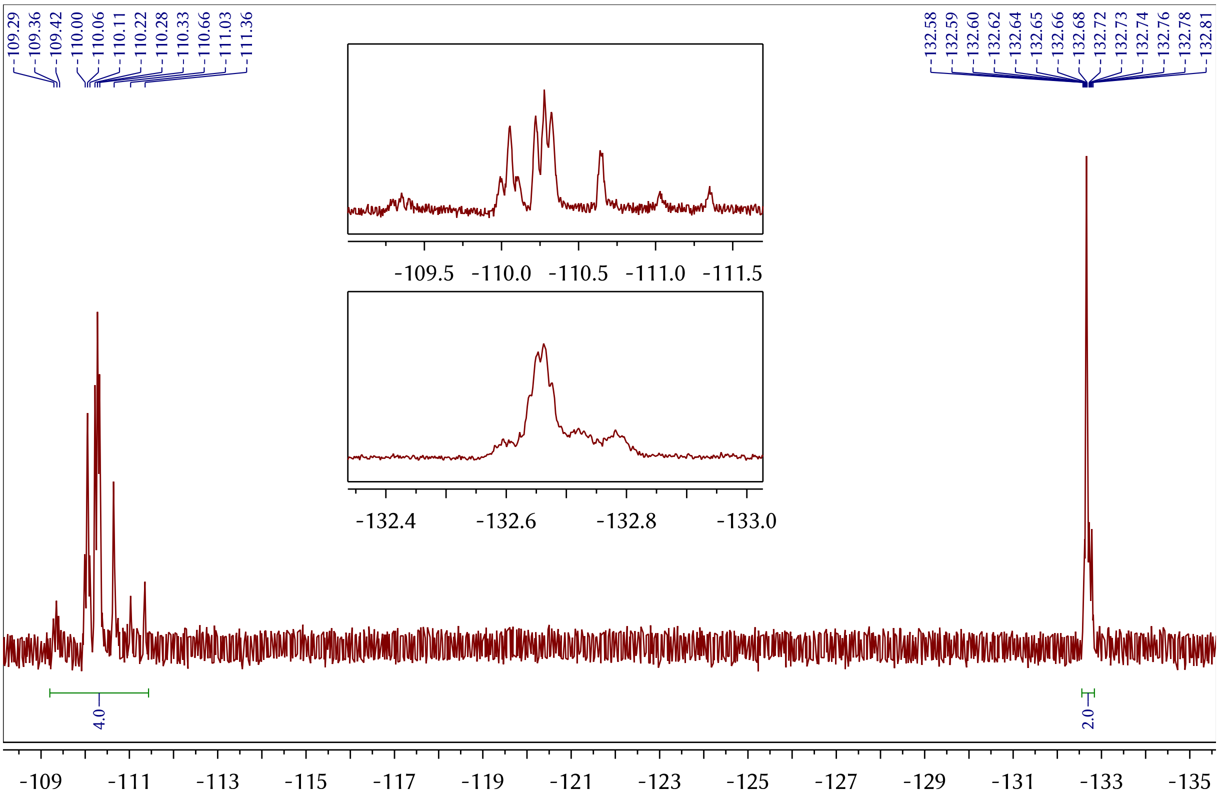

Figure S41: ^{19}F NMR spectrum of compound **AsTh$_2$** in $CDCl_3$.

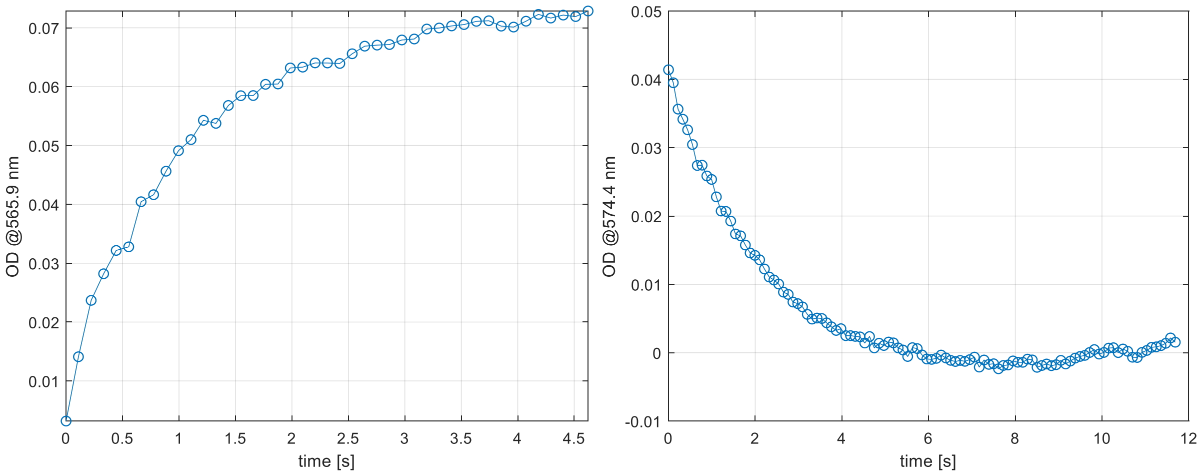

Figure S42: Absorption of **AsTh₂** in MeCN at ca. 570 nm during the cyclization (left, irradiation at 365 nm) and the cycloreversion (right, irradiation at 470 nm).

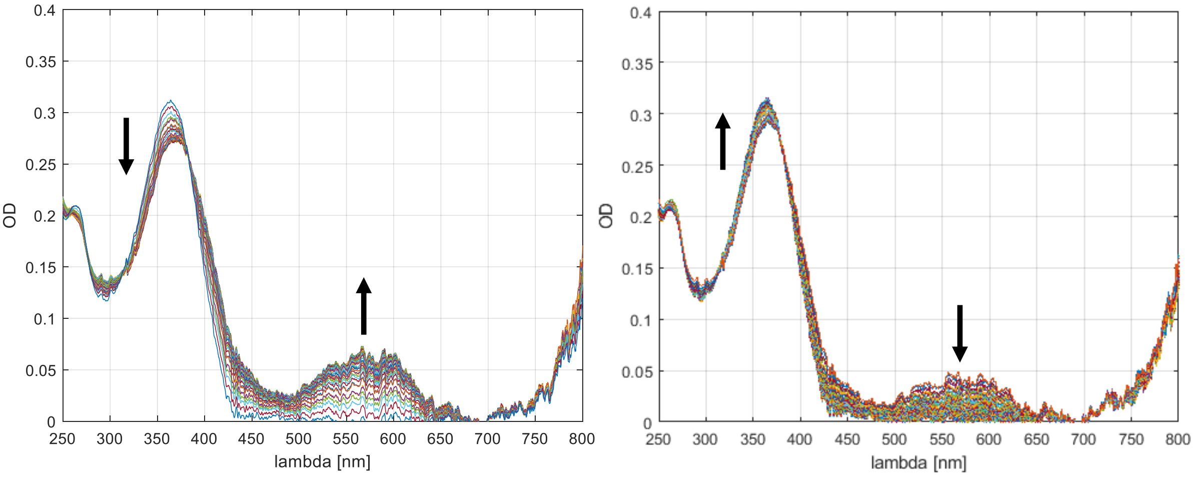

Figure S43: Absorption of **AsTh₂** in MeCN during the cyclization (left, irradiation at 365 nm) and the cycloreversion (right, irradiation at 470 nm).

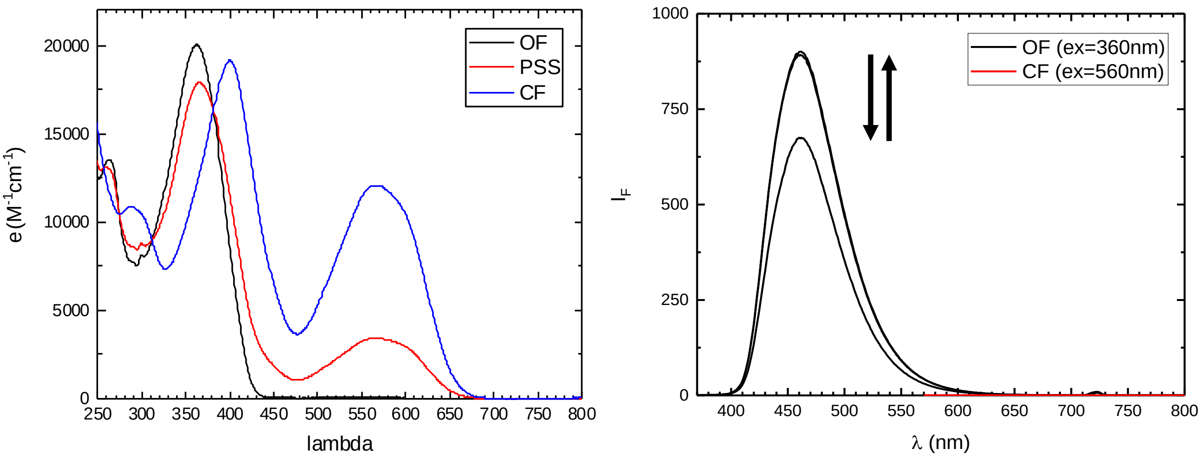

Figure S44: Absorption (left) and emission (right) spectra of compound **AsTh₂** in MeCN, as the open form (OF), closed form (CF), and the photo-stationary state at 365 nm (PSS).

Compound SyTh$_2$

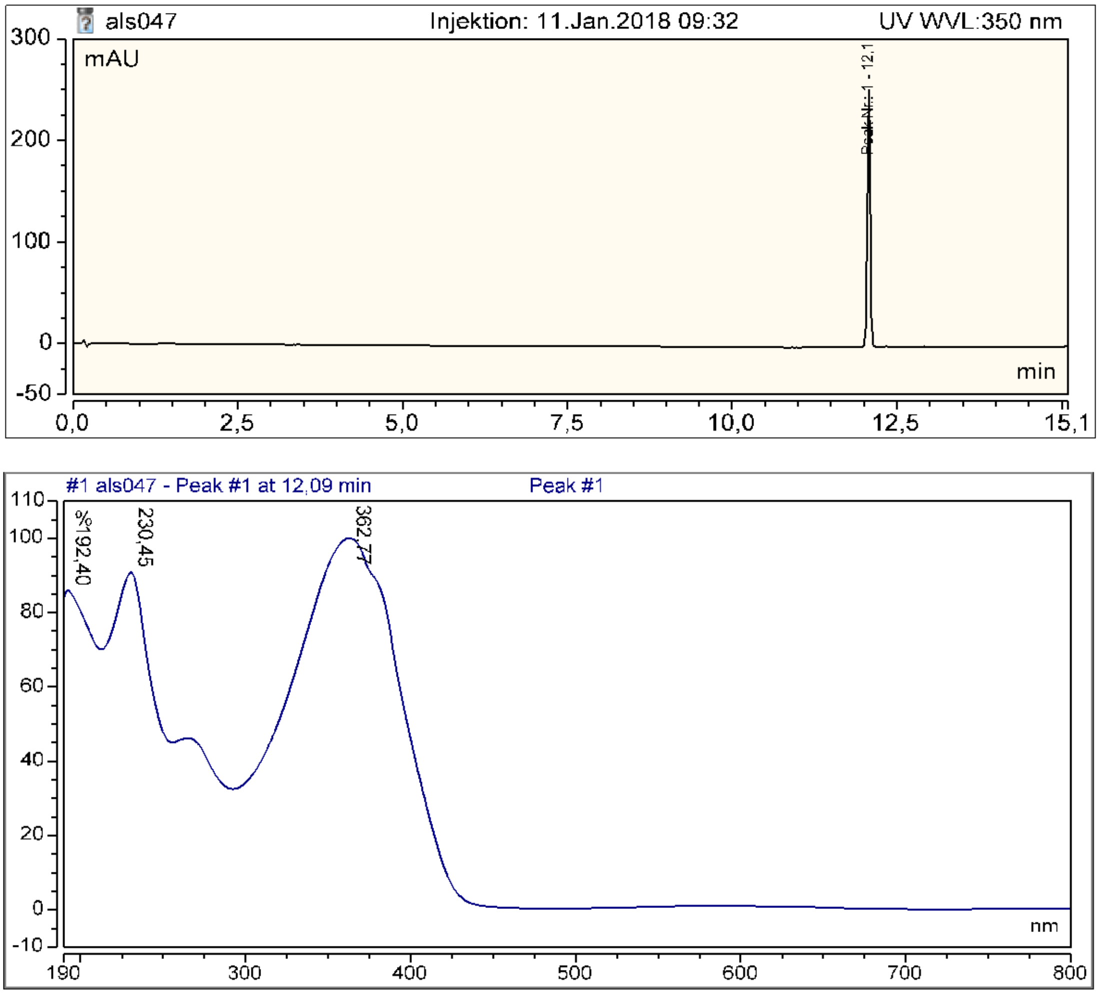

Figure S45: Analytical HPLC trace of compound **SyTh$_2$**.

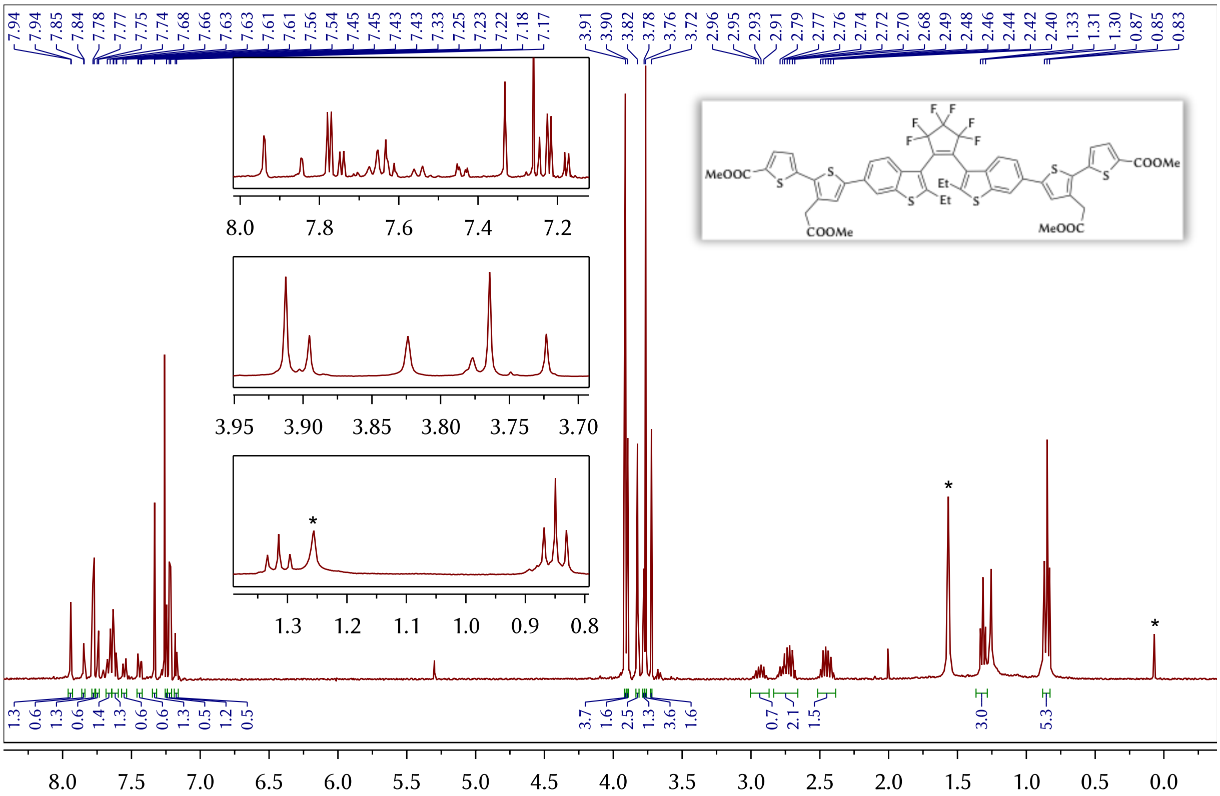

Figure S46: ^{1}H NMR spectrum of compound **SyTh$_2$** in $CDCl_3$. Asterisks denote signals of TMS, grease, and water.

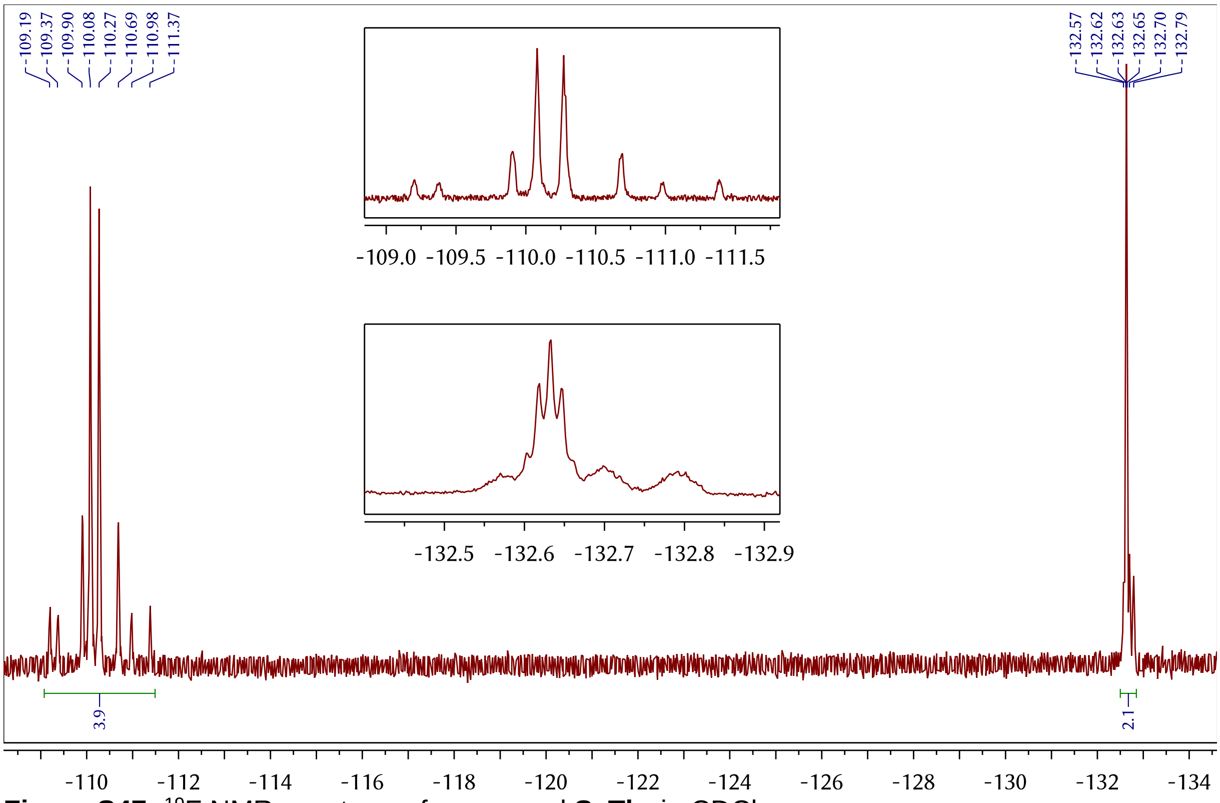

Figure S47: ^{19}F NMR spectrum of compound **SyTh$_2$** in $CDCl_3$.

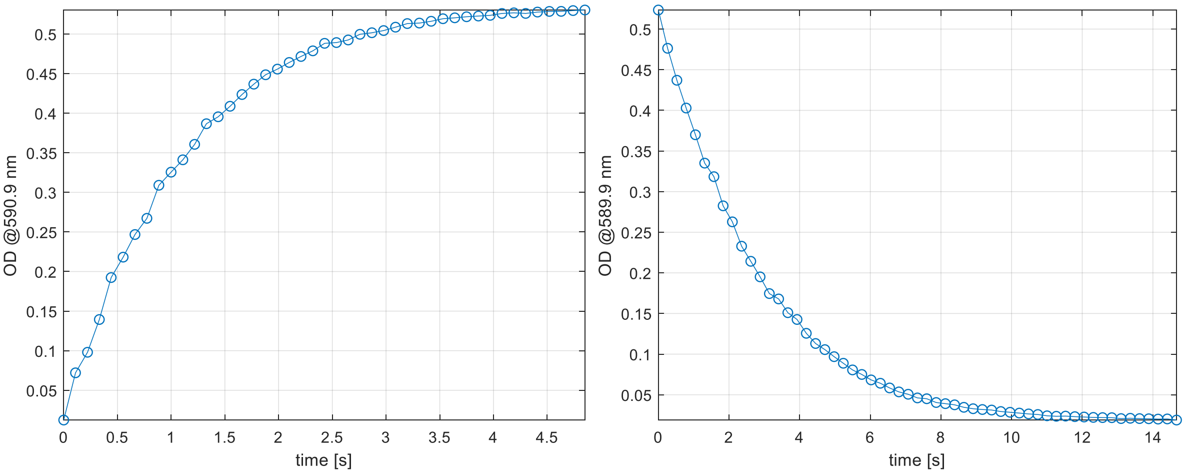

Figure S48: Absorption of **SyTh$_2$** in MeCN at ca. 590 nm during the cyclization (left, irradiation at 365 nm) and the cycloreversion (right, irradiation at 470 nm).

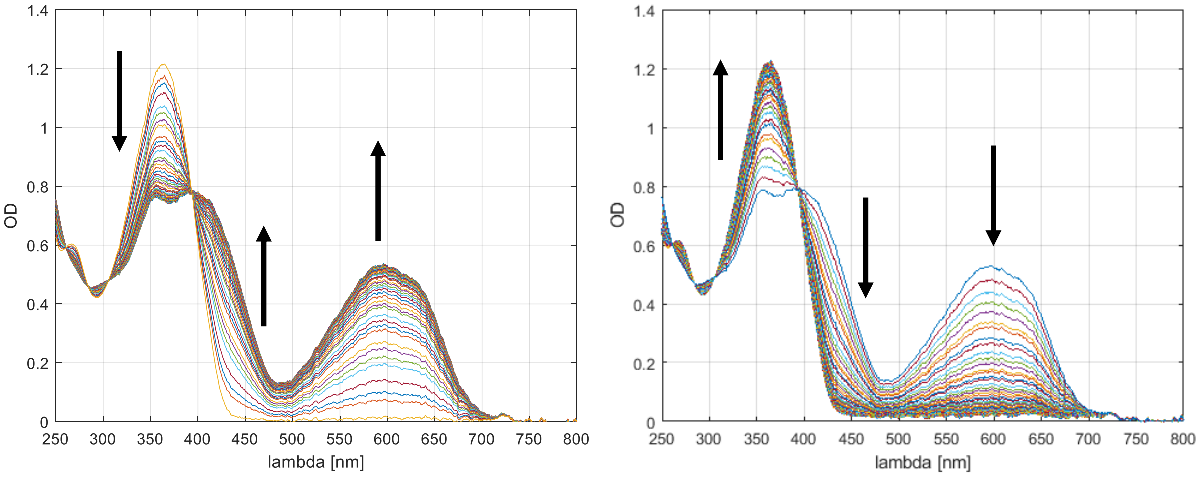

Figure S49: Absorption of **SyTh$_2$** in MeCN during the cyclization (left, irradiation at 365 nm) and the cycloreversion (right, irradiation at 470 nm).

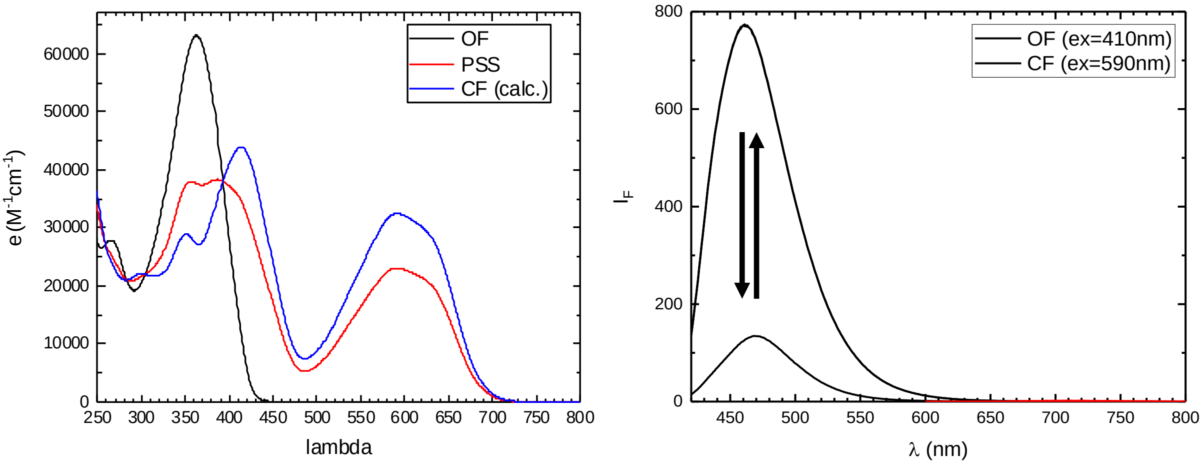

Figure S50: Absorption (left) and emission (right) spectra of compound **SyTh$_2$** in MeCN, as the open form (OF), closed form (CF), and the photo-stationary state at 365 nm (PSS).

Compound AsOTh$_2$

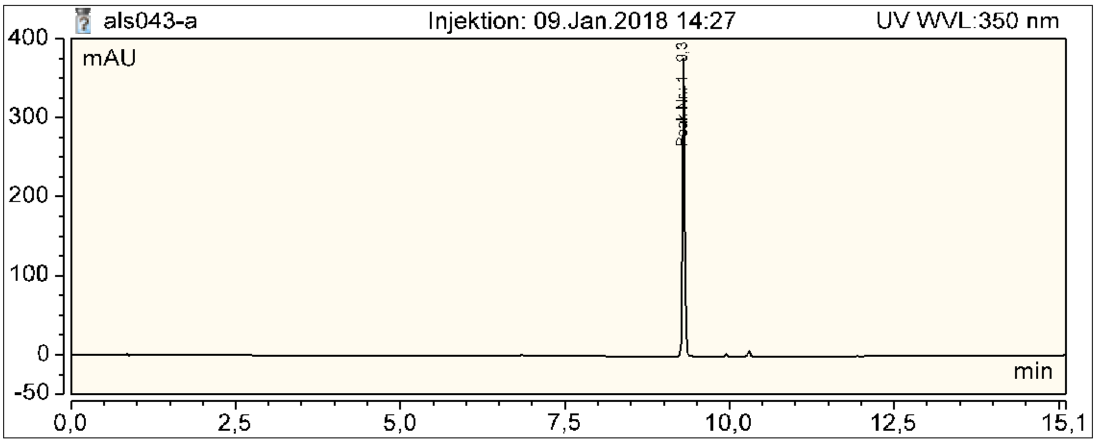

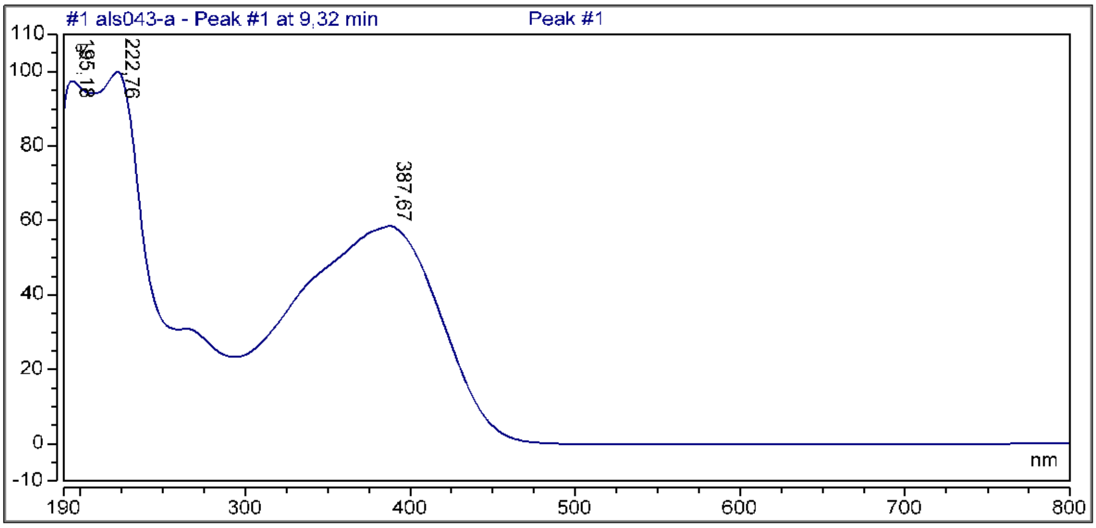

Figure S51: Analytical HPLC trace of compound **AsOTh$_2$**.

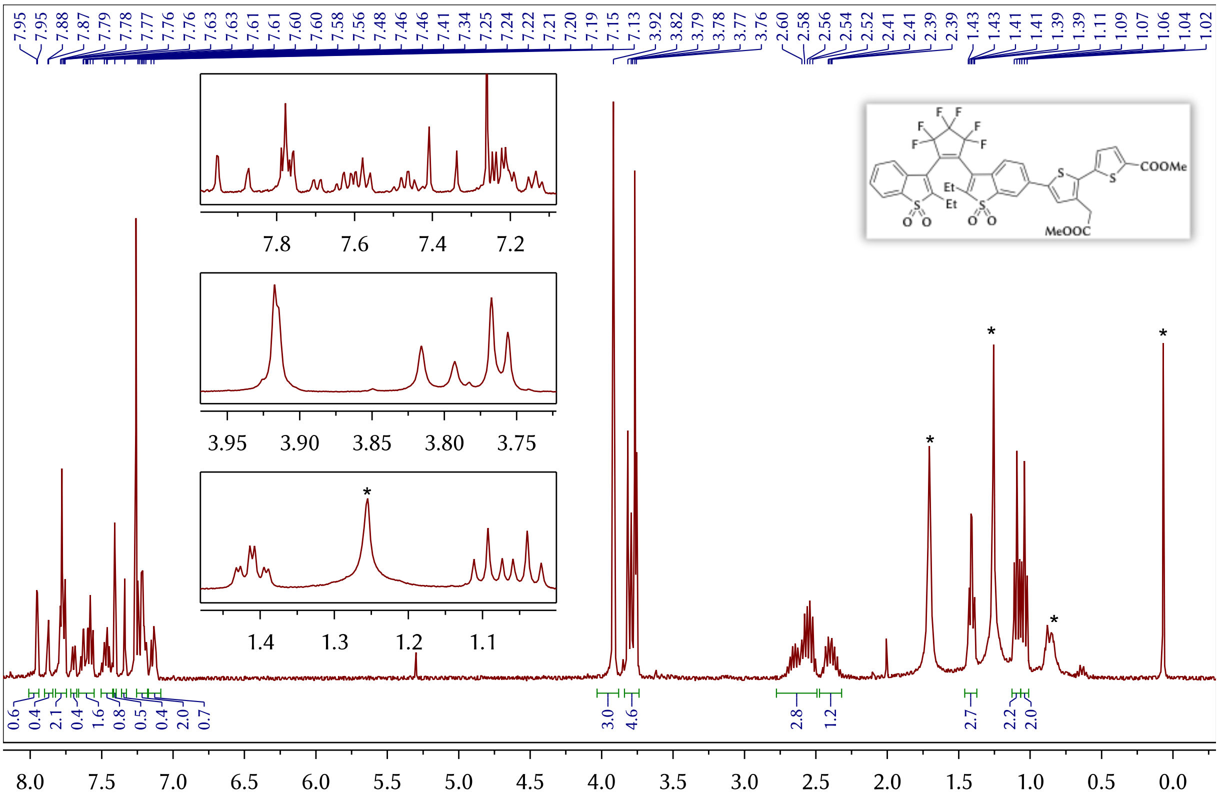

Figure S52: ^{1}H NMR spectrum of compound **AsOTh$_2$** in $CDCl_3$. Asterisks denote signals of TMS, grease, and water.

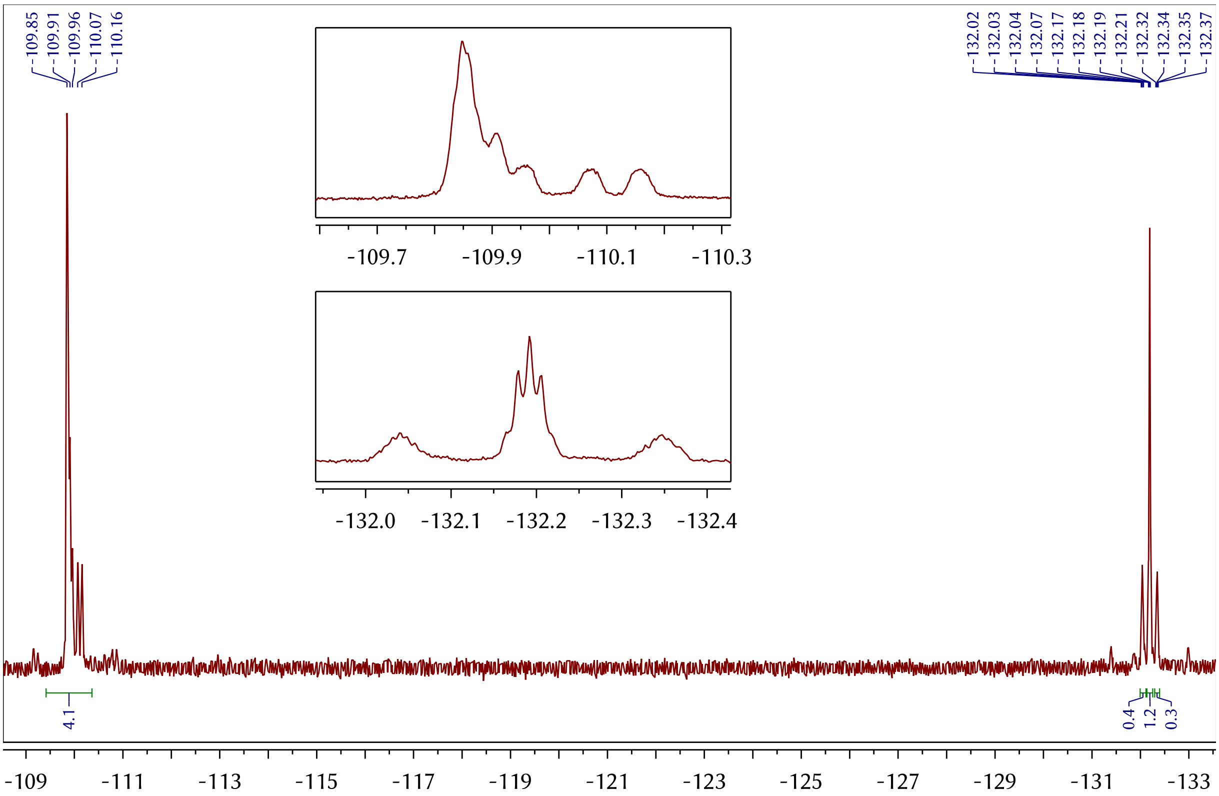

Figure S53: ^{19}F NMR spectrum of compound **AsOTh$_2$** in $CDCl_3$.

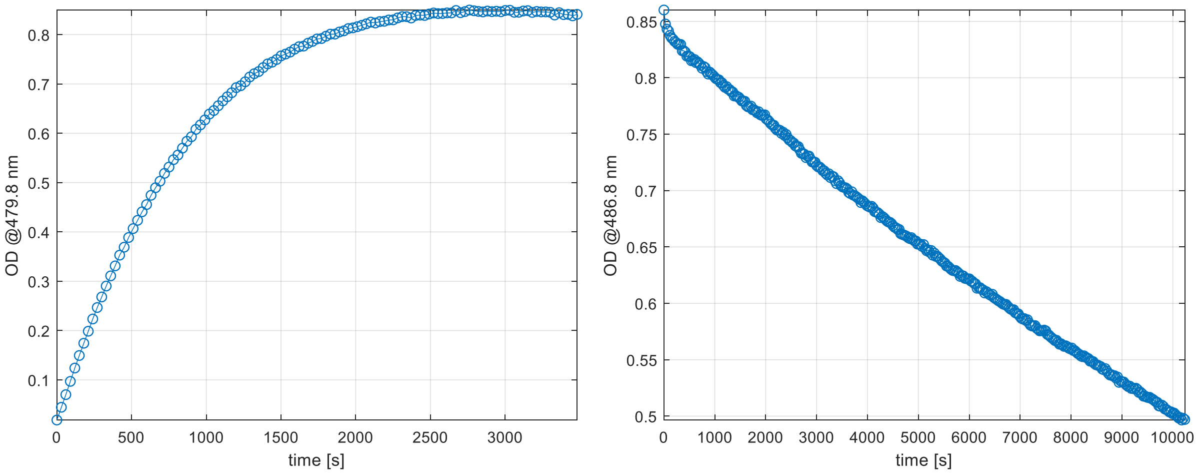

Figure S54: Absorption of **AsOTh$_2$** in MeCN at ca. 480 nm during the cyclization (left, irradiation at 365 nm) and the cycloreversion (right, irradiation at 470 nm).

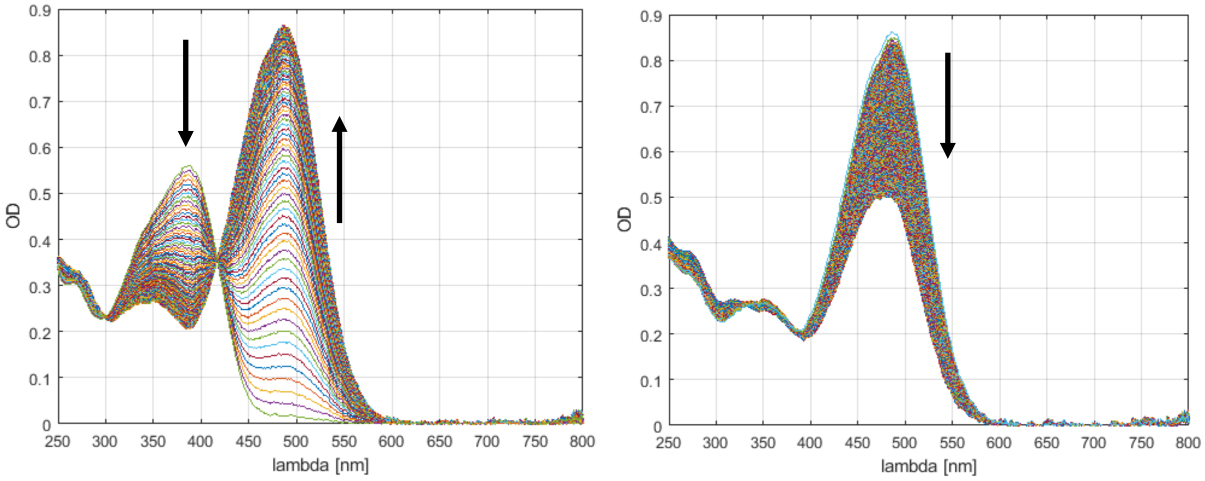

Figure S55: Absorption of **AsOTh$_2$** in MeCN during the cyclization (left, irradiation at 365 nm) and the cycloreversion (right, irradiation at 470 nm).

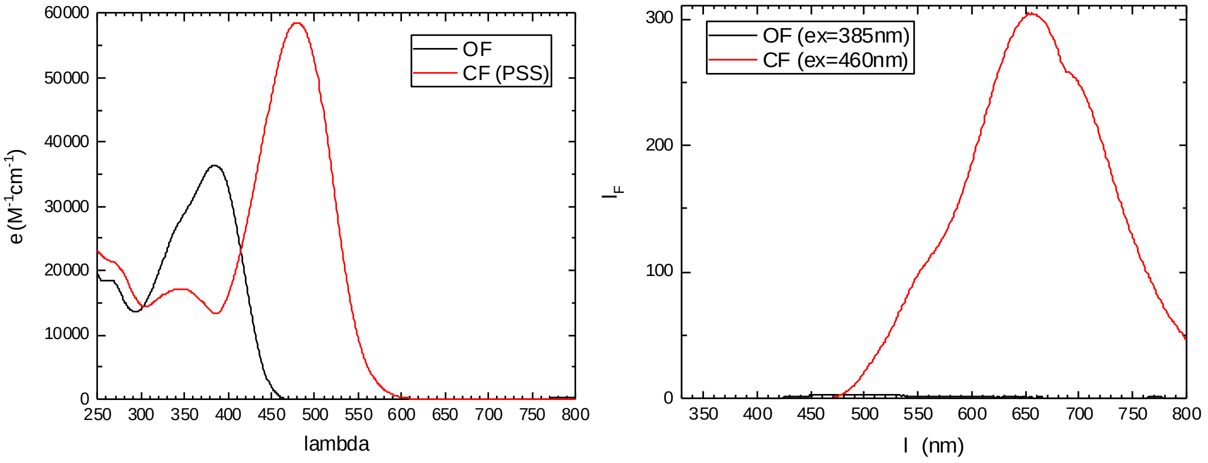

Figure S56: Absorption (left) and emission (right) spectra of compound **AsOTh$_2$** in MeCN, as the open form (OF), and the closed form (CF); in this case, the photo-stationary state contains only the CF (conversion was complete).

Compound $SyOTh_2$

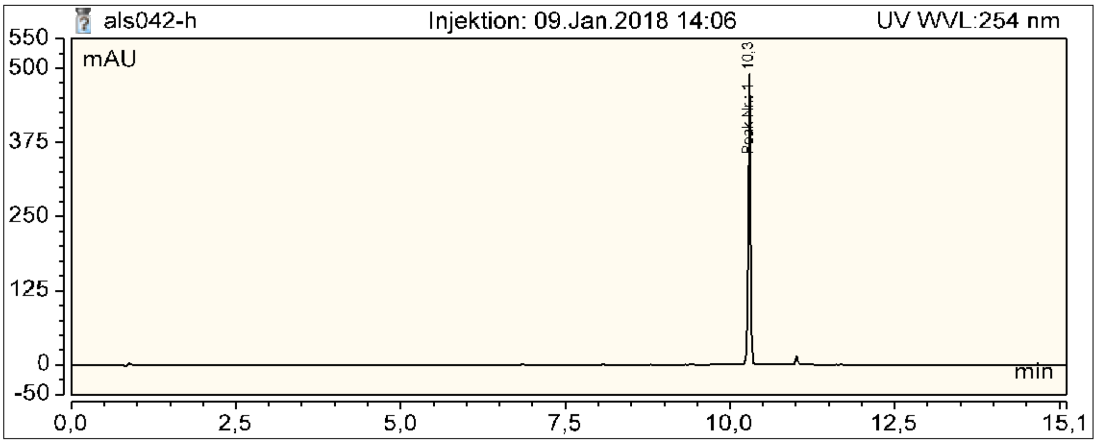

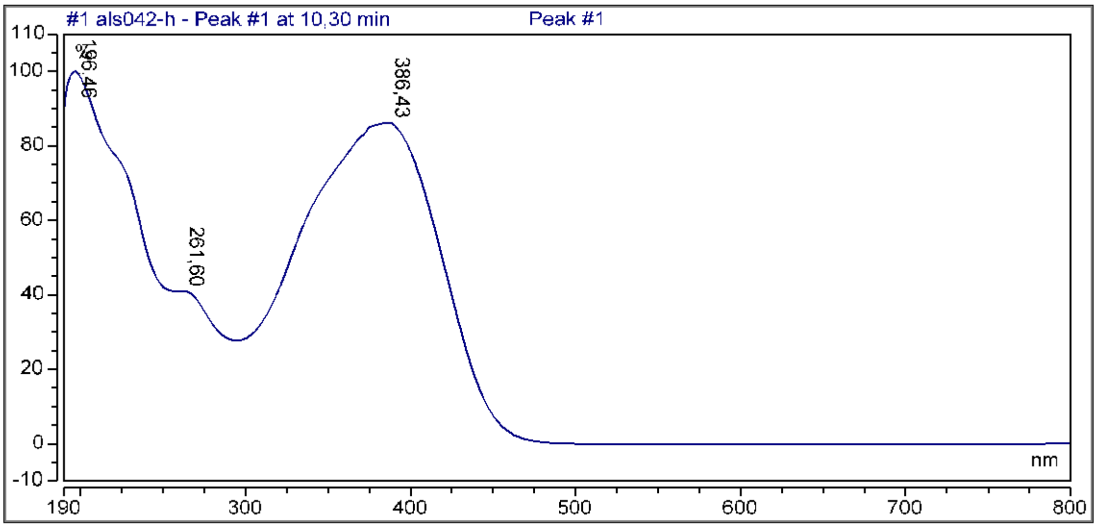

Figure S57: Analytical HPLC trace of compound **$SyOTh_2$**.

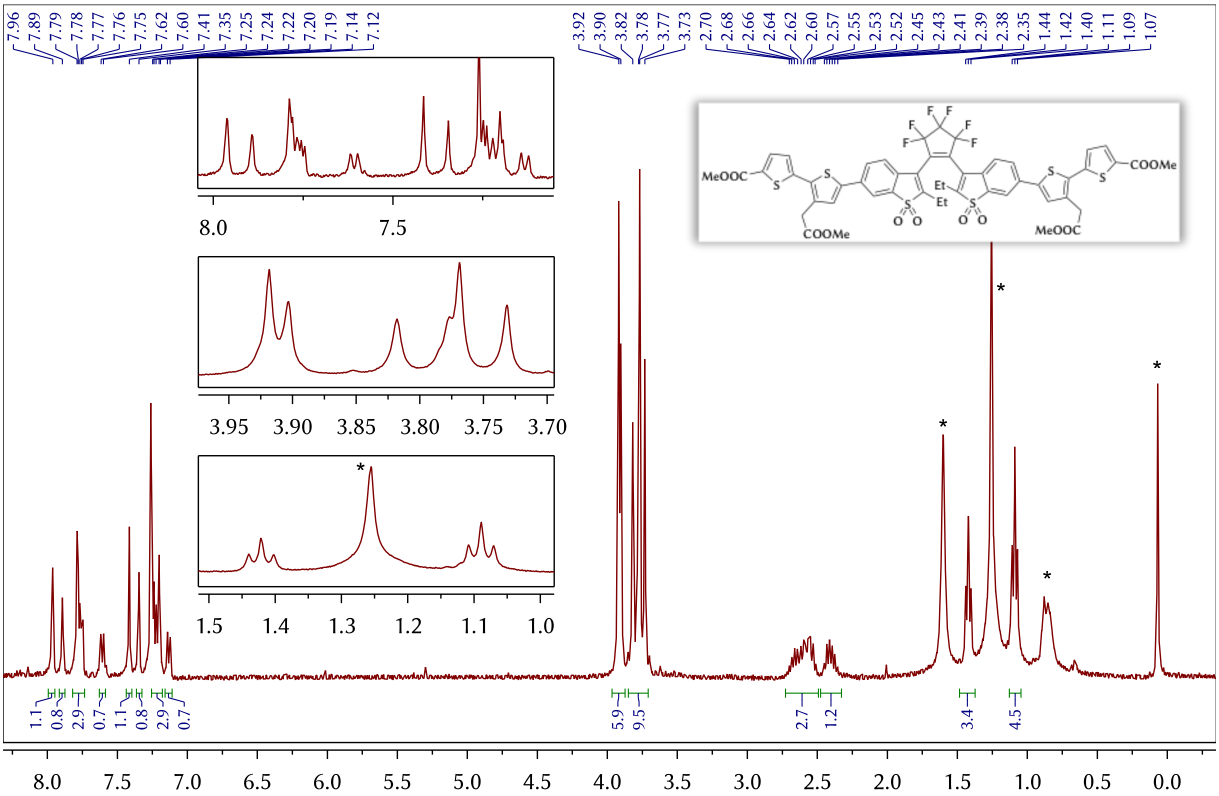

Figure S58: ^{1}H NMR spectrum of compound **SyOTh$_2$** in $CDCl_3$. Asterisks denote signals of TMS, grease, and water.

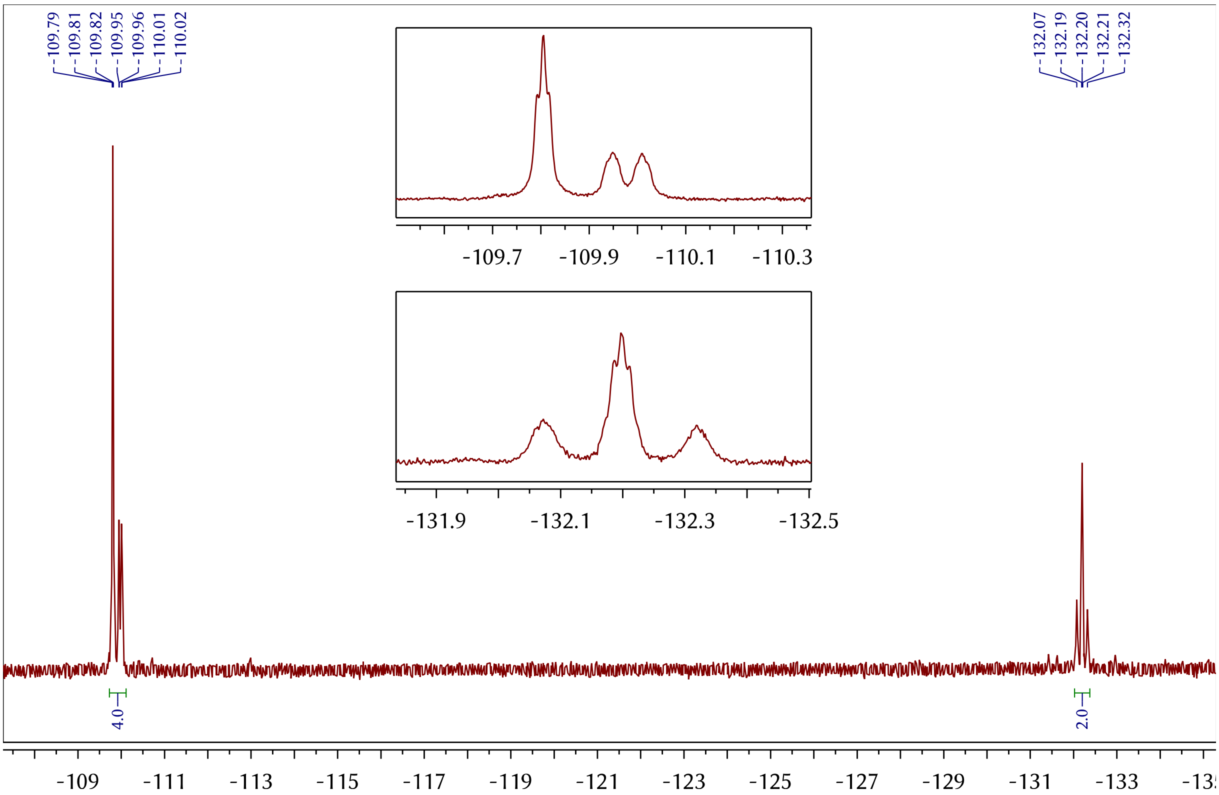

Figure S59: ^{19}F NMR spectrum of compound **SyOTh$_2$** in $CDCl_3$.

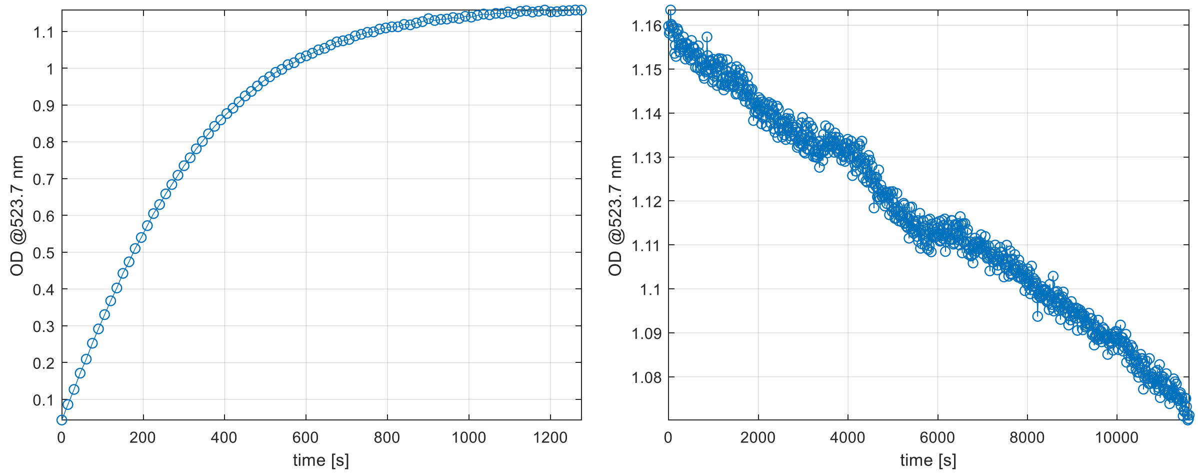

Figure S60: Absorption of **SyOTh₂** in MeCN at ca. 520 nm during the cyclization (left, irradiation at 365 nm) and the cycloreversion (right, irradiation at 470 nm).

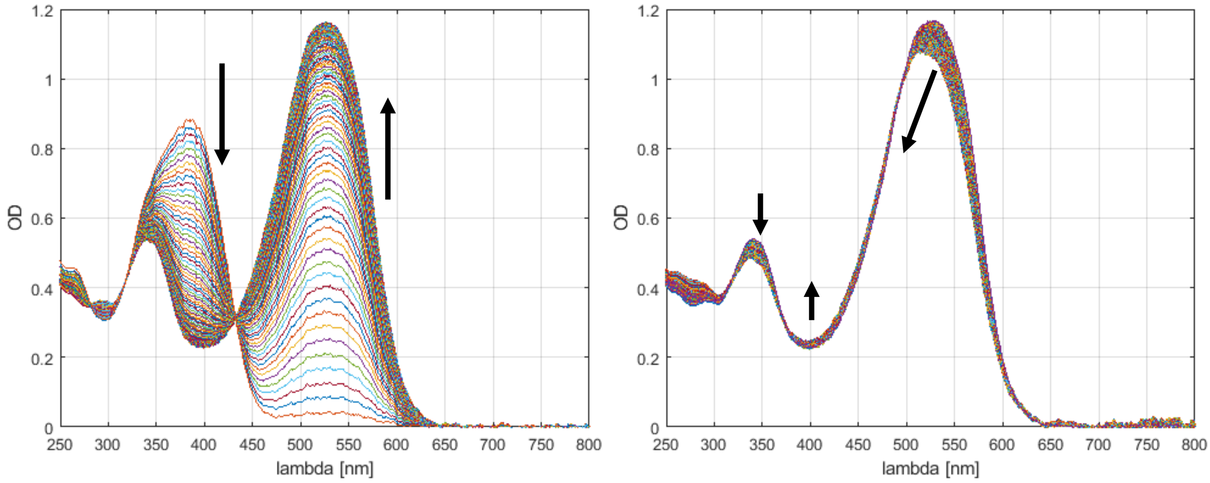

Figure S61: Absorption of **SyOTh₂** in MeCN during the cyclization (left, irradiation at 365 nm) and the cycloreversion (right, irradiation at 470 nm).

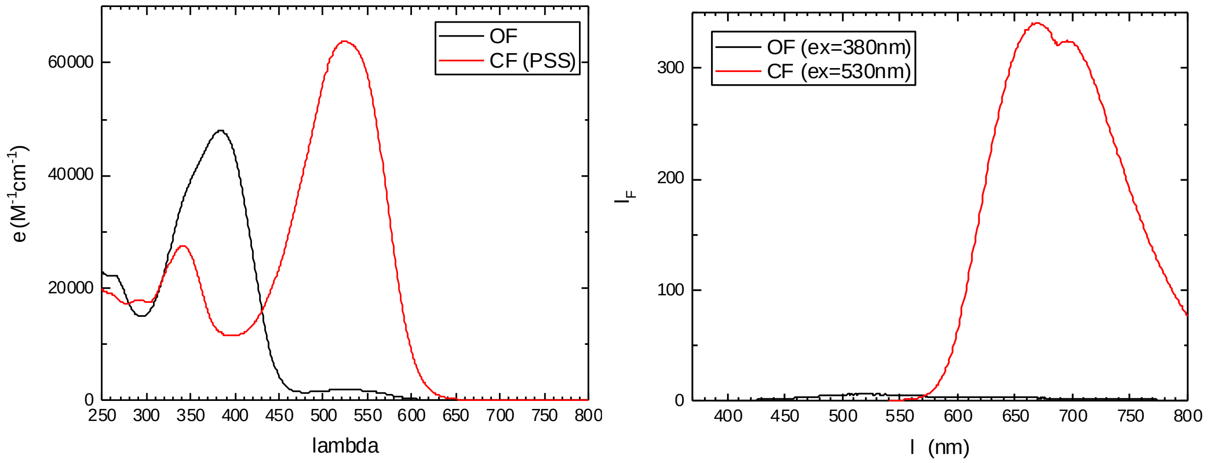

Figure S62: Absorption (left) and emission (right) spectra of compound **SyOTh₂** in MeCN, as the open form (OF) and the closed form (CF); in this case, the photo-stationary state contains only the CF (conversion was complete).

Compound SyTh$_2$-H

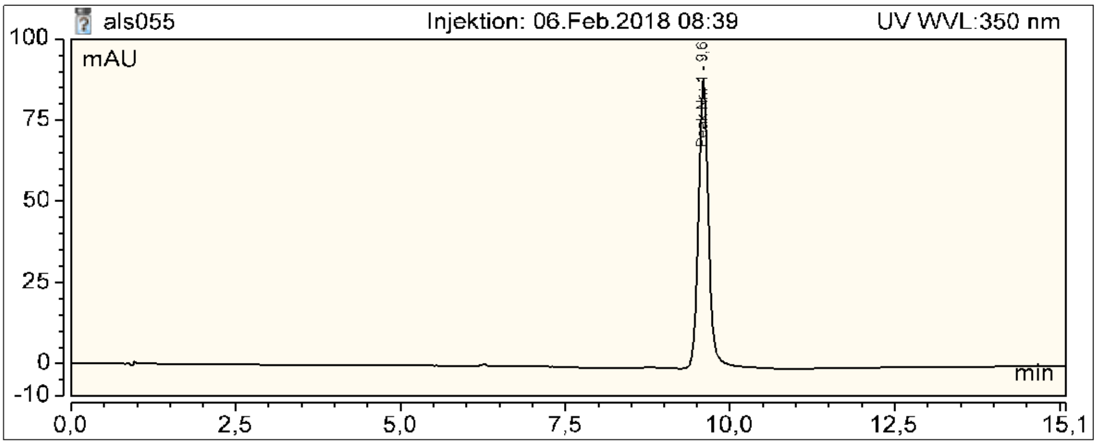

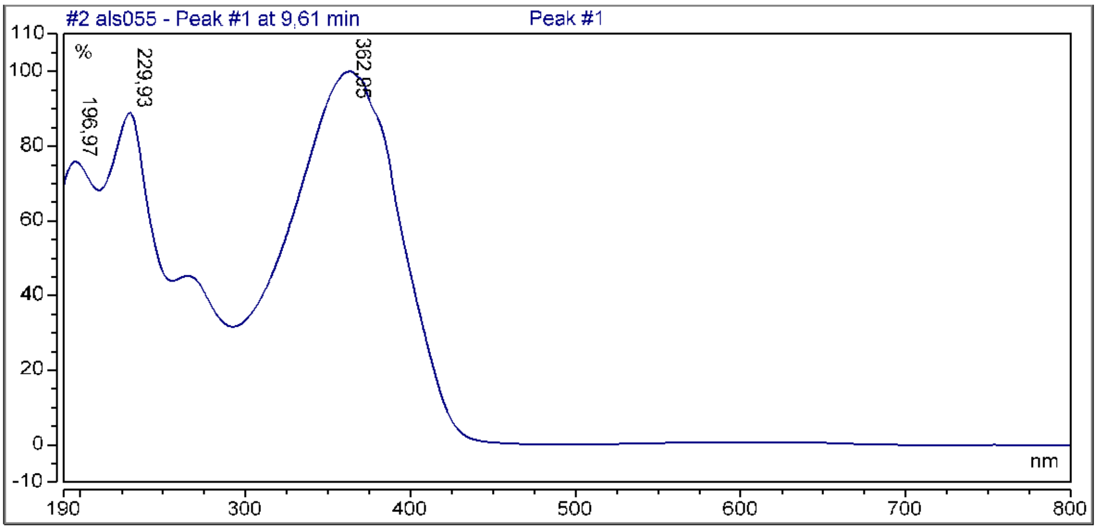

Figure S63: Analytical HPLC trace of compound **SyTh$_2$-H**.

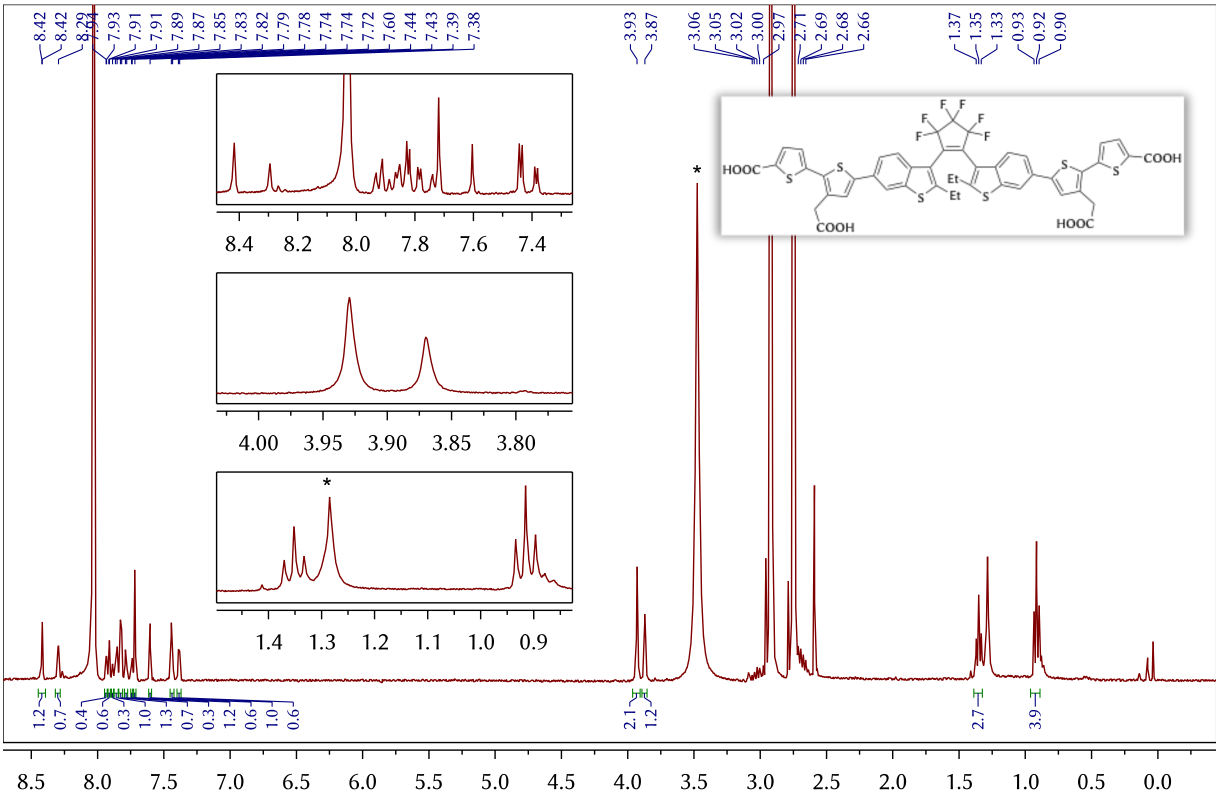

Figure S64: ^{1}H NMR spectrum of compound **SyTh$_2$-H** in DMF-d_7. Asterisks denote the signals of grease and water. Signal of the residual protons of the solvent: 2.7, 2.9 and 8 ppm.

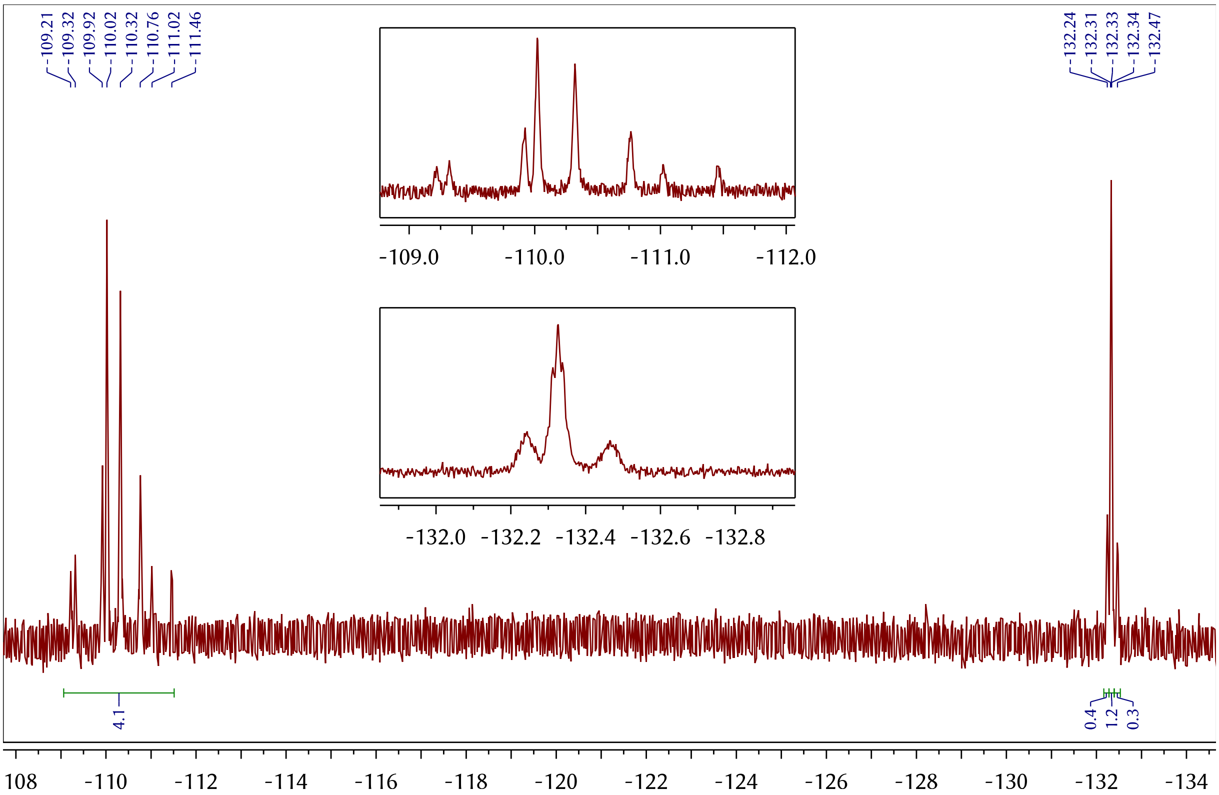

Figure S65: ^{19}F NMR spectra of compound **SyTh$_2$-H** in DMF-d_7.

References

[1] Gauglitz, G.; Hubig, S. *J. Photochem.,* 1985, *30*, 121-125.

[2] Yokoyama, Y.; Inoue, T.; Yokoyama, M.; Goto, T.; Iwai, T.; Kera, N.; Hitomi, I.; Kurita, Y. *B. Chem. Soc. Jpn.,* 1994, *67,* 3297-3303.

[3] Brouwer, A. M. *Pure Appl. Chem.,* 2011, *83,* 2213-2228.

[4] Bryan, C. S.; Braunger, J. A.; Lautens, M. *Angew. Chem. Int. Ed.,* 2009, *48,* 7064-7068; *Angew. Chem.,* 2009, *121,* 7198-7202.

[5] Pu, S.; Li, M.; Fan, C.; Liu, G.; Shen, L. *J. Mol. Struct.,* 2009, *919,* 100-111.

[6] Yamaguchi, T.; Irie, M. *J. Photochem. Photobiol., A* 2006, *178*, 162-169.

[7] K. Uno; K.; Niikura; H.; Morimoto, M.; Ishibashi, Y.; Miyasaka, H.; Irie, M. *J. Am. Chem. Soc.,* 2011, *133*, 13558-13564.

[8] Chotana, G. A.; Kallepalli, V. A.; Maleczka, R. E.; Smith, M. R. *Tetrahedron,* 2008, *64*, 6103-6114.

[9] Tang, J.; Park, J. G.; Millard, C. B.; Schmidt, J. J.; Pang, Y.-P. *PLOS ONE,* 2007, *2,* e761.

[10] Simon, R. A.; Shirani, H.; Åslund, K. O. A.; Bäck, M.; Haroutunian, V.; Gandy, S.; Nilsson, K. P. R. *Chem. Eur. J.,* 2014, *20*, 12537-12543.

[11] Klingstedt, T.; Shirani, H.; Mahler, J.; Wegenast-Braun, B. M.; Nyström, S.; Goedert, M.; Jucker, M.; Nilsson, K. P. R. *Chem. Eur. J.,* 2015, *21*, 9072-9082.
